# Structure-Based Design of New Series of Sulfonates with Potent and Specific BChE Inhibition and Anti-Inflammatory Effects

**DOI:** 10.3390/ijms27073109

**Published:** 2026-03-29

**Authors:** Siva Hariprasad Kurma, Camila Adarvez-Feresin, Oscar Parravicini, Adriana Garro, Sarka Stepankova, Jan Hosek, Karel Pauk, Jovana Lisicic, Josef Jampilek, Ricardo Daniel Enriz, Ales Imramovsky

**Affiliations:** 1 Institute of Organic Chemistry and Technology, Faculty of Chemical Technology, University of Pardubice, Studentska 573, 532 10 Pardubice, Czech Republic; sivahari1415@gmail.com (S.H.K.); karel.pauk@upce.cz (K.P.); 2 Instituto Multidisciplinario de Investigaciones Biológicas (IMIBIO-SL), Facultad de Química, Bioquímica y Farmacia, Universidad Nacional de San Luis, Chacabuco 915, San Luis 5700, Argentina; camilaadarvezferesin@gmail.com (C.A.-F.); oparravicini@gmail.com (O.P.); denriz@unsl.edu.ar (R.D.E.); 3 Department of Biological and Biochemical Sciences, Faculty of Chemical Technology, University of Pardubice, Studentska 573, 532 10 Pardubice, Czech Republic; sarka.stepankova@upce.cz; 4 Department of Pharmacology and Toxicology, Veterinary Research Institute, Hudcova 296/70, 621 00 Brno, Czech Republic; hosek.jan@vri.cz (J.H.); 507401@mail.muni.cz (J.L.); 5 Department of Molecular Pharmacy, Faculty of Pharmacy, Masaryk University, Palackeho tr. 1946/1, 612 00 Brno, Czech Republic; 6 Department of Chemical Biology, Faculty of Science, Palacky University Olomouc, Slechtitelu 27, 779 00 Olomouc, Czech Republic; josef.jampilek@gmail.com; 7 Institute of Chemistry, University of Silesia, Szkolna 9, 40-007 Katowice, Poland

**Keywords:** Alzheimer’s disease, anti-inflammatory activity, BChE activity, molecular modeling, QTAIM calculations, sulfonate derivatives

## Abstract

In the present work, a novel series of eleven sulfonate derivatives with potent inhibitory activity against butyrylcholinesterase (BChE) is reported. Of these, compounds 2-[(*E*)-(2-Benzoylhydrazinylidene)methyl]phenyl 5-(dimethylamino)naphthalene-1-sulfonate (**5c**, IC_50_ = 1.11 µM) and *tert*-butyl (2*E*)-2-[(2-{[5-(dimethylamino)naphthalene-1-sulfonyl]oxy}phenyl)methylidene]hydrazine-1-carboxylate (**5b**, IC_50_ = 11.51 µM) exhibit stronger inhibitory activity than rivastigmine, the reference compound, and exhibit high selectivity for BChE over AChE (e.g., selectivity index 57 for **5c**). Interestingly, compound **5c** also exhibited anti-inflammatory effects, which is important for potential therapeutic applications, especially in Alzheimer’s disease. These new compounds were designed through a structure-based approach using molecular modeling techniques (docking, molecular dynamic (MD) simulations, and QTAIM (quantum theory of atoms in molecules) calculations). The most promising compounds show no detectable toxic effects and satisfy Lipinski’s rule of five, indicating that they represent attractive starting structures for the design of new derivatives acting as specific BChE inhibitors. In addition, our results indicate that relatively simple computational techniques such as docking calculations and toxicity prediction programs can be valuable when properly used in the search of new candidates for this particular target. Docking calculations show that the more active compounds of this series reach the bottom region of the gorge interacting with residues within the active site of BChE. However, our data further suggest that the use of more precise techniques, such as MD simulations and QTAIM analysis, is necessary to obtain detailed insight into ligand–enzyme interactions. Regarding QTAIM calculations, they demonstrate that such computations are very useful to evaluate the molecular interactions of the different molecular complexes. In summary, we report a new series of sulfonate derivatives as promising starting structures for the development of new selective BChE inhibitors.

## 1. Introduction

Butyrylcholinesterase (BChE) displays a broad array of physiological and pharmacological activities [1]; therefore, the volume of research focused on this enzyme has steadily increased over the past few decades. BChE plays important roles in the prevention and treatment of nerve agent and organophosphorus pesticide poisoning [2], as well as in cocaine addiction and drug abuse by hydrolyzing ingested cocaine into inactive substrates [3]. Recently, emerging evidence has revealed novel roles of BChE in lipid metabolism [4]. This variety of pharmacological activities confers the application of BChE as a therapeutic target for diseases, among which Alzheimer’s disease (AD) has received the most attention [5]. BChE plays a crucial, dual role in AD by contributing to both neurotransmitter dysfunction and, as the disease progresses, the development of amyloid plaques and neurofibrillary tangles, two primary pathological hallmarks of AD. As AD progresses, the decrease in acetylcholine levels increases due to increasing levels of BChE (30–60% increase in the cortex), which compensates for the loss of AChE. BChE is found within amyloid plaques and is believed to play a role in the maturation and toxicity of these plaques. Genetic variants of the BCHE gene, most notably the K-variant, further contribute to inter-individual differences in AD susceptibility, disease onset, and therapeutic response. BChE is also associated with increased neuroinflammation and is affected by iron overload, a common factor in aging and neurodegeneration [6,7,8].

Although dual cholinesterase inhibition is generally considered advantageous for the potential treatment of neurodegenerative diseases [9], selective BChE inhibitors may provide some specific benefits, including fewer adverse effects and potential neuroprotective properties [10,11,12]. The aberrantly elevated expression ratio of BChE to acetylcholinesterase (AChE) during AD progression implies that selective BChE inhibition may be especially beneficial in the middle and late stages of the disease, when BChE activity becomes predominant [6,13]. The improvement of impaired learning and memory function by BChE inhibitors further supports targeting this enzyme in AD and promotes the development of BChE-centered treatment strategies [14]. Accordingly, current inhibitor development emphasizes the importance of improving selectivity toward BChE to mitigate adverse implications associated with AChE inhibition [1].

Consequently, selective inhibition of BChE is an appealing and promising therapeutic approach for AD and has attracted considerable research interest [1].

From a structural point of view, BChE and AChE exhibit high similarity, although several interesting structural differences enable the rational design of compounds with relatively specific activities. Both enzymes share approximately 65% amino acid sequence identity and belong to the serine hydrolase family within the α-/β-hydrolases fold superfamily [15,16,17].

Both enzymes possess similar active site composition, comprising a peripheral anionic site (PAS), a 20 Å-deep hydrophobic gorge, and a catalytic anionic site (CAS) at the bottom of aromatic gorge [18,19]. The catalytic triad of the CAS region (Ser, Glu and His) is the main binding site of the substrate. In addition, the CAS region contains three auxiliary binding sites: an oxyanion hole (Gly116, Gly117 and Ala199), a hydrophobic acyl-binding pocket (Trp230, Leu286, Val288 and Phe329), and a quaternary ammonium-binding region involving Trp82 and Phe329 [20,21]. The main structural difference between AChE and BChE is the larger gorge volume of BChE, resulting from the substitution of bulky aromatic residues in AChE by smaller aliphatic amino acids in BChE, which yields approximately 300 Å^3^ of additional space, allowing the accommodation of larger substrates [22,23].

We previously reported molecular dynamics (MD) simulations indicating that the size of the pocket entrance of BChE is substantially wider than that of AChE [16]. In fact, we have recently reported the feasibility of co-occupancy of two ligands—galantamine (GAL) and donepezil (DON)—within the active sites of both enzymes, a phenomenon more favorable in BChE due to its larger gorge volume. This would result in synergistic effects between GAL and DON if administered in an appropriate ratio of concentrations [24].

Regarding the extensive efforts to develop novel BChE inhibitors, numerous compounds representing diverse structural scaffolds have been reported. Among the most interesting are: tacrine analogs [25,26,27,28,29,30], tetrahydroaminoacridine derivatives [31,32,33,34,35], carbamate-based inhibitors [36,37], imidazole, benzimidazole [38], and benzothiazole derivatives [39,40], compounds possessing indole skeleton [41], carbazole derivatives [42], tertiary amines [43], quaternary ammonium compounds [44], and sulfonamide derivatives [45], among others.

Our research group has also reported several compounds acting as cholinesterase inhibitors, including 3-(dialkylamino)-2-hydroxypropyl 4-[(alkoxy-carbonyl)amino]benzoates [46], arylaminopropanone derivatives [47], and pyrrolo [2,1-a]isoquinolinones derivatives [48]. More recently, we focused on the development of selective BChE inhibitors. Consequently, we have reported compounds obtained from both natural [49] and synthetic [16,37] sources with specific activity against BChE. In the case of natural products, bioactive alkaloids were isolated from an alkaloid-enriched extract of *Cannabis sativa* roots using a bioguided fractionation strategy [43]. For synthetic compounds, we reported a series of fourteen benzyl [2-(arylsulfamoyl)-1-substituted-ethyl]carbamates [31], which were prepared by multi-step synthesis, most of which exhibited a strong BChE inhibitory activity, with several compounds displaying lower IC_50_ values than those of the reference inhibitor. In addition, detailed information about the binding mode of these compounds to the active site of BChE was obtained by combined computational approaches, including docking, MD simulations, and QTAIM (quantum theory of atoms in molecules) analyses.

**Figure 1:**
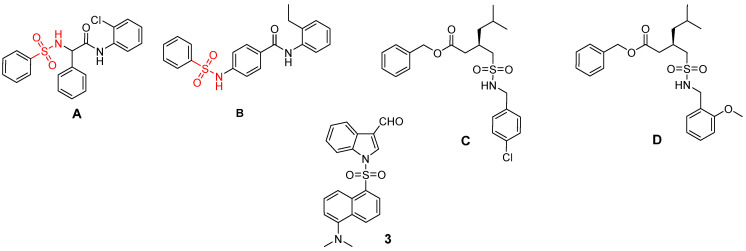
Representative BChE inhibitors. Compounds **A** and **B** are selective BChE inhibitors presented in reference [1] (where they are numbered 61, 62), while compounds **C** and **D** were reported by our group in reference [37]. Compound **3** serves as the starting scaffold for the present study.

Sun et al. [1] provided a concise review of recently reported selective anti-BChE compounds based on their chemotypes. Among them, compounds with sulfonamide functional groups come into view several times (see compounds **A** and **B** in Figure 1). In this regard, our group reported 11 sulfonamides derivatives with strong preference inhibition of BChE compared to AChE [37]. Remarkably, some of them were more active than rivastigmine [37] (see compounds **5j** and **5k** in Figure 1). Notably, these compounds possess some structural resemblance to the sulfonamide derivatives reported in reference [1]. We report here a new series of sulfonate derivatives exhibiting selective inhibitory activity against BChE. These compounds attempt to deviate from the classical design of cholinesterase inhibitors based on carbamate and/or amide moieties [50]. Several recent studies have reported that compounds containing sulfonate or sulfonyl-based structures exhibit significant enzyme inhibitory properties [51], including activity against cholinesterases. In particular, hydrazone–sulfonate hybrids and sulfonylhydrazone derivatives have been reported as promising inhibitors of acetylcholinesterase and butyrylcholinesterase [52,53,54]. The biological relevance of these compounds has been attributed, at least in part, to the presence of the sulfonyl group, which can participate in hydrogen-bonding interactions with amino acid residues in enzyme active sites and contribute to favorable conformational constraints within the molecular scaffold [55]. Our compounds were designed sequentially and optimized over time using the results obtained from a structure-based approach using molecular modeling techniques, including docking, MD simulations, and QTAIM calculations to characterize enzyme–ligand interactions in detail. The incorporation of sulfonate moieties into bioactive frameworks represents an attractive strategy for the development of new cholinesterase inhibitors, as evidenced by recent publications on molecules with similar structural motifs [53,54,56,57]. Some compounds in this new series showed significant inhibitory activity against BChE, increased selectivity towards BChE compared to AChE, and, in addition, anti-inflammatory effects of potential therapeutic interest.

## 2. Results and Discussion

### 2.1. Synthesis of Target Compounds

**Scheme 1:**
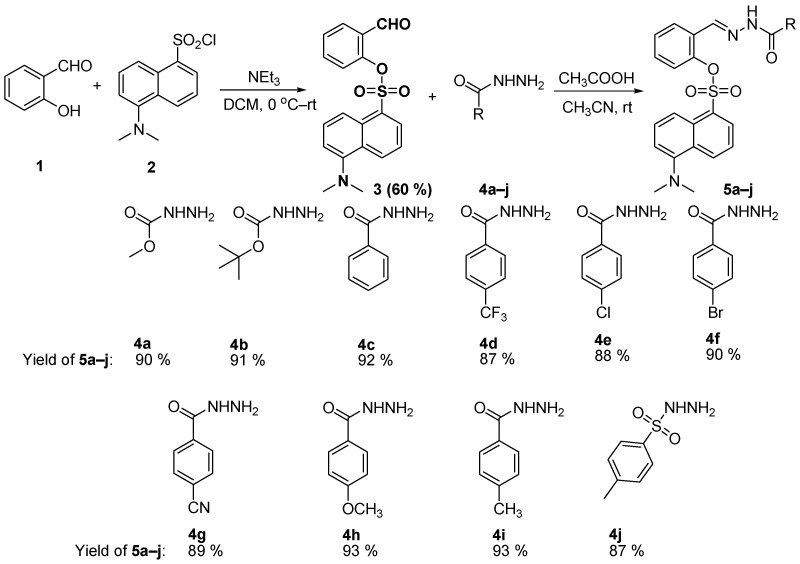
Synthesis of sulfonate intermediates **3** and target compounds **5a**–**j** from hydrazides **4a**–**j**, including reaction conditions and yields.

The synthesis of the investigated molecules was initially based on the reaction of freshly distilled salicylaldehyde **1** with commercially available 5-(dimethylamino)naphthalene-1-sulfonyl chloride **2** in dichloromethane, in the presence of an organic base (triethylamine). The reactants were combined at 0 °C, and the reaction mixture was subsequently allowed to warm to room temperature and stirred until completion. The intermediate **3** was isolated by column chromatography in a yield of 60%. Subsequent modification of compound **3** with selected hydrazides **4a**–**j** in acetonitrile, in the presence of glacial acetic acid, afforded the final compounds **5a**–**j** in excellent yields ranging from 87 to 93%. (Scheme 1) The prepared molecules were characterized using standard analytical techniques, including ^1^H and ^13^C NMR spectroscopy, high-resolution mass spectrometry (HRMS), infrared spectroscopy (IR), and elemental analysis.

### 2.2. Inhibitory Activity of Cholinesterases

Using the modified Ellman’s method, a standard approach for in vitro screening of potential cholinesterase inhibitors, the inhibitory activity of newly synthesized compounds against AChE from electric eel and BChE from horse serum was studied. The inhibitory activities of the investigated derivatives were expressed as IC_50_ values, defined as the inhibitor concentration required to reduce enzyme activity by 50%. In addition, selectivity indexes (SI), quantifying selectivity for BChE, were calculated as the ratio IC_50_(AChE)/IC_50_(BChE). RIV, a dual inhibitor of AChE and BChE, was used as a standard for comparison, see Table 1. In addition, the effects of GAL and DON are shown in Table 1.

As shown in Table 1, the IC_50_ values for BChE inhibition span a wide range, specifically from 0.74 μM (**3**) to 212 μM (**5d**). Five compounds (**3**, **5a**, **5b**, **5c**, and **5i**) showed stronger BChE inhibition than RIV under the assay conditions, with compound **3** being more than 50-fold more potent. Regarding AChE inhibition, the IC_50_ values of the studied derivatives ranged from 39.5 μM (**5i**) to 92.5 μM (**5g**). Three of the investigated compounds (**3**, **5e**, and **5i**) showed slightly stronger AChE inhibition than RIV.

Comparison of the inhibitory efficacy of key intermediate **3** with compounds **5a**–**5j**, indicates that none of the latter surpassed compound **3** in BChE inhibition. In contrast, differences in AChE inhibition between key intermediate **3** and compounds **5a**–**5j** were less pronounced. Compound **3** serves as the parent or lead structure. Its high potency and selectivity for BChE (SI = 70.55) suggest that its core fits perfectly into the larger, more flexible binding pocket of BChE compared to the narrow gorge of AChE. BChE lacks the “aromatic bottleneck” found in AChE, allowing larger core structures like those in the **5**-series to bind more effectively. Two of the studied compounds (**5e** and **5i**) are slightly more potent, while the other four compounds (**5a**, **5b**, **5c**, and **5f**) are only slightly weaker inhibitors of AChE than intermediate **3**. The effectiveness of all the mentioned compounds (i.e., **3**, **5a**, **5b**, **5c**, **5e**, **5f**, and **5i**) is essentially comparable to the effectiveness of RIV.

Regarding selectivity, most derivatives exhibited a relatively balanced inhibition of both enzymes, with SI values ranging from 0.43 to 5.78. Two compounds displayed marked selectivity for BChE: compound **5c** showed a more than 50-fold lower IC_50_ value for BChE than for AChE, while compound **3** exhibited IC_50_ values for BChE even 70-fold lower than the IC_50_ for AChE.

It is important to note that none of the investigated compounds achieves the nanomolar inhibitory effect of DON or the potent inhibition of AChE by GAL. On the other hand, derivatives **3** and **5c** are more potent BChE inhibitors than GAL and all active compounds have higher selectivity for BChE than clinically used drugs.

The type of inhibitory mechanism of the most active BChE inhibitors (**3**, **5b**, and **5c**) was also determined. The type of inhibition can be identified using the Lineweaver–Burk plot [58] and by comparing the kinetic parameters Michaelis constant (K_M_) and maximum velocity (V_m_) obtained in the absence and presence of inhibitors found for the uninhibited and inhibited reaction. The nature of the changes in K_M_ and V_m_ values, together with the intersection of the lines in the Lineweaver–Burk plot, distinguish the type of inhibition. Reversible enzyme inhibitors are classified as competitive, classical non-competitive, uncompetitive and mixed type (also referred to as mixed non-competitive inhibition).

**Figure 2:**
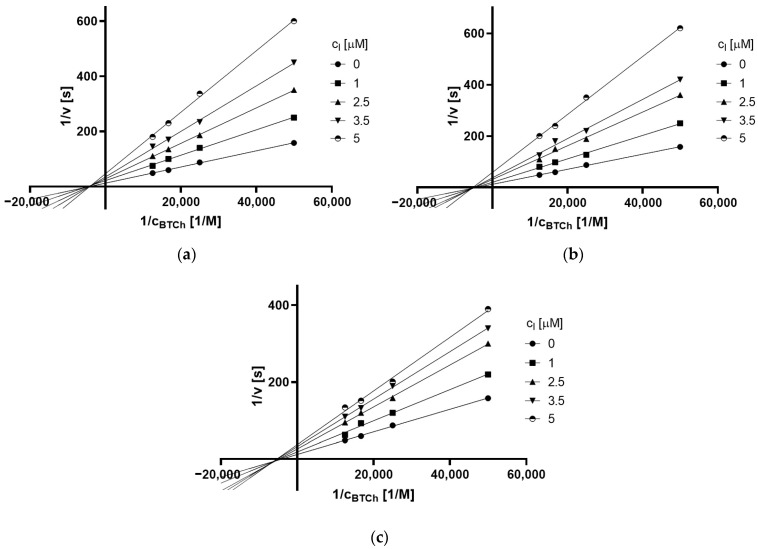
Lineweaver–Burk plots obtained for derivatives **3** (**a**), **5b** (**b**) and **5c** (**c**), respectively, inhibiting BChE. (BTCh—butyrylthiocholine).

The results are depicted in Figure 2a–c. Based on the obtained results, it is evident that compound **3** was identified as a classical non-competitive inhibitor, whereas and compounds **5b** and **5c** showed a mixed type of inhibition (Figure 2a–c).

### 2.3. Cytotoxicity and NF-κB Inhibition

**Figure 3:**
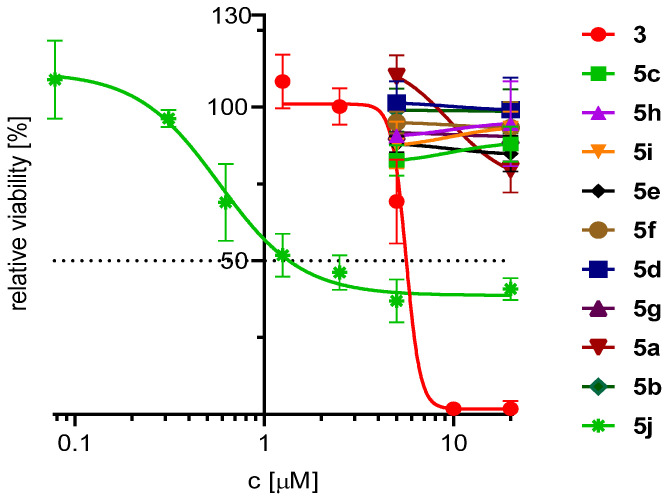
Relative viability of THP1-Blue NF-κB cells. Cells were kept in serum-free medium and incubated with the tested sulfonates for 24 h. After that, relative viability was determined by a CCK-8 kit. Graph shows means ± SEM.

All tested compounds, except **3** and **5j**, did not show a cytotoxic effect on THP1-Blue NF-κB cells (Figure 3). The IC_50_ value for compound **3** was calculated as 5.8 ± 1.1 µM. This observation is in accordance with previous experiments, where any or low cytotoxicity was described for different sulfonamides tested on several cell lines (e.g., references [59,60,61]. It indicates good medicinal and pharmacological potential for application in human therapy.

**Figure 4:**
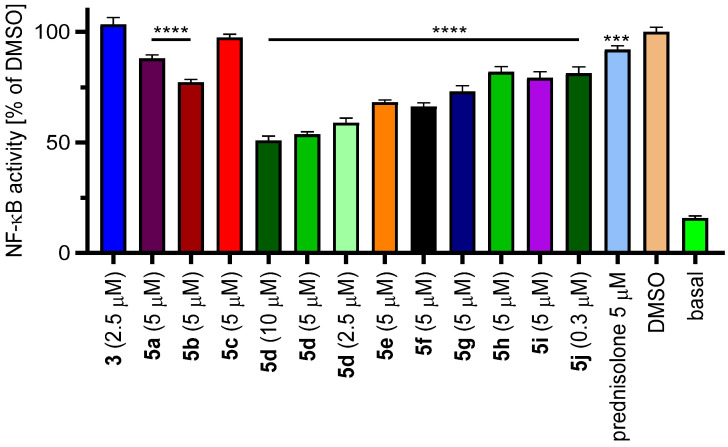
Effect of tested sulfonates on NF-κB activity in LPS-challenged THP1-Blue™ NF-κB cells. Cells were pre-treated with all the tested compounds and control drug prednisolone at the indicated concentrations, or vehicle (DMSO) only. After 1 h, LPS was added [except for the negative control (basal)] and NF-κB activity was evaluated 24 h later. Bars in the graph show mean ± SEM (standard error of mean). *** indicates statistical significance to DMSO only treated cells (*p* < 0.001); **** indicates statistical significance to DMSO only treated cells (*p* < 0.0001).

Sulfonamides are originally known as antibiotics, but many also exhibit significant anti-inflammatory properties. Classic examples include sulfasalazine, used in inflammatory bowel disease and rheumatoid arthritis. The anti-inflammatory actions of sulfonamides have been attributed to their ability to modulate key signaling pathways in immune cells. A unifying mechanism of anti-inflammatory sulfonamides is the suppression of NF-κB signaling at various levels. One well-documented example is sulfasalazine, which directly inhibits the IκB kinase complex [62]. Hence, the anti-inflammatory potential of the tested sulfonates was evaluated as their ability to influence the activity of pro-inflammatory transcription factor NF-κB in LPS-challenged cells (Figure 4). THP1 cells could be used as a simplified microglia-like model; hence, they could serve to study neuroinflammation, which is often connected with AD [63]. Apart from compounds **3** and **5c**, all other molecules significantly attenuated the activity of NF-κB. The most active was compound **5d** with the most bulky -CF_3_ substituent on the phenyl ring, which attenuated the NF-κB activity by 46% at 5 µM. Moreover, compounds **5e** and **5f** were able to decrease the activity of this transcription factor by more than 30% at a concentration of 5 µM. All these compounds (**5d**–**f**) bear an electronegative halogenated substituent on the phenyl ring. However, **5g** with a strong electronegative -CN group on the phenyl ring inhibited the activity of NF-κB only by 27%. Previous studies demonstrated the potential of sulfonamides to interfere with the NF-κB signaling pathway in different models (e.g., reference [64,65,66]).

### 2.4. ADME Properties and Drug-Likeness Evaluation

An ideal cholinesterase inhibitor should combine effective enzyme inhibition, low cytotoxicity to mammalian cells, and optimal physicochemical properties for drug-likeness and crossing the blood–brain barrier (BBB). The compounds with high inhibitory activity (**3**, **5a**, **5b**, **5c**, **5e**, **5f**, **5i**) and RIV (standard compound) were evaluated for their physicochemical properties and whether they satisfy Lipinski’s rule of five. Table 2 summarizes molecular weights, log *p* values, number of H-donors, number of H-acceptors and tPSA values. In addition, the gastrointestinal absorption and bioavailability scores are also presented.

It is evident from Table 2 that compounds **3**, **5a**, **5b**, **5c**, and **5i,** along with RIV, satisfy Lipinski’s rule 5 with no violation and demonstrate a high oral bioavailability score of 0.55. Unfortunately, when we include the tPSA value in the evaluation, only compound **3** demonstrates suitable properties for a substance intended as a CNS-targeting drug.

**Figure 5:**
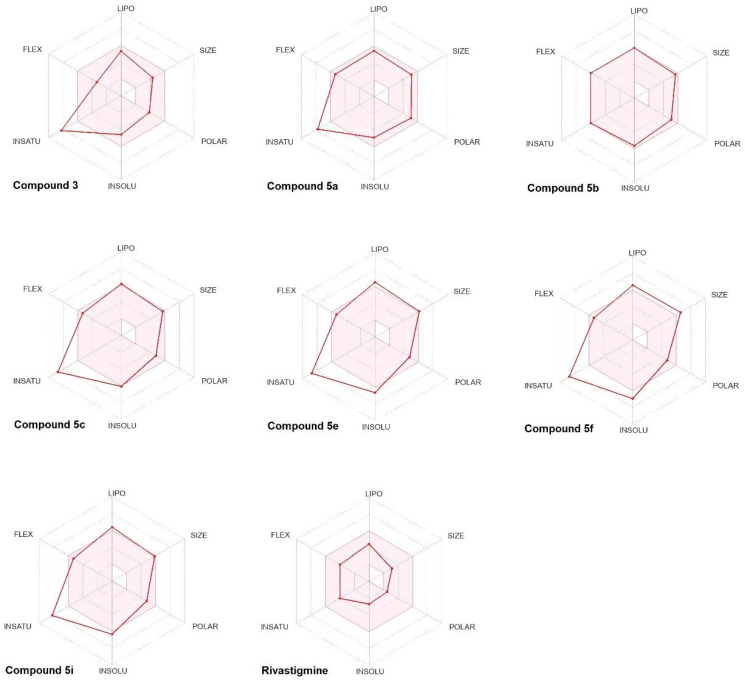
Bioavailability radar plots of compounds **3**, **5a**–**c**, **5e**, **5f**, **5i** and rivastigmine.

Bioavailability radar is displayed for quick assessment of drug-likeness (Figure 5). The radar representation illustrates six key physicochemical parameters influencing oral bioavailability, including lipophilicity, size, polarity, solubility, saturation, and flexibility. Only compound **5b** demonstrated a favorable profile within the optimal physicochemical space, but compared to RIV, the drug-likeness features are not as pronounced.

**Figure 6:**
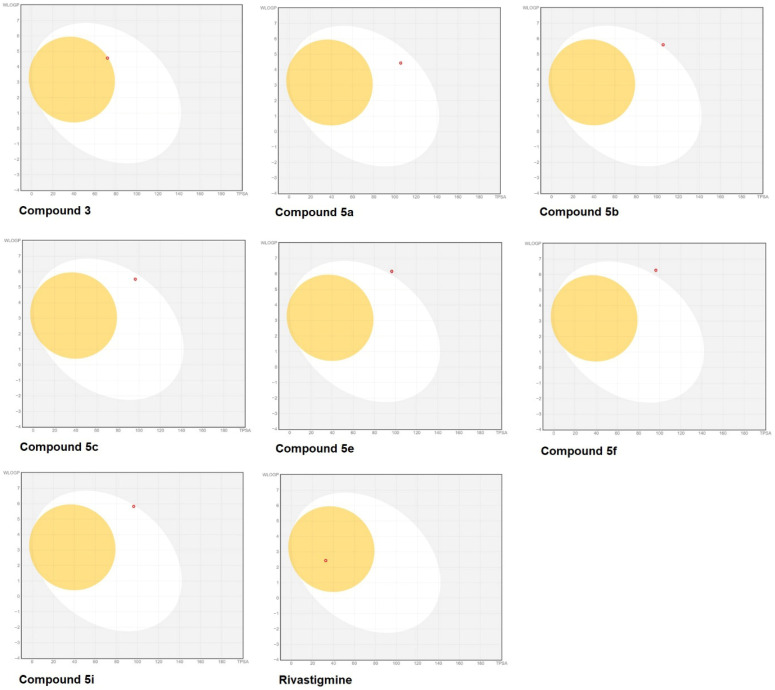
The BOILED-Egg plots of compounds **3**, **5a**–**c**, **5e**, **5f**, **5i** and rivastigmine.

The BOILED-Egg model [67] is an intuitive graphical classification model that includes predictions of two key ADME parameters, i.e., passive absorption in the human gastrointestinal tract (GIT) and permeation through the BBB (Figure 6). This classification model relies on only two physicochemical descriptors (WLOGP and tPSA for lipophilicity and apparent polarity). The yolk represents a physicochemical space for highly probable BBB permeation, and the white represents a physicochemical space for highly probable passive absorption in the human GIT.

As can be seen from Figure 6, only for the lead and the most active compound **3**, both passive absorption in the GIT and passive permeation through the BBB are predicted (in the yolk covering the area of the white). Unfortunately, this compound showed significant toxicity, see Figure 3. The selectivity index (ratio between toxicity (5.8 μM) and activity (0.74 μM)) is 7.8, which means that the molecule is interesting but has an inappropriate toxicity profile, and therefore, subsequent optimizations were performed. However, as can be seen from Table 1 and Table 2 and Figure 3 and Figure 6, removal of the aldehyde group from compound **3** resulted in an improvement in the toxicity profile, but on the other hand, it worsened the predicted bioavailability and the observed inhibitory activity. Compounds **5b**, **5e** and **5f** are expected to be neither passively absorbed in the GIT nor passively cross the BBB. Compounds **5a**, **5c** and **5i** are expected to be well absorbed in the GIT but not to reach the brain (in the white, see Figure 6).

However, there are various strategies to increase bioavailability both from the gastrointestinal tract and across the BBB into the brain. For example, the low bioavailability of GAL has been addressed by preparing a more lipophilic prodrug, memogain [68]. In the case of the discussed sulfonate-based cholinesterase inhibitors, there is no possibility to prepare derivatives with increased lipophilicity, because, as can be seen from Table 1, these compounds have significantly lower inhibitory activity against AChE/BChE (compounds **5d**–**f**). The preparation of classical prodrugs (ester/amide type) is also excluded, because the discussed compounds do not have a suitable free functional group [69] but the bioavailability can be increased by galenic modifications, especially by encapsulation into surface-modified lipid nanovesicles or fullerenes [70,71,72] or nanoemulsions and polymer nanosystems [73,74]. Furthermore, surface-modified formulations with so-called Trojan horses or various molecular fragments recognized by influx transporters can be applied [75,76].

### 2.5. Molecular Modeling and SAR

Molecular modeling was conducted using a tiered computational workflow that integrates methods with different levels of accuracy and computational cost. Specifically, we performed molecular docking, MD simulations coupled to residue-level interaction analyses, and QTAIM calculations. The results are presented in the subsequent section for two reasons: (i) to preserve the chronological order in which the computations were carried out and (ii) to streamline the presentation and discussion of these results.

In search of compounds that resemble the previously reported sulfonamides derivatives as selective BChE inhibitors [1], compound **3** was selected as a promising starting scaffold (Figure 1). Note that this compound is a suitable starting structure for the design of new derivatives sharing this structural skeleton. It is interesting to note that in this compound, we propose an isosteric replacement by substituting the NH group present in previously reported compounds [37] with an oxygen atom. In addition, compound **3** features a bicyclic moiety instead of the benzene ring found in the previously reported sulfonamide derivatives.

**Figure 7:**
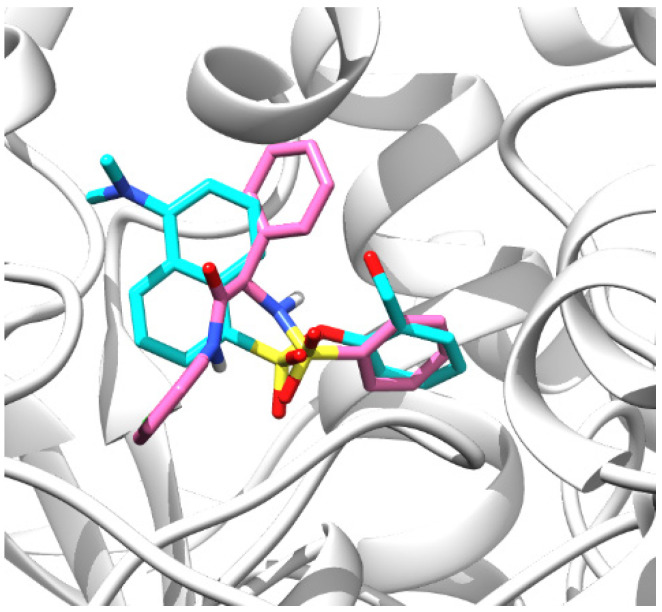
Spatial view of BChE-**3** (cyan) and BChE-A (bright pink) overimposed for comparison in the active site.

As a preliminary and exploratory study, docking calculations were performed to compare the binding molecular behaviors of **3** with that of compound **A** [1] (Figure 1). As shown in Figure 7, both compounds are positioned in the same region of the active site, near the CAS, with similar orientations, pointing sulfoxide oxygen toward the same location of the active site.

**Figure 8:**
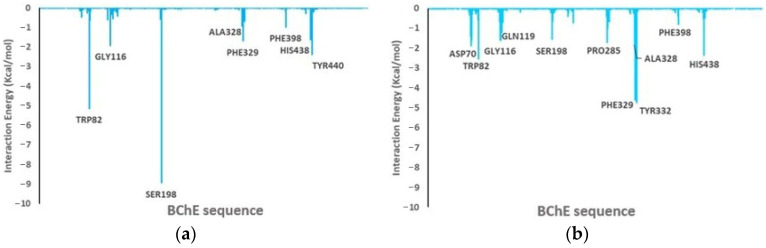
Histogram of interaction energies partitioned with respect to BChE amino acid sequence when complexed with compound **3** (**a**) and DON (**b**). The X-axis denotes the residue number of BChE, and the Y-axis denotes the interaction energy between the compound and a specific residue.

Although docking provides valuable preliminary insights, it requires further validation by more rigorous computational and experimental approaches. Thus, MD simulations were carried out to examine in more detail the binding of compound **3** in the BChE active site. A per-residue analysis to determine which amino acids mainly bind this ligand was also performed. The results were compared with those obtained for DON [24]. The per-residue analysis indicates that **3** might bind to BChE in a quite similar manner to that of DON, however compound **3** shows fewer interactions in comparison to DON. In particular, interactions with Asp70, Gln119, Pro285, and Tyr332 are missing in **3**. Conversely, **3** displayed stronger interactions with Trp82 and Ser198 (Figure 8). Figure 8 shows this situation quite well.

**Figure 9:**
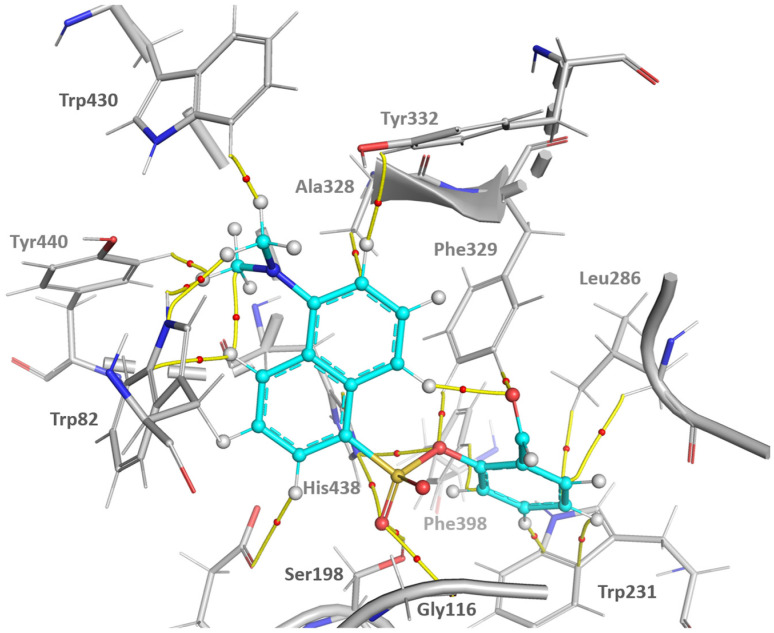
Charge density molecular graph of compound **3** (cyan) at the binding site of BChE. Residues from the active site are shown in gray. Topological elements of charge density associated with intermolecular interactions are depicted with yellow lines (bond paths) and small red spheres (bond critical points).

In addition to these key interactions in the BChE-**3** complex, further stabilizing interactions were observed with Gly116, Phe329, His438, and Tyr440. Next, in order to quantitatively evaluate these interactions, a QTAIM analysis was then performed. The charge density molecular graph of compound **3** complexed with BChE (Figure 9) shows that this ligand reaches the bottom of the gorge, thus interacting with residues within the active site of BChE.

Compound **3** adopts a stable binding pose within the active site, where it establishes multiple interactions that ensure effective anchorage to the binding pocket. The benzaldehyde moiety is positioned at the bottom of the cavity and primarily establishes hydrophobic interactions with surrounding residues, including Gly116, Gly117, Trp231, Leu286, Phe329, Phe398, and His438. Particularly, the sulfonate group forms a hydrogen bond with the hydroxyl group of Ser198. A second anchoring region is provided by the 1-(*N*,*N*-dimethylamino)naphthalene moiety, which is oriented toward the entrance of the pocket. This aromatic system is surrounded by residues such as Ala328, His438, and Trp430, while the methyl groups of the dimethylamino substituent are stabilized through hydrophobic interactions with Trp80, Trp428, and Tyr438. Altogether, these interactions contribute to the stabilization of the enzyme–ligand complex (Figure 9).

The results obtained from our molecular simulations suggested that compound **3** could represent a promising candidate as a BChE inhibitor. Consequently, this compound was synthesized and experimentally evaluated as a possible inhibitor of this enzyme. The details of the synthesis are shown in Section 2.1 Chemistry, in the Results section, and its biological activity is reported in Table 1. The inhibitory effect of this compound is strong, showing quite a specific activity as a BChE inhibitor (IC_50_ values: 0.74 μM for BChE vs. 52.2 μM for AChE). Simultaneously, compound **3** also showed significant anti-inflammatory activity (Figure 4). Although these results were highly encouraging, it was considered appropriate to assess the potential toxicity of this compound. As a first attempt, we carried out an in silico toxicity prediction using ProTox 3.0 [77], which classified compound **3** as acute oral toxicity class 5 with an LD_50_ value of 4000 mg/kg (prediction accuracy of 70%). Furthermore, the drug was predicted to be active for BBB permeability, immunotoxicity and clinical toxicity according to class toxicology endpoints. It is interesting to note that the aldehyde group in compound **3** was identified as a possible factor responsible for its toxicity. These preliminary in silico results were then experimentally corroborated, potentially limiting the therapeutic applicability of this ligand (Figure 3).

**Figure 10:**
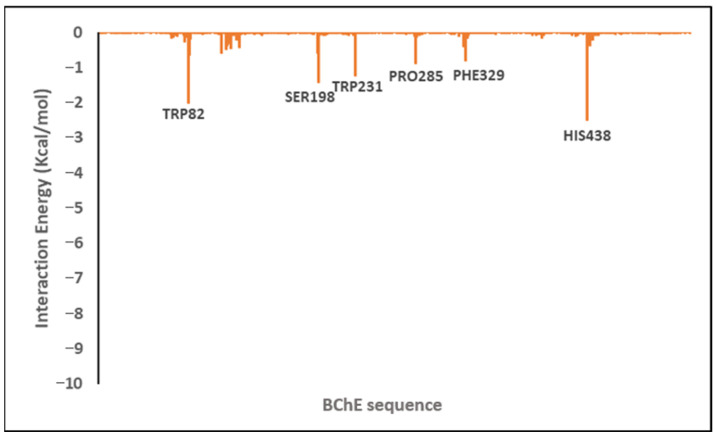
Histogram of interaction energies partitioned with respect to the BChE amino acid sequence when complexed with compound **5a**. The X-axis denotes the residue number of BChE, and the Y-axis denotes the interaction energy between the compound and a specific residue.

Based on these findings, structural modifications of **3** were pursued in order to reduce its toxicity by incorporating bulkier groups that could reduce the high reactivity of the aldehyde group while preserving the BChE inhibitory effect. It is worth noting that introducing moderately bulky groups would not cause steric constraints within the active site, as our simulations indicated sufficient space to accommodate substituents of this characteristic. Thus, the aldehyde group was replaced with a hydrazone moiety, yielding compound **5a**. Prior to synthesis, molecular simulations were performed to assess its binding behavior, see Figure 10.

Our simulations showed that compound **5a** could adopt a quite similar spatial arrangement to that of **3** and DON in the BChE active site. However, in this case, the interactions are weaker than those observed for **3**, suggesting a weaker inhibitory effect. In silico toxicity predictions for **5a** were encouraging, since they predict toxicity class 6 for this compound, with an LD_50_ value of 5000 mg/kg (prediction accuracy of 56.24%). Based on these results, we proceeded to synthesize **5a** and evaluate its inhibitory activity against BChE, along with its anti-inflammatory properties and potential toxicity. Experimental results were in complete agreement with our simulations. Compound **5a** retained selective inhibitory activity against BChE and anti-inflammatory activity while exhibiting no detectable cytotoxicity (Table 1, Figure 3 and Figure 4). However, compound **5a** possesses a significantly weaker inhibitory effect compared to **3** (IC_50_ = 27.69 vs. 0.74 μM), in agreement with computational predictions.

It is important to analyze the inhibitory potency of the most active compounds reported in this work in the context of known cholinesterase inhibitors. Notice that under the same experimental conditions, rivastigmine showed an IC_50_ value of 38 μM toward BChE (Table 1). Notably, five compounds (**3**, **5a**–**c**, and **5i**) from the present series exhibited higher inhibitory activity than this reference inhibitor. In previous studies from our group, DON and GAL displayed IC_50_ values of 4.7 μM and 11.4 μM, respectively, against the same enzyme [24] (slightly different from the values currently given in Table 1). The improved activity observed relative to rivastigmine and their selectivity toward BChE highlight this scaffold as a promising starting point for further optimization.

**Figure 11:**
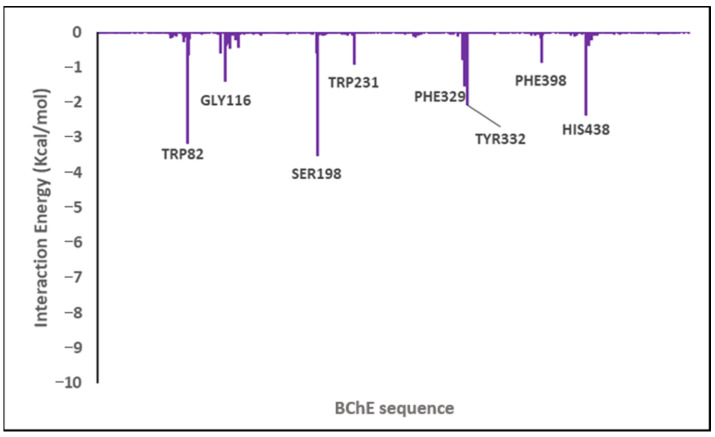
Histogram of interaction energies partitioned with respect to BChE amino acid sequence when complexed with compound **5b**. The X-axis denotes the residue number of BChE, and the Y-axis denotes the interaction energy between the compound and a specific residue.

To further enhance activity, the methyl substituent in compound **5a** was replaced by a *tert*-butyl group, yielding compound **5b**. The simulations obtained for compound **5b** were very encouraging as they showed stronger stabilizing interactions for the BChE-**5b** complex (Figure 11). Furthermore, QTAIM calculations predict total ∑ρ(r) values comparable to those of compound **3** (0.3361 vs. 0.3297, respectively).

QTAIM calculations performed for **5b** yielded very interesting results (see Figure S1 in the Supporting Materials). The bulkier derivative **5b** adopts a spatial arrangement similar to that of the previously discussed compounds, although it shows a slight deviation from the more active compound **3**. As a consequence of its larger size, the ligand establishes increased interactions with residues near the surface of the catalytic gorge. This results in a slightly weaker hydrogen bond between the sulfonate group of **5b** and the hydroxyl group of Ser198. The corresponding electron density values for this interaction are 0.0310 for **3** and 0.0206 for **5b**, consistent with the average donor–acceptor distances obtained from MD simulations (2.0 Å for **3** and 2.3 Å for **5b**).

Regarding the 1-naphthylamino moiety, the interactions observed in the BChE-**5b** complex are weaker than those found for **3**. Particularly, some interactions of the naphthyl moiety, such as those with Ala328, Tyr332 and Tyr440, present in the BChE-**5a** complex are missing in **5b**. In contrast, the substituted hydrazone moiety of **5b** establishes stronger interactions, which appear to compensate for this effect. In particular, the *tert*-butyl substituent is located towards the surface of the gorge and interacts with residues of the peripheral anionic site, including Tyr332, which may contribute to the anticholinesterase activity observed for this compound.

Based on these results, we decided to synthesize **5b**, and bioassays for this compound were subsequently carried out. Consistent with these predictions, compound **5b** displayed more than a two-fold improvement in BChE inhibitory activity compared to **5a**, showing no cytotoxic effects and maintaining anti-inflammatory activity. These results were in line with our simulations. Nevertheless, the inhibitory effect of **5b** remained lower than that of compound **3**.

**Figure 12:**
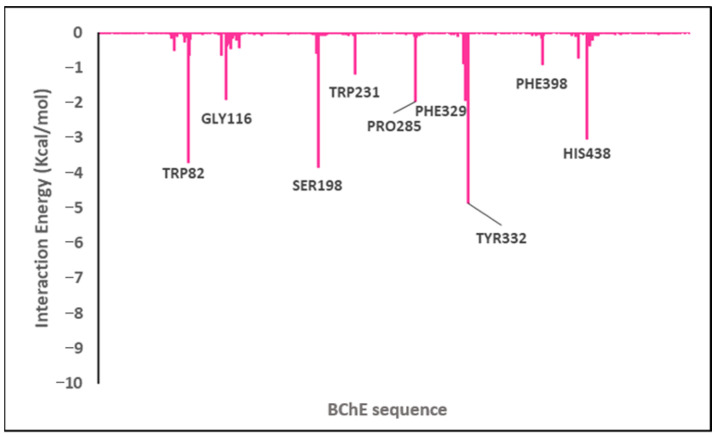
Histogram of interaction energies partitioned with respect to BChE amino acid sequence when complexed with compound **5c**. The X-axis denotes the residue number of BChE, and the Y-axis denotes the interaction energy between the compound and a specific residue.

Considering the remaining space available within the binding pocket, the *tert*-butoxy group of **5b** was replaced by a phenyl substituent, giving compound **5c**. The simulation results for this compound were promising, as the residue analysis of this compound closely resembled that of compound **3** (compare Figure 8a and Figure 12). QTAIM analysis yielded total ∑ρ(r) values comparable to those of compounds **3** and **5b** (0.3297 for **3**, 0.3361 for **5b**, and 0.3192 for **5c**).

**Figure 13:**
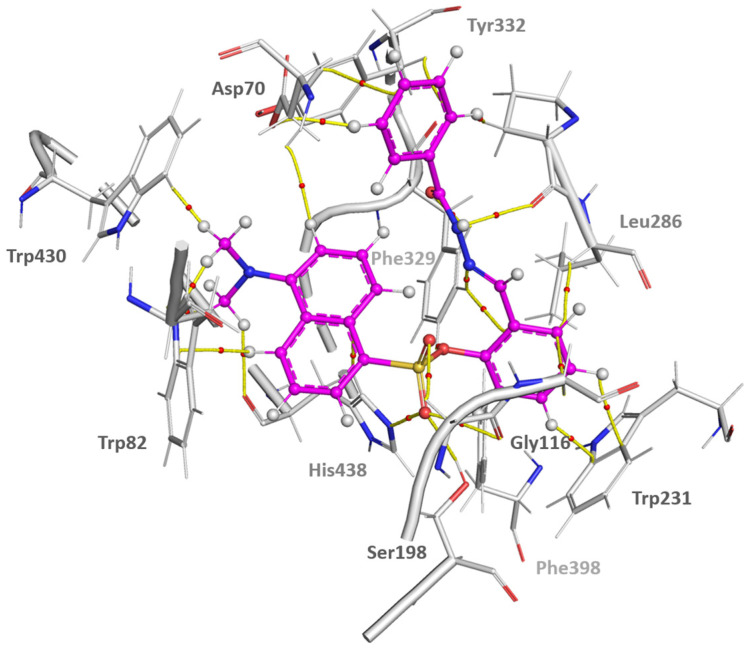
Charge density molecular graph of compound **5c** (magenta) at the binding site of BChE. Residues from the active site are shown in gray. Topological elements of charge density associated with intermolecular interactions are depicted with yellow lines (bond paths) and small red spheres (bond critical points).

**Figure 14:**
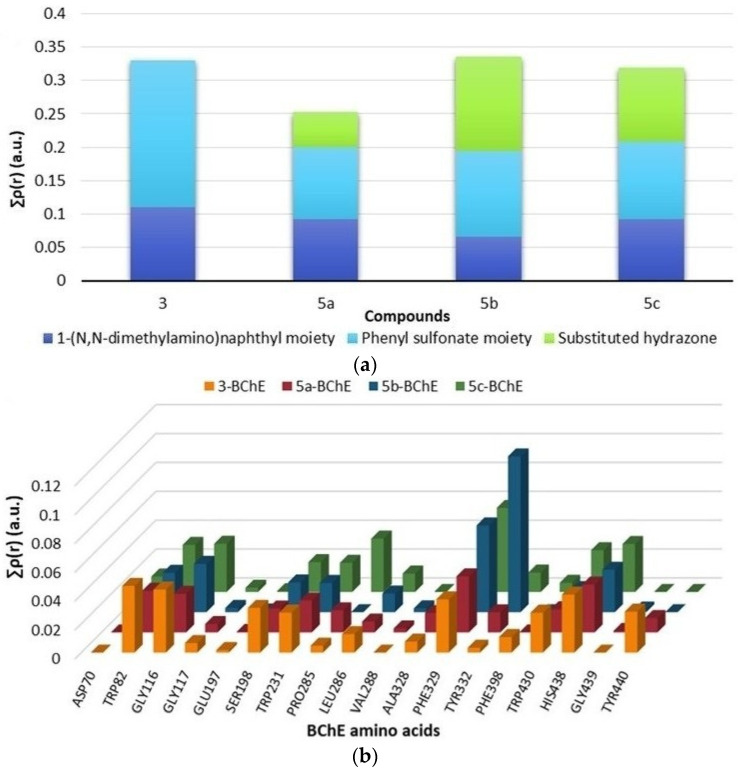
Sum of charge density values (atomic units) at the critical intermolecular binding points obtained for the different complexes are shown as bar charts that represent: (**a**) contributions from the three ligand structural moieties, and (**b**) detailed interaction densities with individual amino acids.

Figure 13 shows the charge density molecular graph of compound **5c** at the binding site of BChE. These results are comparable to those obtained for compounds **3** and **5b**. As observed for **5b**, interactions involving the 1-naphthyl moiety of **5c** were weaker than in compound **3**. However, these were compensated by strong interactions involving the substituted hydrazone region, including residues Phe239 and Tyr332 (Figure 13 and Figure 14).

Based on our simulations, we synthesized and then tested compound **5c** (see Section 2.1 and Section 2.2). Experimental evaluation confirmed these predictions: compound **5c** exhibited potent and selective BChE inhibition (IC_50_ = 1.1 μM for BChE vs. 63.7 μM for AChE), with no detectable cytotoxicity and preserved anti-inflammatory activity, identifying it as a promising hit compound.

Further extension of the terminal chain of the hydrazone moiety led to compounds **5d**–**j** (Table 1). Unfortunately, none of these derivatives with more extended end chains surpassed the activity of compounds **3**, **5c** and **5b**. In fact, some of them exhibited marginal BChE inhibitory activity and, in some cases, compounds showed greater inhibition of AChE than BChE. Nevertheless, none of these compounds showed promising cholinesterase inhibitory effects. To better understand these experimental results, molecular simulations were performed. These simulations revealed that compounds **5d**–**j** adopted binding poses distinct from those of the most active derivatives, with per-residue interaction patterns that were not comparable to those observed for compounds **3**, **5c**, and **5b**.

Figure S2 in the Supporting Materials shows the different behavior observed for compound **5d**. Similar results were obtained for the rest of these inactive compounds. In particular, our simulations indicate that *para*-substitution on the terminal phenyl ring introduced steric constraints within the binding site. Thus, they are unable to adopt spatial arrangements comparable to those of DON, **3**, **5b** and **5c**. In contrast, compound **5d** adopts a folded conformation corresponding to a markedly different spatial arrangement. This geometry results in the loss of interaction with the catalytic Ser residue, as it favors the formation of intramolecular contacts. As we previously reported, such conformations promote intramolecular stabilization at the expense of productive stabilizing intermolecular interactions with residues within the enzyme active site, which are essential for complex formation.

An interesting result to highlight is that **5d**–**j** displayed no detectable cytotoxicity and retained anti-inflammatory activity. The lack of toxicity in these compounds further supports our strategy of replacing the aldehyde group as a means to reduce toxicity while preserving biological activity.

It is important to emphasize that QTAIM analysis proved particularly valuable for quantitatively evaluating enzyme–ligand interactions across the series. In fact, this study, combined with the per-residue interaction energy analysis obtained from the MD calculations, has provided the information that allowed for the synthesis of these new compounds. Figure 14 summarizes the total ∑ρ(r) values obtained for the most active compounds in the series and the contribution of the different three ligand regions: 1-(*N*,*N*-dimethylamino)naphthyl moiety, phenyl sulfonate moiety, and substituted hydrazone. The dissection of these three moieties allowed a detailed assessment of the structural determinants of binding, assessing which portion established the main interactions that stabilized their respective complexes.

From Figure 14, the most active compounds displayed comparable total ∑ρ(r) values to the 1-naphthylamine moiety (blue bar in Figure 14a), which is consistent since it is a conserved structure throughout the series. Compound **5b** showed slightly reduced values for this region, attributable to the fact that this compound establishes very strong interactions with the substituted hydrazone portion, at the other extreme of the molecule (green bar in Figure 14a), which required partial “stretching” of the ligand to accommodate both interaction domains. Notably, compound **3** displayed the strongest interactions involving the sulfonate region. This behavior is related to the sulfonate group forming a strong hydrogen bond with Ser198, as well as effective interactions between the benzene ring and aromatic residues, such as Trp231, Phe329, and Phe398. It should be noted that the three most active compounds **3**, **5b** and **5c** show significantly higher ∑ρ(r) values than **5a**, which is in full agreement with the inhibitory activity values observed for these compounds.

Although the total ∑ρ(r) value of compound **5b** was slightly overestimated regarding its lower inhibitory effect compared with compound **5c**, such discrepancies are expected given the sensitivity of QTAIM calculations to geometric parameters. Thus, small changes in the selected geometry can lead to this type of error. In fact, this type of QTAIM calculations should not be interpreted as providing absolute quantitative predictions but rather as a very useful tool to differentiate between highly active, moderately active (**5a**), and inactive compounds. The combined MD and QTAIM analyses demonstrated strong predictive capacity for this compound series and effectively guided structure-based optimization.

**Figure 15:**
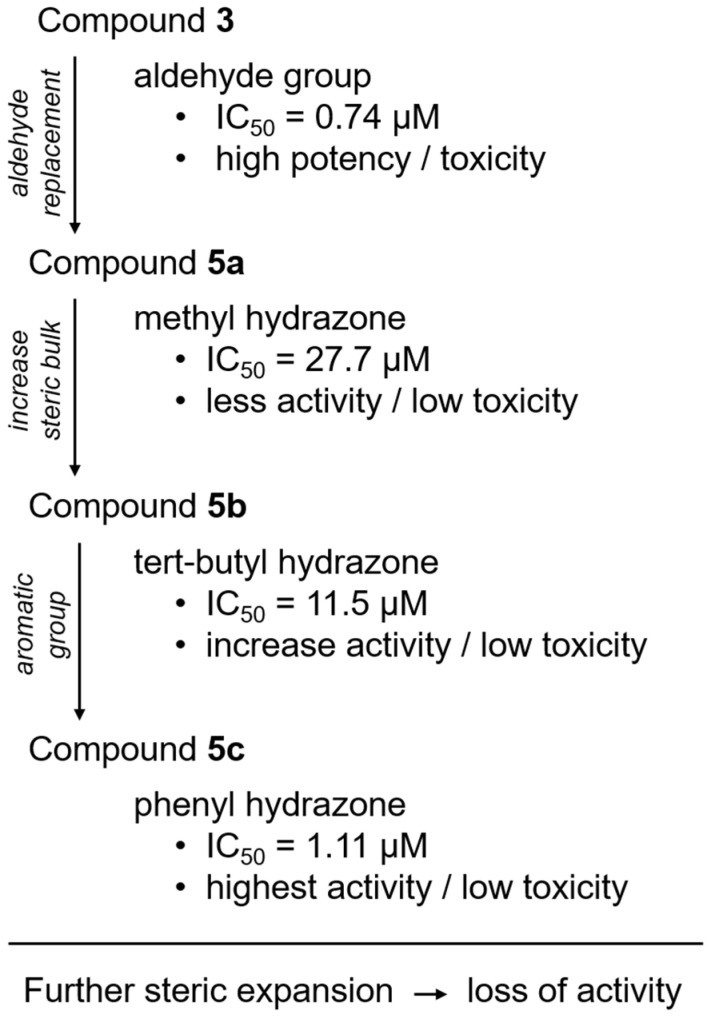
Summary of the structure–activity relationships (SARs) observed for the sulfonate derivatives evaluated as BChE inhibitors.

Finally, several observations and generalizations of the overall observed structure-activity relationships (SARs) in this series highlight several relevant structural features that govern BChE inhibition. The presence of the aldehyde group in compound **3** enables strong interactions within the catalytic gorge, particularly through the formation of a hydrogen bond between the sulfonate group and Ser198, resulting in high inhibitory potency. However, this functional group is also associated with increased toxicity. Replacing the aldehyde with hydrazone derivatives successfully reduced toxicity but initially decreased inhibitory activity, as observed for compound **5a**. Increasing the steric bulk of the terminal substituent partially restored inhibitory potency in compounds **5b** and **5c**, likely due to increased hydrophobic interactions with residues located near the entrance of the catalytic gorge, including Tyr332. In contrast, further extension of the terminal substituent (compounds **5d**–**j**) introduced steric constraints that prevented the ligands from adopting productive binding orientations within the active site. These findings suggest that the BChE gorge can accommodate moderately bulky substituents while still imposing spatial limitations that strongly influence inhibitory activity. The observed SAR trends are supported by molecular dynamics and QTAIM analyses, which indicate stronger interaction densities for the most active compounds of the series. A summary of the SAR observed for this series is presented in Figure 15.

## 3. Materials and Methods

### 3.1. Chemistry

All reagents and solvents were purchased from commercial sources (Sigma-Aldrich (Czech Republic, Prague), Fluorochem (Penrose Dock, Cork T23 YY09 Ireland), Acros Organics (Geel, Belgium), TCI Europe (Haven, Belgium), Merck (Prague, Czech Republic), and Lach-Ner (Neratovice, Czech Republic)). Commercial-grade reagents were used without further purification. Reactions were monitored by using thin-layer chromatography (TLC) plates coated with 0.2 mm silica gel (60 F254, Merck). TLC plates were visualized by using UV irradiation (254 nm). All melting points were determined by using a Melting Point B-540 apparatus (Büchi, Switzerland) and are given in their uncorrected form. The IR spectra were recorded with a Nicolet 6700 FTIR spectrometer (Thermo Fisher Scientific, Waltham, MA, USA) over the range 400–4000 cm^−1^ by using the ATR technique. The NMR spectra were measured in CDCl_3_ and CD_2_Cl_2_-d_2_ solutions at ambient temperature with a Bruker AvanceTM III 400 spectrometer (Brno, Czech Republic) at frequencies 400 MHz (^1^H), 470 MHz (^19^F) and 100.26 MHz (^13^C), or with a Bruker Ascend™ 500 spectrometer (Brno, Czech Republic)at frequencies 500.13 MHz (1H) and 125.76 MHz (^13^C{^1^H}). The chemical shifts (d) reported in the Supporting Information are given in ppm and are related to the following residual solvent peaks: ≅7.27 (CDCl_3_), ≅2.50 (DMSO-d_6_). Tetramethylsilane (TMS) was used as an internal standard. The coupling constants (*J*) are reported in Hz. Elemental analyses (C, H, and N) were performed with an automatic microanalyzer (Flash 2000 Organic elemental analyser) (Thermo Fisher Scientific, Waltham, MA, USA). Mass spectrometry with high resolution was determined by the “dried droplet” method using a MALDI mass spectrometer LTQ Orbitrap XL (Thermo Fisher Scientific) equipped with a nitrogen UV laser (337 nm, 60 Hz). Spectra were measured in positive ion mode and in regular mass extent with a resolution of 100,000 at a mass-to-charge ratio (*m*/*z*) of 400, with 2,5-dihydrobenzoic acid (DBH) used as the matrix.

#### Synthesis and Characterization of Investigated Molecules **5a**–**j** and Key Intermediate **3**

*Synthetic procedure and characterization for the preparation of 2-formylphenyl 5-(dimethylamino)naphthalene-1-sulfonate* (**3**): To a stirring solution of salicylaldehyde (**1**, 1.0 g, 1.0 equiv.) and 5-(dimethylamino)naphthalene-1-sulfonyl chloride (**2**, 2.64 g, 1.2 equiv.) in DCM, triethylamine (1.40 mL, 1.2 equiv.) was slowly added at 0 °C. Then the reaction mixture was stirred at room temperature and the reaction was monitored by TLC (50% EtOAc:Hexane). After completion of the reaction (20 h), the reaction mixture was extracted with water, and the organic layer was separated and dried over sodium sulfate. The solvent was removed under reduced pressure and the obtained crude product was purified by column chromatography (*n*-hexane-EtOAc, 25%). Yield: 60.6%, yellow solid; Rf: 0.58 (*n*-hexane/EtOAc = 50%), m.p.: 66.1–66.4 °C. IR (ATR, cm^−1^): 2985, 2944, 2827, 1691, 1599, 1567, 1475, 1406, 1392, 1369, 1204, 1185, 1163, 1147, 1087, 863, 789, 703, 572. ^1^H NMR (500 MHz, CDCl_3_): *δ* 10.18 (s, 1H, CHO), 8.67 (d, *J* = 8.5 Hz, 1H, aromatic), 8.48 (d, *J* = 8.6 Hz, 1H, aromatic), 8.11 (d, *J* = 7.2 Hz, 1H, aromatic), 7.87 (dd, *J* = 7.7, 1.5 Hz, 1H, aromatic), 7.69 (t, *J* = 8.1 Hz, 1H, aromatic), 7.49 (t, *J* = 7.9 Hz, 1H, aromatic), 7.46–7.41 (m, 1H, aromatic), 7.33 (t, *J* = 7.5 Hz, 1H, aromatic), 7.28 (d, *J* = 7.7 Hz, 1H, aromatic), 6.89 (d, *J* = 8.2 Hz, 1H, aromatic), 2.93 (s, 6H, N(CH_3_)_2_), Figure S3. ^13^C NMR (126 MHz, CDCl_3_): *δ* 188.45, 152.15, 135.96, 133.47, 132.14, 130.94, 130.67, 130.52, 130.23, 130.14, 129.32, 128.17, 123.75, 123.67, 119.61, 116.63, 46.15, Figure S4. MALDI-TOF: [M+H]^+^ Calcd. for C_19_H_18_NO_4_S 356.09511; found 356.09146, Figure S5. Elemental analysis: Calc. for C_19_H_17_NO_4_S: C, 64.21; H, 4.82; N, 3.94; found: C, 64.32 ± 0.08; H, 4.81 ± 0.04; N, 3.88 ± 0.01.

*General procedure for the synthesis of (E)-2-[(2-substituted hydrazono)methyl]phenyl 5-(dimethylamino)naphthalene-1-sulfonates* (**5a**–**j**): Chosen hydrazide (**4a**–**j**, 1.25 mmol, 1.5 equiv.) was added to a stirring solution of aldehyde (**3**, 0.84mml, 1.0 equiv.) in CH_3_CN (50 ML) at room temperature. Acetic acid (4 drops) was added to the reaction mixture at room temperature. The reaction was monitored by TLC (60% EtOAc:Hexane). After completion of the reaction (typically 20 h), the solvent was removed under reduced pressure. The crude product was purified by column chromatography (*n*-hexane-EtOAc, 30%), and compounds 5a–j were obtained as a yellow solid in excellent yields.

*Methyl (2E)-2-[(2-{[5-(dimethylamino)naphthalene-1-sulfonyl]oxy}phenyl)methylidene]hydrazine-1-carboxylate* (**5a**)**:** Yield: 90.3%, yellow solid; Rf: 0.73 (*n*-hexane/EtOAc = 60%), m.p.: 82.3–82.9 °C. IR (ATR, cm^−1^): 3227, 2947, 1715, 1604, 1569, 1538, 1479, 1448, 1366, 1285, 1233, 1203, 1185, 1147, 1085, 1051, 945, 876, 836, 778, 769, 566. ^1^H NMR (500 MHz, CDCl_3_): *δ* 11.28 (s, 1H, NH), 8.62 (s, 1H, CH), 8.28 (d, *J* = 8.6 Hz, 2H, aromatic), 8.14 (d, J = 7.2 Hz, 1H, aromatic), 7.82 (d, *J* = 7.3 Hz, 1H, aromatic), 7.75 (t, *J* = 8.1 Hz, 1H, aromatic), 7.65 (t, *J* = 7.9 Hz, 1H, aromatic), 7.31 (dd, *J* = 15.2, 7.6 Hz, 2H, aromatic), 7.25 (dd, *J* = 10.8, 4.7 Hz, 1H, aromatic), 6.65 (d, *J* = 7.9 Hz, 1H, aromatic), 3.70 (s, 3H, OCH3), 2.85 (s, 6H, N(CH_3_)_2_), Figure S6. ^13^C NMR (126 MHz, CDCl_3_): 152.98, 148.40, 133.71, 132.70, 131.87, 130.88, 130.77, 130.31, 130.11, 129.21, 128.82, 127.51, 124.78, 122.78, 119.23, 116.88, 53.18, 46.19, Figure S7. MALDI-TOF: [M+H]^+^ Calcd. for C_21_H_22_N_3_O_5_S 428.12747; found 428.12265, Figure S8. Elemental analysis: Calc. for C_21_H_21_N_3_O_5_S: C, 59.00; H, 4.95; N, 9.83; found: C, 58.60 ± 0.25; H, 4.79 ± 0.03; N, 9.18 ± 0.02.

*tert-Butyl (2E)-2-[(2-{[5-(dimethylamino)naphthalene-1-sulfonyl]oxy}phenyl)methylidene]hydrazine-1-carboxylate* (**5b**): Yield: 91.6%, yellow solid; Rf: 0.68 (*n*-hexane/EtOAc = 60%), m.p.: 81.3–81.8 °C. IR (ATR, cm^−1^): 3231, 2977, 2788, 1714, 1604, 1569, 1478, 1409, 1392, 1235, 1185, 1145, 1084, 884, 788, 518. ^1^H NMR (500 MHz, CDCl_3_): *δ* 11.09 (s, 1H, NH), 8.63 (d, *J* = 8.5 Hz, 1H, aromatic), 8.33–8.23 (m, 2H, aromatic), 8.15 (d, *J* = 7.2 Hz, 1H, aromatic), 7.82 (d, *J* = 7.6 Hz, 1H, aromatic), 7.75 (t, *J* = 8.1 Hz, 1H, aromatic), 7.66 (t, *J* = 7.9 Hz, 1H, aromatic), 7.31 (dd, *J* = 19.2, 7.6 Hz, 2H, aromatic), 7.23 (t, *J* = 7.7 Hz, 1H, aromatic), 6.62 (d, *J* = 8.2 Hz, 1H, aromatic), 2.86 (s, 6H, N(CH_3_)_2_), 1.48 (s, 9H, 3CH_3_), Figure S9. ^13^C NMR (125 MHz, CDCl_3_): *δ* 152.27, 147.60, 132.94, 131.90, 130.92, 130.37, 130.01, 129.62, 129.43, 128.82, 128.06, 126.67, 124.09, 122.01, 118.60, 116.20, 45.51, 28.52, Figure S10. MALDI-TOF: [M+Na]^+^ Calcd. for C_24_H_27_N_3_O_5_SNa 492.15636; found 492.15720, Figure S11. Elemental analysis: Calc. for C_21_H_21_N_3_O_5_S: C, 61.39; H, 5.80; N, 8.95; found: C, 60.54 ± 0.005; H, 5.72 ± 0.03; N, 8.11 ± 0.02.

*2-[(E)-(2-Benzoylhydrazinylidene)methyl]phenyl 5-(dimethylamino)naphthalene-1-sulfonate* (**5c**): Yield: 92.2%, yellow solid; Rf: 0.72 (*n*-hexane/EtOAc = 60%), m.p.: 209.9–210.8 °C. IR (ATR, cm^−1^): 3235, 3062, 2830, 1637, 1605, 1539, 1397, 1280, 1185, 1093, 1075, 911, 865, 569. ^1^H NMR (500 MHz, DMSO-d_6_): *δ* 11.99 (s, 1H, NH), 8.72 (s, 1H, CH), 8.61 (d, *J* = 8.5 Hz, 1H, aromatic), 8.33 (d, *J* = 8.5 Hz, 1H, aromatic), 8.15 (d, *J* = 7.2 Hz, 1H, aromatic), 7.95 (d, *J* = 7.2 Hz, 3H, aromatic), 7.76 (t, *J* = 8.1 Hz, 1H, aromatic), 7.65 (t, *J* = 8.0 Hz, 2H, aromatic), 7.58 (t, *J* = 7.2 Hz, 2H, aromatic), 7.35 (t, *J* = 7.3 Hz, 1H, aromatic), 7.29 (dd, *J* = 15.4, 7.7 Hz, 2H, aromatic), 6.66 (d, *J* = 8.0 Hz, 1H, aromatic), 2.81 (s, 6H, N(CH_3_)_2_), Figure S12. ^13^C NMR (126 MHz, DMSO-d_6_): *δ* 164.18, 152.97, 148.91, 142.55, 134.26, 133.77, 133.14, 132.86, 132.38, 130.80, 130.77, 130.32, 130.10, 129.70, 129.14, 128.90, 128.88, 127.76, 124.78, 122.73, 119.24, 116.84, 45.14, Figure S13. MALDI-TOF: [M+Na]^+^ Calcd. for C_26_H_23_N_3_O_4_SNa 496.13015; found 496.13181, Figure S14. Elemental analysis: Calc. for C_26_H_23_N_3_O_4_S: C, 65.94; H, 4.90; N, 8.87; found: C, 65.20 ± 0.07; H, 4.88 ± 0.03; N, 8.35 ± 0.03.

*2-[(E)-{2-[4-(Trifluoromethyl)benzoyl]hydrazinylidene}methyl]phenyl 5-(dimethylamino)naphthalene-1-sulfonate* (**5d**): Yield: 87.1%, yellow solid; Rf: 0.76 (*n*-hexan/EtOAc = 80%), m.p.: 103.1–103.8 °C. IR (ATR, cm^−1^): 3223, 2835, 1654, 1607, 1568, 1480, 1451, 1323, 1288, 1203, 1185, 1163, 1147, 1125, 1060, 1016, 945, 857, 788, 753. ^1^H NMR (500 MHz, DMSO-d_6_): *δ* 12.18 (s, 1H, NH), 8.72 (s, 1H, CH), 8.62 (d, *J* = 8.5 Hz, 1H, aromatic), 8.31 (d, *J* = 8.7 Hz, 1H, aromatic), 8.14 (d, *J* = 7.4 Hz, 3H, aromatic), 8.02–7.92 (m, 3H, aromatic), 7.76 (t, *J* = 8.1 Hz, 1H, aromatic), 7.65 (t, *J* = 8.0 Hz, 1H, aromatic), 7.31–7.36 (m, 3H, aromatic), 6.65 (d, *J* = 8.1 Hz, 1H, aromatic), 2.81 (s, 6H, N(CH_3_)_2_), Figure S15. ^13^C NMR (126 MHz, DMSO-d_6_): *δ* 163.04, 152.98, 148.99, 143.41, 138.05, 133.81, 132.98, 132.89, 132.72, 132.63, 130.80, 130.74, 130.31, 130.10, 129.86, 128.93, 127.82, 126.74, 126.71, 126.68, 126.16, 124.79, 124.06, 122.77, 119.21, 116.85, 46.14, Figure S16. ^19^F NMR (470 MHz, DMSO-d_6_): δ −61.35, Figure S17. MALDI-TOF: [M+H]^+^ Calcd. for C_27_H_22_F_3_N_3_O_4_S 542.13559; found 542.13666, Figure S18. Elemental analysis: Calc. for C_27_H_22_F_3_N_3_O_4_S: C, 59.88; H, 4.09; N, 7.76; found: C, 59.79 ± 0.03; H, 4.12 ± 0.07; N, 7.34 ± 0.10.

*2-{(E)-[2-(4-Chlorobenzoyl)hydrazinylidene]methyl}phenyl 5-(dimethylamino)naphthalene-1-sulfonate* (**5e**): Yield: 88.3%, yellow solid; Rf: 0.73 (*n*-hexane/EtOAc = 60%), m.p.: 101.6–102.3 °C. IR (ATR, cm^−1^): 3229, 3143, 2832, 1651, 1592, 1568, 1478, 1450, 1369, 1294, 1269, 1185, 1203, 1162, 1146, 1090, 1060, 1014, 864, 788, 751, 574. ^1^H NMR (500 MHz, DMSO-d_6_): *δ* 12.04 (s, 1H, NH), 8.70 (s, 1H, CH), 8.62 (d, *J* = 8.5 Hz, 1H, aromatic), 8.31 (d, *J* = 8.6 Hz, 1H, aromatic), 8.14 (d, *J* = 7.3 Hz, 1H, aromatic), 7.95 (dd, *J* = 15.6, 7.9 Hz, 3H, aromatic), 7.76 (t, *J* = 8.1 Hz, 1H, aromatic), 7.72–7.61 (m, 3H, aromatic), 7.35 (dd, *J* = 14.0, 6.5 Hz, 1H, aromatic), 7.32–7.24 (m, 2H, aromatic), 6.65 (d, *J* = 8.1 Hz, 1H, aromatic), 2.82 (s, 6H, N(CH_3_)_2_), Figure S19. ^13^C NMR (126 MHz, DMSO-d_6_): *δ* 163.11, 152.98, 148.93, 142.88, 137.97, 133.80, 132.95, 132.88, 132.51, 130.84, 130.79, 130.74, 130.31, 130.09, 129.82, 129.03, 128.92, 127.78, 124.79, 122.75, 119.21, 116.86, 46.16, Figure S20. MALDI-TOF: [M+H]^+^ Calcd. for C_26_H_23_ClN_3_O_4_S 508.10923; found 508.10321, Figure S21. Elemental analysis: Calc. for C_26_H_22_ClN_3_O_4_S: C, 61.42; H, 4.37; N, 8.27; found: C, 61.10 ± 0.02; H, 4.36 ± 0.01; N, 7.83 ± 0.04.

*2-{(E)-[2-(4-Bromobenzoyl)hydrazinylidene]methyl}phenyl 5-(dimethylamino)naphthalene-1-sulfonate* (**5f**): Yield: 89.7%, yellow solid; Rf: 0.54 (*n*-hexane/EtOAc = 60%), m.p.: 103.7–104.3 °C. IR (ATR, cm^−1^): 3202, 2942, 2832, 2786, 1650, 1606, 1587, 1567, 1478, 1396, 1367, 1288, 1268, 1162, 1146, 1090, 1010, 945, 863, 747, 573. ^1^H NMR (500 MHz, DMSO-d_6_): *δ* 11.94 (s, NH, 1H), 8.70 (s, 1H, aromatic), 8.61 (d, *J* = 8.5 Hz, 1H), 8.31 (d, *J* = 8.6 Hz, 1H), 8.14 (d, *J* = 7.2 Hz, 1H), 7.94 (d, *J* = 7.5 Hz, 1H, aromatic), 7.90 (d, *J* = 8.3 Hz, 2H, aromatic), 7.83–7.78 (m, 2H, aromatic), 7.75 (dd, *J* = 17.1, 8.9 Hz, 1H, aromatic), 7.64 (t, *J* = 8.0 Hz, 1H, aromatic), 7.34 (dd, *J* = 14.3, 6.7 Hz, 1H, aromatic), 7.29 (dd, *J* = 15.3, 7.5 Hz, 2H, aromatic), 6.65 (d, *J* = 8.0 Hz, 1H, aromatic), 2.81 (s, 6H, N(CH_3_)_2_), Figure S22. ^13^C NMR (126 MHz, DMSO-d_6_): *δ* 163.24, 152.97, 148.94, 142.91, 133.79, 133.30, 132.88, 132.76, 132.30, 131.00, 130.78, 130.75, 130.31, 130.10, 129.04, 128.91, 127.78, 126.96, 124.78, 122.75, 119.22, 46.15, Figure S23. MALDI-TOF: [M+H]^+^ Calcd. for C_26_H_23_BrN_3_O_4_S 552.05872; found 552.05951, Figure S24. Elemental analysis: Calc. for C_26_H_22_BrN_3_O_4_S: C, 56.53; H, 4.01; N, 7.61; found: C, 55.95 ± 0.04; H, 4.05 ± 0.03; N, 6.96 ± 0.07.

*2-{(E)-[2-(4-Cyanobenzoyl)hydrazinylidene]methyl}phenyl 5-(dimethylamino)naphthalene-1-sulfonate* (**5g**): Yield: 88.6%, yellow solid; Rf: 0.52 (*n*-hexane/EtOAc = 60%), m.p.: 108.9–109.7 °C. IR (ATR, cm^−1^): 3231, 2943, 2833, 2787, 2229, 1653, 1609, 1567, 1501, 1479, 1450, 1367, 1271, 1203, 1162, 1147, 1060, 862, 788, 574. ^1^H NMR (500 MHz, DMSO-d_6_): *δ* 12.20 (s, 1H, NH), 8.72 (s, 1H, CH), 8.62 (d, *J* = 8.5 Hz, 1H, aromatic), 8.31 (t, *J* = 7.0 Hz, 1H, aromatic), 8.14 (d, *J* = 7.3 Hz, 1H, aromatic), 8.13–8.04 (m, 4H, aromatic), 7.97–7.92 (m, 1H, aromatic), 7.76 (t, *J* = 8.1 Hz, 1H, aromatic), 7.65 (t, *J* = 8.0 Hz, 1H, aromatic), 7.32 (ddd, *J* = 25.9, 17.1, 7.5 Hz, 3H, aromatic), 6.65 (d, *J* = 8.1 Hz, 1H, aromatic), 2.82 (s, 6H, N(CH_3_)_2_), Figure S25. ^13^C NMR (126 MHz, DMSO-d_6_): *δ* 162.86, 152.99, 149.00, 143.56, 138.29, 133.79, 132.89, 132.68, 130.80, 130.73, 130.30, 130.09, 129.75, 128.94, 128.90, 127.82, 124.79, 122.77, 119.47, 119.19, 116.86, 115.37, 46.16, Figure S26. MALDI-TOF: [M+H]^+^ Calcd. for C_27_H_23_N_4_O_4_S 499.14345; found 499.14423, Figure S27. Elemental analysis: Calc. for C_27_H_22_N_4_O_4_S, C, 65.05; H, 4.45; N, 11.24; found: C, 63.76 ± 0.32; H, 4.36 ± 0.01; N, 10.71 ± 0.04.

*2-{(E)-[2-(4-Methoxybenzoyl)hydrazinylidene]methyl}phenyl 5-(dimethylamino)naphthalene-1-sulfonate* (**5h**): Yield: 93.4%, yellow solid; Rf: 0.75 (*n*-hexane/EtOAc = 60%), m.p.: 104.2–105.5 °C. IR (ATR, cm^−1^): 3216, 2939, 2835, 1645, 1603, 1568, 1506, 1479, 1450, 1367, 1310, 1251, 1203, 1174, 1146, 1060, 1028, 945, 829, 788, 568. ^1^H NMR (500 MHz, DMSO-d_6_): *δ* 11.85 (s, 1H, NH), 8.68 (s, 1H, CH), 8.61 (d, *J* = 8.5 Hz, 1H, aromatic), 8.32 (d, *J* = 8.8 Hz, 1H, aromatic), 8.14 (d, *J* = 7.3 Hz, 1H, aromatic), 7.94 (d, *J* = 7.8 Hz, 3H, aromatic), 7.76 (t, *J* = 8.1 Hz, 1H, aromatic), 7.65 (t, *J* = 7.9 Hz, 1H, aromatic), 7.34 (q, *J* = 7.3 Hz, 1H, aromatic), 7.29 (t, *J* = 7.9 Hz, 2H, aromatic), 7.11 (d, *J* = 8.2 Hz, 2H, aromatic), 6.66 (d, *J* = 8.0 Hz, 1H, aromatic), 3.87 (s, 3H, OCH_3_), 2.81 (s, 6H, N(CH_3_)_2_), Figure S28. ^13^C NMR (126 MHz, DMSO-d_6_): *δ* 163.55, 163.33, 152.97, 148.83, 141.84, 133.77, 132.86, 132.22, 130.85, 130.79, 130.77, 130.31, 130.09, 129.25, 128.86, 127.70, 126.25, 124.78, 122.71, 119.24, 116.86, 114.93, 56.64, 46.15, Figure S29. MALDI-TOF: [M+H]^+^ Calcd. for C_27_H_26_N_3_O_5_S 504.15877; found 504.15289, Figure S30. Elemental analysis: Calc. for C_27_H_25_N_3_O_5_S: C, 64.40; H, 5.00; N, 8.34; found: C, 63.89 ± 0.11; H, 4.96 ± 0.04; N, 8.02 ± 0.09.

*2-{(E)-[2-(4-Methylbenzoyl)hydrazinylidene]methyl}phenyl 5-(dimethylamino)naphthalene-1-sulfonate* (**5i**): Yield: 93.1%, yellow solid; Rf: 0.68 (*n*-hexane/EtOAc = 60%), m.p.: 102.4–103.6 °C. IR (ATR, cm^−1^): 3215, 2942, 2833, 1646, 1610, 1568, 1556, 1479, 1368, 1271, 1203, 1185, 1146, 1060, 863, 788, 652. ^1^H NMR (500 MHz, DMSO-d_6_): *δ* 11.77 (s, 1H, NH), 8.70 (s, 1H, CH), 8.60 (d, *J* = 8.3 Hz, 1H, aromatic), 8.32 (d, *J* = 8.4 Hz, 1H, aromatic), 8.14 (d, *J* = 6.8 Hz, 1H, aromatic), 7.93 (d, *J* = 6.8 Hz, 1H, aromatic), 7.86 (d, *J* = 7.0 Hz, 2H, aromatic), 7.74 (t, *J* = 8.0 Hz, 1H, aromatic), 7.64 (t, *J* = 7.9 Hz, 1H, aromatic), 7.35 (dd, *J* = 21.3, 6.9 Hz, 3H, aromatic), 7.31–7.21 (m, 2H, aromatic), 6.66 (d, *J* = 7.5 Hz, 1H, aromatic), 2.80 (s, 6H, N(CH_3_)_2_), 2.40 (s, 3H, CH_3_), Figure S31. ^13^C NMR (126 MHz, DMSO-d_6_): *δ* 163.29, 152.28, 148.19, 142.47, 141.56, 133.03, 132.10, 131.55, 130.72, 130.19, 130.01, 129.64, 129.49, 129.43, 128.51, 128.20, 128.11, 127.04, 124.03, 122.01, 118.55, 116.13, 45.43, 21.52, Figure S32. MALDI-TOF: [M+H]^+^ Calcd. for C_27_H_26_N_3_O_4_S 488.16385; found 488.15805, Figure S33. Elemental analysis: Calc. for C_27_H_25_N_3_O_4_S: C, 66.51; H, 5.17; N, 8.62; found: C, 66.26 ± 0.14; H, 5.21 ± 0.01; N, 8.18 ± 0.02.

*2-{(E)-[2-(4-Methylbenzene-1-sulfonyl)hydrazinylidene]methyl}phenyl 5-(dimethylamino)naphthalene-1-sulfonate* (**5j**): Yield: 87.3%, yellow solid; Rf: 0.61 (*n*-hexane/EtOAc = 50%), m.p.: 83.7–84.2 °C. IR (ATR, cm^−1^): 3196, 2834, 1569, 1481, 1449, 1409, 1364, 1323, 1203, 1185, 1161, 1091, 1058, 943, 859, 778, 546, 531. ^1^H NMR (500 MHz, CDCl_3_): *δ* 11.71 (s, 1H, NH), 8.63 (d, *J* = 8.5 Hz, 1H, aromatic), 8.24 (d, *J* = 8.6 Hz, 1H, aromatic), 8.14 (d, *J* = 8.0 Hz, 2H, aromatic), 7.77–7.71 (m, 4H, aromatic), 7.65 (t, *J* = 8.0 Hz, 1H, aromatic), 7.41 (t, *J* = 7.3 Hz, 2H, aromatic), 7.31 (dd, *J* = 13.6, 5.6 Hz, 1H, aromatic), 7.25 (dt, *J* = 16.5, 9.5 Hz, 2H, aromatic), 6.61 (d, *J* = 7.8 Hz, 1H, aromatic), 2.86 (s, 6H, N(CH_3_)_2_), 2.36 (s, 3H, CH_3_), Figure S34. ^13^C NMR (125 MHz, CDCl_3_): *δ* 153.00, 148.46, 144.76, 141.48, 137.23, 133.80, 132.66, 132.49, 130.94, 130.83, 130.81, 130.53, 130.24, 130.11, 128.89, 128.69, 128.35, 127.32, 124.82, 122.77, 119.12, 116.98, 46.23, 22.19, Figure S35. MALDI-TOF: [M+H]^+^ Calcd. for C_26_H_26_N_3_O_5_S_2_ 524.13084; found 524.13184, Figure S36. Elemental analysis: Calc. for C_26_H_25_N_3_O_5_S_2_: C, 59.64; H, 4.81; N, 8.02; found: C, 58.84 ± 0.04; H, 4.92 ± 0.03; N, 8.22 ± 0.28.

### 3.2. Bioassays

#### 3.2.1. Cholinesterase Inhibition Assay

The modified Ellman’s method [78] was used to determine the inhibitory activity of the studied compounds against AChE and BChE. Acetylthiocholine (ATCh) was used as the substrate for AChE, while butyrylthiocholine (BTCh) was used for BChE. Uninhibited and inhibited substrate hydrolysis (ATCh/BTCh) catalyzed by AChE/BChE was carried out in phosphate-buffered saline (PBS, 0.1 M, pH 7.4) at room temperature. The enzyme activity in the final reaction mixture (2000 μL) was 0.2 U/mL, the concentration of ATCh/BTCh was 40 µM and the concentration of 5,5’-dithiobis(2-nitrobenzoic acid) (DTNB) was 100 µM. The investigated derivatives were dissolved in DMSO to a concentration of 0.01 M and diluted as needed with demineralized water (conductivity 3 μS, equipment supplier BKG Water Treatment, Hradec Kralove, Czech Republic). To determine the IC_50_ values of all studied compounds and RIV, GAL and DON, five different inhibitor concentrations were used in the final reaction mixture. All experiments were performed in triplicate. The time dependences of absorbance at a wavelength of 412 nm were monitored and the reaction rates for uninhibited (v_0_) and inhibited (v_i_) reactions were calculated. Subsequently, the dependence of v_0_/v_i_ on the inhibitor concentration was plotted and the IC_50_ values were calculated from the obtained regression curve equations for y = 2 (based on the definition of IC_50_).

The type of inhibition was also determined for the three most potent BChE inhibitors using the Lineweaver–Burk plot [58]. The measurement procedure was similar to that for IC_50_ determination. Reactions were performed in PBS (0.1 M, pH 7.4) at room temperature. The BChE activity in the final reaction mixture was 0.2 U/mL, the concentration of BTCh was 20–80 µM, and the concentration of DTNB was 100 µM. Again, the time dependences of absorbance at a wavelength of 412 nm were monitored in the presence and absence of inhibitor and the reaction rates were calculated. All experiments were performed in duplicate and the average values of reaction rates were used to construct the Lineweaver–Burk plot. From the obtained regression curve equations, the values of the Michaelis constant and the maximum velocity were calculated and the type of inhibition was determined.

#### 3.2.2. Cell Cultivation and Cytotoxicity Assessment

Modified human monocytic leukemia THP1-Blue™ NF-κB cells (InvivoGen, San Diego, CA, USA) were employed for in vitro antiproliferative assays. Cells were routinely maintained in RPMI 1640 medium supplemented with 10% fetal bovine serum (FBS), 2 mM L-glutamine, antibiotics (100 U/mL penicillin and 100 µg/mL streptomycin) (all reagents from Sigma-Aldrich), and the antimicrobial agent Normocin (InvivoGen, San Diego, CA, USA). Cultures were incubated at 37 °C in a humidified atmosphere containing 5% CO_2_ and were passaged twice weekly. Cell viability following treatment with the tested compounds was assessed using the Cell Counting Kit-8 (CCK-8; Sigma-Aldrich, Prague, Czech Republic) according to the manufacturer’s protocol. Sulfonate compounds were dissolved in dimethyl sulfoxide (DMSO; Sigma-Aldrich) and added to the cell suspension (5 × 10^5^ cells/mL) in RPMI 1640 medium devoid of FBS. Cells were incubated at 37 °C with 5% CO_2_ for 24 h. Subsequently, relative cell viability, defined as the ratio of viable treated cells to viable control cells (treated with DMSO only), was determined using the CCK-8 assay. Half-maximal inhibitory concentration (IC_50_) values, representing the concentration required to reduce relative cell viability by 50%, were calculated from viability curves using a four-parameter logistic (4PL) model in Prism 8.0.1 software (GraphPad, Boston, MA, USA). Viability assays were performed using at least five concentrations per compound, with all experiments conducted a minimum of three times in triplicate.

#### 3.2.3. Evaluation of NF-κB Activity

The impact of the tested compounds on the activity of the pro-inflammatory transcription factor NF-κB was assessed in lipopolysaccharide (LPS)-stimulated THP1-Blue™ NF-κB cells, as previously described [79]. Briefly, cells were pre-treated with various concentrations of the test compounds, dissolved in DMSO, for 1 h. Concentrations used were confirmed to be non-cytotoxic (cell viability > 95%). Subsequently, LPS from *Escherichia coli* O111:B4 (Merck), dissolved in serum-free RPMI 1640 medium at a final concentration of 0.5 µg/mL, was added to the cells. After 24 h of incubation, NF-κB activity was quantified by measuring the secretion of embryonic alkaline phosphatase using Quanti-Blue™ medium (InvivoGen).

Statistical analyses were carried out using GraphPad Prism 8.01 software (San Diego, CA, USA). The data were graphed as means ± standard error of mean (SE). Outlying values were identified by ROUT algorithms (Q = 5%) and removed from the analysis. Comparisons between groups were made using a one-way ANOVA test followed by the uncorrected Fisher’s LSD post hoc test for multiple comparisons.

### 3.3. ADME Study

The physicochemical properties of selected compounds were evaluated using the SwissADME [80]. It is an effective free web tool that allows the computation of physicochemical descriptors as well as the prediction of ADME parameters, pharmacokinetic properties, druglike nature and medicinal chemistry friendliness of one or multiple small molecules to support drug discovery.

The relationship between pharmacokinetic and physicochemical parameters was delineated by the so-called Lipinski’s rule of five [81]. It is a rule of thumb to evaluate drug-likeness or determine if a chemical compound with a certain pharmacological or biological activity has physicochemical properties that would likely make it an orally active drug in humans. It is based on the observation that most orally effective drugs are relatively small and moderately lipophilic molecules. The Lipinski’s rule of five states that orally active drugs have less than 5 hydrogen-bond donors, less than 10 (5 × 2) hydrogen-bond acceptors, a molecular weight less than 500, and a calculated log *P* (n-octanol/water partition coefficient) of less than 5. Violating more than one of these guidelines reduces the likelihood of adequate absorption.

In addition to log *P*, polar surface area (PSA) has been identified as another representative parameter for CNS-targeting drugs. A PSA value lower than 60–70 Å^2^ usually indicates active compounds [82]. According to Ref. [83], the suggested PSA ranges for increasing the probability of attaining improved BBB permeability are to be lower than 90 Å^2^, preferably lower than 70 Å^2^. Instead of the PSA value, the topological polar surface area (tPSA) is commonly used.

To perform ADME profiling, structures of the investigated compounds (created in ChemDraw 21.0.0.) were uploaded as input to the SwissADME server. These were subsequently converted to SMILES format. The output panel is divided into different sections: chemical structure and bioavailability radar, physicochemical properties, lipophilicity, water solubility, pharmacokinetics, drug-likeness, and medicinal chemistry. In addition, it is possible to display a graphical output, a BOILED-Egg model, which predicts the ability of the investigated compound to be passively absorbed in the GIT (white region) and passively cross the BBB (yellow region). The obtained outputs were processed into Table 2 (fulfillment of Lipinski’s rule of five) and Figure 5 (bioavailability) and Figure 6 (passive absorption in human GIT and passive crossing the BBB).

### 3.4. Molecular Modeling Study

#### 3.4.1. Automated Docking Setup

Crystal structures of human BChE (PDB ID 1P0I) [84] were retrieved from the RCSB Protein Data Bank and used as receptors. Prior to computation, all non-protein residues (including co-crystallized ligands and waters) were removed. Molecular docking was performed with AutoDock4 [85], keeping the receptor rigid while allowing full ligand flexibility. For BChE, cubic grids of 50 × 50 × 50 points were generated with a spacing of 0.375 Å and centered on the catalytic site of the enzyme. Ligands were assigned Gasteiger partial charges, nonpolar hydrogens were merged, and all rotatable bonds were left unconstrained. For each ligand, 200 poses were produced. The maximum number of energy evaluations and genetic algorithm generations were set to 2.5 × 10^6^ and 2.7 × 10^4^, respectively; all remaining parameters were left at their default values. Resulting poses were clustered by backbone root-mean-square deviation (RMSD) and ranked by predicted binding free energy; the representative of the most populated cluster with the lowest relative free energy was selected for subsequent analyses.

#### 3.4.2. Molecular Dynamic Simulation

MD simulations were conducted using the Amber22 package [86] with the ff14SB protein force field [87] and the General Amber Force Field (GAFF) for ligands [88]. Complexes were prepared with the Leap module, and ligand topologies were generated through the Antechamber utility. Each selected complex was embedded in an octahedral box of TIP3P explicit water molecules [89], ensuring at least a 10 Å buffer between the solute and the box boundaries. Counterions (Na^+^ or Cl^−^) were introduced to neutralize the net charge of BChE systems.

Energy minimization of the solvated complexes was performed for 5000 steps using the steepest descent algorithm implemented in the Sander module. Following minimization, equilibration was carried out under constant volume conditions for 2500 ps, during which the systems were gradually heated from 0 to 300 K using a Langevin thermostat [90] with a collision frequency of 1.0 ps^−1^. The SHAKE algorithm [91] was employed to constrain covalent bonds involving hydrogen atoms, enabling the use of a 2 fs integration time step.

Production MD trajectories were generated in triplicate at a target temperature of 298 K, each extending for 50 ns, resulting in a cumulative simulation time of 150 ns per complex. Long-range electrostatic interactions were treated with the Particle Mesh Ewald (PME) method [92], applying a 10 Å real-space cutoff, a grid spacing of 1.2 Å, and fourth-order spline interpolation. Trajectory post-processing and analysis were carried out using the CPPTRAJ module [93].

#### 3.4.3. MM-GBSA Free Energy Decomposition

Relative binding free energies of the enzyme–ligand complexes were estimated using the MM-PBSA [94] module implemented in AMBER22. Subsequently, per-residue free energy decomposition analyses were carried out to identify the key amino acid residues within the BChE active site that contribute to ligand interactions. For these calculations, snapshots were extracted from the final 30 ns of each MD trajectory, after removal of explicit solvent molecules and counterions.

#### 3.4.4. Quantum Theory of Atoms in Molecules

The representative conformations used for QTAIM [95,96] analyses were obtained through clustering of MD trajectories in CPPTRAJ [93], applying a 2 Å RMSD cutoff. For each complex, the most populated cluster was selected as the input structure. Topological analysis of the electron density within the QTAIM framework was then performed on reduced models of BChE bound to each ligand. Reduced models were generated by retaining only those residues directly interacting with the ligands, specifically including all amino acids located within 5 Å of any ligand atom. Wavefunctions for the reduced systems were computed at the M06-2X/6-31G(d) level of theory using Gaussian16 [97]. Subsequent QTAIM calculations were conducted with Multiwfn [98] to evaluate the electron density [ρ(r)] at the bond critical points (BCPs). The outcomes are reported as the summed ρ(r) values of BCPs, organized according to specific regions within the binding sites of the BChE.

## 4. Conclusions

We report here a new series of sulfonate derivatives with potent inhibitory activity against BChE and specific effects in relation to AChE. Another interesting aspect of these compounds is that they also showed anti-inflammatory activities, which might be important for a possible therapeutic use, especially for Alzheimer’s disease. It is important to note that these compounds were designed based on structural information through molecular modeling studies using combined techniques (docking, MD simulations, and QTAIM calculations). Although lead compound **3** showed the most potent inhibitory activity and good bioavailability not only from the GIT but also to the brain, it also displayed toxic effects, which rule it out as a good hit. However, this compound allowed us to take its structural skeleton to make modifications that allowed us to obtain compounds **5c** and **5b**, which did not show toxic effects and maintained strong inhibitory effects on BChE. Moreover, compound **5b** also exhibits some anti-inflammatory potential. Our results indicate that these two compounds are excellent starting structures for the development of new specific BChE inhibitors.

Important information related to ADME characteristics was evaluated for the most characteristic compounds in this series. Thus, some interesting physicochemical properties such as molecular weights, log *p* values, number of H-donors, number of H-acceptors and tPSA values are reported here. It is interesting to note that compounds reported here satisfy Lipinski’s rule of five. On the other hand, both compounds **5b**, **5c** have limited passive absorption into the brain (through the BBB), so there is room for further optimization of the structure with improved bioavailability to the brain or for innovative galenic modifications for increased delivery to the brain.

From a methodological perspective, it has also been possible to draw some interesting conclusions, at least for the type of compounds we are reporting here. Our results show that very simple techniques such as docking calculations and toxicity prediction programs can be very useful if used in a guiding and exploratory manner. In any case, modeling studies using more precise techniques, as well as experimental corroboration, are necessary. Meanwhile, QTAIM calculations to evaluate the molecular interactions of the different complexes in more detail have proven extremely useful, as they guide the design of new structures based on the information at the molecular level they provide. However, it is important to keep in mind that this type of calculation is highly dependent on the geometry used; therefore, its performance will depend largely on the data obtained through MD simulations and the corresponding residue analyses performed.

## Figures and Tables

**Table 1:** List of compounds investigated and their inhibitory activities and selectivity index (SI) compared to rivastigmine (RIV), galantamine (GAL), and donepezil (DON).

Comp.	Structure	IC_50_ (µM)		SI *
		AChE	BChE	
**3**	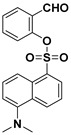	52.21 ± 2.07	0.74 ± 0.01	70.55
**5a**	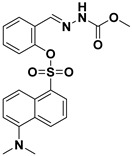	66.76 ± 0.53	27.69 ± 0.08	2.41
**5b**	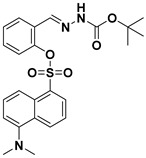	66.50 ± 0.39	11.51 ± 0.17	5.78
**5c**	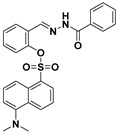	63.73 ± 1.83	1.11 ± 0.02	57.41
**5d**	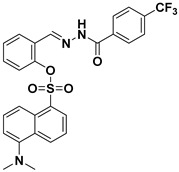	90.65 ± 2.66	212.06 ± 18.03	0.43
**5e**	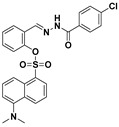	48.09 ± 0.28	88.72 ± 4.47	0.54
**5f**	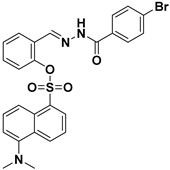	64.30 ± 1.43	116.37 ± 2.48	0.55
**5g**	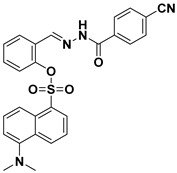	92.49 ± 3.26	178.61 ± 4.10	0.52
**5h**	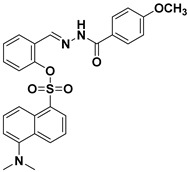	71.33 ± 2.00	101.89 ± 5.72	0.70
**5i**	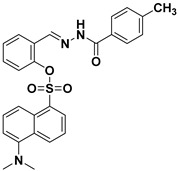	39.52 ± 1.27	30.24 ± 0.77	1.31
**5j**	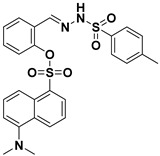	84.02 ± 2.43	188.84 ± 7.92	0.45
**RIV**	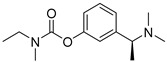	56.10 ± 1.41	38.40 ± 1.97	1.46
**GAL**	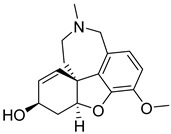	1.54 ± 0.02	2.77 ± 0.15	0.56
**DON**	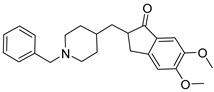	0.015 ± 0.0001	0.0314 ± 0.001	0.48

* SI—ratio IC_50_(AChE)/IC_50_(BChE).

**Table 2:** Physicochemical properties, Lipinski’s rule of five parameters, gastrointestinal absorption and bioavailability of compounds **3**, **5a**–**c**, **5e**, **5f** and **5i**.

Comp.	MW	Clog*P*	HBD	HBA	Violation	tPSA [Å^2^]	GI Absorption	Bioavailability Score
**3**	355.41	3.36	0	4	0	72.06	high	0.55
**5a**	427.47	3.28	1	6	0	105.68	high	0.55
**5b**	469.55	4.15	1	6	0	105.68	low	0.55
**5c**	473.54	4.41	1	5	0	96.45	high	0.55
**5e**	507.99	4.86	1	5	1	96.45	low	0.17
**5f**	552.44	4.95	1	5	1	96.45	low	0.17
**5i**	487.57	4.61	1	5	0	96.45	high	0.55
RIV	250.34	2.34	0	3	0	32.78	high	0.55

MW—molecular weight; Clog*P*—consensus log*P*_o/w_ (the arithmetic mean of the values predicted by the five proposed methods—XLOGP3, WLOGP, MLOGP, SILICOS-IT, and iLOGP); tPSA—topological polar surface area; HBA—number of H-bond acceptors; HBD—number of H-bond donors; GI—gastrointestinal; and RIV—rivastigmine.

## Data Availability

The original contributions presented in this study are included in the article/Supplementary Materials. Further inquiries can be directed to the corresponding authors.

## Supplementary Materials

The following supporting information can be downloaded at: https://www.mdpi.com/article/10.3390/ijms27073109/s1.

## Abbreviations

The following abbreviations are used in this manuscript:

AChE	Acetylcholinesterase
AD	Alzheimer’s Disease
ADME	Absorption, Distribution, Metabolism, Excretion
ANOVA	ANalysis Of Variance
ATR	Attenuated Total Reflection
BBB	blood–brain Barrier
BChE	Butyrylcholinesterase
BTCh	Butyrylthiocholine
CAS	Catalytic Anionic Site
CDCl_3_	Deuterated chloroform
CD_2_Cl_2_	Deuterated dichloromethane
CNS	Central Nervous System
DBH	2,5-Dihydrobenzoic acid
DCM	Dichloromethane
DON	Donepezil
DMSO	Dimethyl sulfoxide
EtOAc	Ethyl acetate
FBS	Fetal Bovine Serum
GAFF	General Amber Force Field
GAL	Galanthamine
GI	Gastrointestinal
GIT	Gastrointestinal tract
HBA	H-bond acceptors
HBD	H-bond donors
HRMS	High-Resolution Mass Spectrometry
IC	Inhibitory Concentration
IR	Infrared
*J*	Coupling constants
K_M_	Michaelis constant
LD	Lethal Dose
log *P*	logarithm of n-octanol-water partition coefficient
LPS	Lipopolysaccharide
MD	Molecular Dynamic
m.p.	Melting point
MW	Molecular Weight
NMR	Nuclear Magnetic Resonance
PAS	Peripheral Anionic Site
PME	Particle Mesh Ewald
PSA	Polar Surface Area
QTAIM	Quantum Theory of Atoms in Molecules
Rf	Retention Factor
RIV	Rivastigmine
RMSD	Root-Mean-Square Deviation
RPMI	Roswell Park Memorial Institute
SAR	structure–activity relationship
SEM	Standard Error Of Mean
SI	Selectivity Index
THP1	Human monocytic leukemia
TLC	Thin-Layer Chromatography
TMS	Tetramethylsilane
tPSA	topological Polar Surface Area
UV	Ultraviolet
V_m_	Maximum velocity
