# Supplementary material for: Structure-Based Design of New Series of Sulfonates with Potent and Specific BChE Inhibition and Anti-Inflammatory Effects
Source: Int J Mol Sci. 2026 Mar 29;27(7):3109. doi: 10.3390/ijms27073109 (PMC13072879; doi:10.3390/ijms27073109)
Supplement: Supplementary file 1 [file ijms-27-03109-s001.zip › ijms-4166880-supplementary.pdf]

Supplementary Materials

Structure-Based Design of New Series of Sulfonates with Potent and Specific BChE Inhibition and Anti-Inflammatory Effects

Siva Hariprasad Kurma [1], Camila Adarvez-Feresin [2], Oscar Parravicini [2], Adriana Garro [2,*], Sarka Stepankova [3], Jan Hosek [4], Karel Pauk [1], Jovana Lisicic [4,5], Josef Jampilek [6,7], Ricardo Daniel Enriz [2] and Ales Imramovsky [1,*]

1 Institute of Organic Chemistry and Technology, Faculty of Chemical Technology, University of Pardubice, Studentska 573, 532 10 Pardubice, Czech Republic; sivahari1415@gmail.com (S.H.K.), karel.pauk@upce.cz (K.P.)
2 Instituto Multidisciplinario de Investigaciones Biológicas (IMIBIO-SL), Facultad de Química, Bioquímica y Farmacia, Universidad Nacional de San Luis, Chacabuco 915, San Luis 5700, Argentina; cami-
3 laadarvezferesin@gmail.com (C.A.-F.), oparravicini@gmail.com (O.P.), denriz@unsl.edu.ar (R.D.E.)
Department of Biological and Biochemical Sciences, Faculty of Chemical Technology,
4 University of Pardubice, Studentska 573, 532 10 Pardubice, Czech Republic; sarka.stepankova@upce.cz
Department of Pharmacology and Toxicology, Veterinary Research Institute, Hudcova 296/70, 621 00 Brno, Czech Republic; hosek.jan@vri.cz (J.H.); 507401@mail.muni.cz (J.L.)
5 Department of Molecular Pharmacy, Faculty of Pharmacy, Masaryk University, Palackeho tr. 1946/1, 612 00 Brno, Czech Republic
6 Department of Chemical Biology, Faculty of Science, Palacky University Olomouc, Slechtitelu 27, 779 00 Olomouc, Czech Republic; josef.jampilek@gmail.com
7 Institute of Chemistry, University of Silesia, Szkolna 9, 40-007 Katowice, Poland

* Correspondence: adgarro@unsl.edu.ar (A.G.); ales.imramovsky@upce.cz (A.I.)

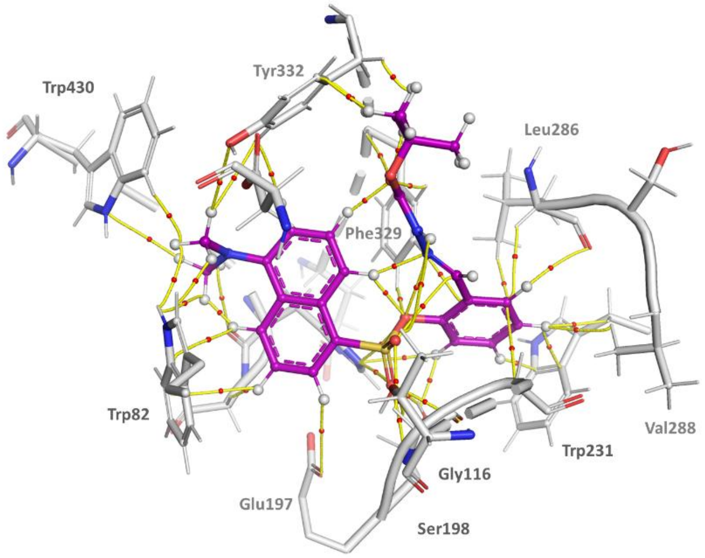

Figure S1. Charge density molecular graph of compound **5b** (purple) at the binding site of BChE. Residues from the active site are shown in grey. Topological elements of charge density associated with intermolecular interactions are depicted with yellow lines (bond paths) and small red spheres (bond critical points).

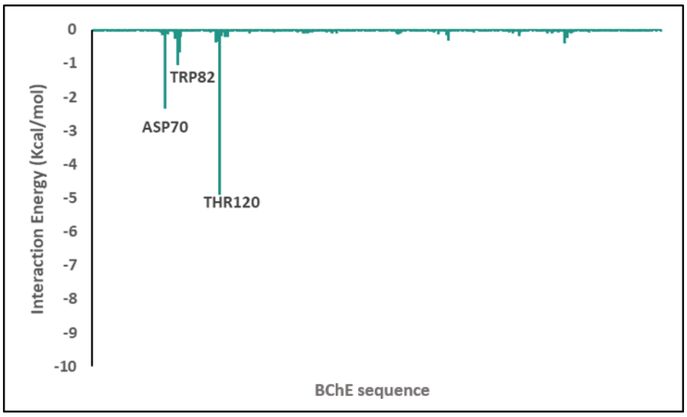

Figure S2. Histogram of interaction energies partitioned with respect to BChE amino acid sequence when complexed with compound **5d**. The X-axis denotes the residue number of BChE, and the Y-axis denotes the interaction energy between the compound and a specific residue.

List of used chemicals:

Compound	CAS Number	Supplier	Catalogue Number
Salicylaldehyde	90-02-8	Sigma-Aldrich	S356
5-(Dimethylamino)naphthalene-1-sulfonyl chloride	605-65-2	Thermo Fisher Scientific	115850250
Triethylamine	121-44-8	Acros Organics	219510010
Methyl hydrazinecarboxylate	6294-89-9	Sigma-Aldrich	151653
tert-Butyl hydrazinecarboxylate	870-46-2	Sigma-Aldrich	B91005
Benzohydrazide	613-94-5	Sigma-Aldrich	B13071
4-(Trifluoromethyl)benzohydrazide	339-59-3	TCI Europe	T3372
4-Methoxybenzohydrazide	3290-99-1	TCI Europe	M1770
4-Methylbenzohydrazide	3619-22-5	TCI Europe	M2846
4-Methylbenzenesulfonohydrazide	1576-35-8	Sigma-Aldrich	132004

Solvents

Solvent	CAS Number	Supplier	Catalogue Number
Ethyl acetate (p.a.)	—	Lach-Ner	—
n-Hexane (p.a.)	—	Lach-Ner	—
$CDCl_3$	865-49-6	Acros Organics	351421000
CD_2Cl_2	1665-00-5	Sigma-Aldrich / Fluorochem	177865 / D023
DMSO	2206-27-1	Sigma-Aldrich	175943

Other Materials

Material	Specification	Supplier	Catalogue Number
TLC plates	Silica gel F254	Merck	1.05554.0001
Silica gel (SiO_2)	0.035–0.070 mm, 60 Å	Acros Organics	240360300

Listo f ¹H and ¹³C NMR of prepared compounds:

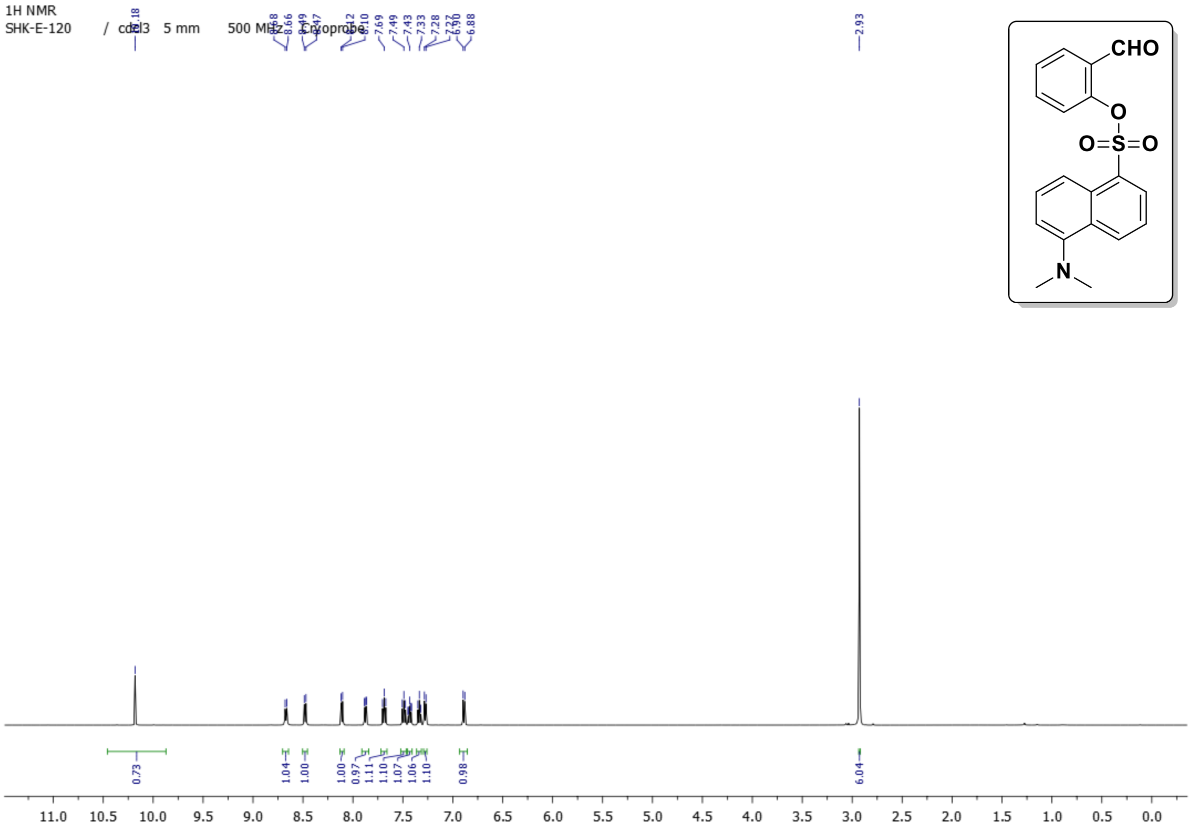

Figure S3. ¹H NMR Spectrum of compound **3**.

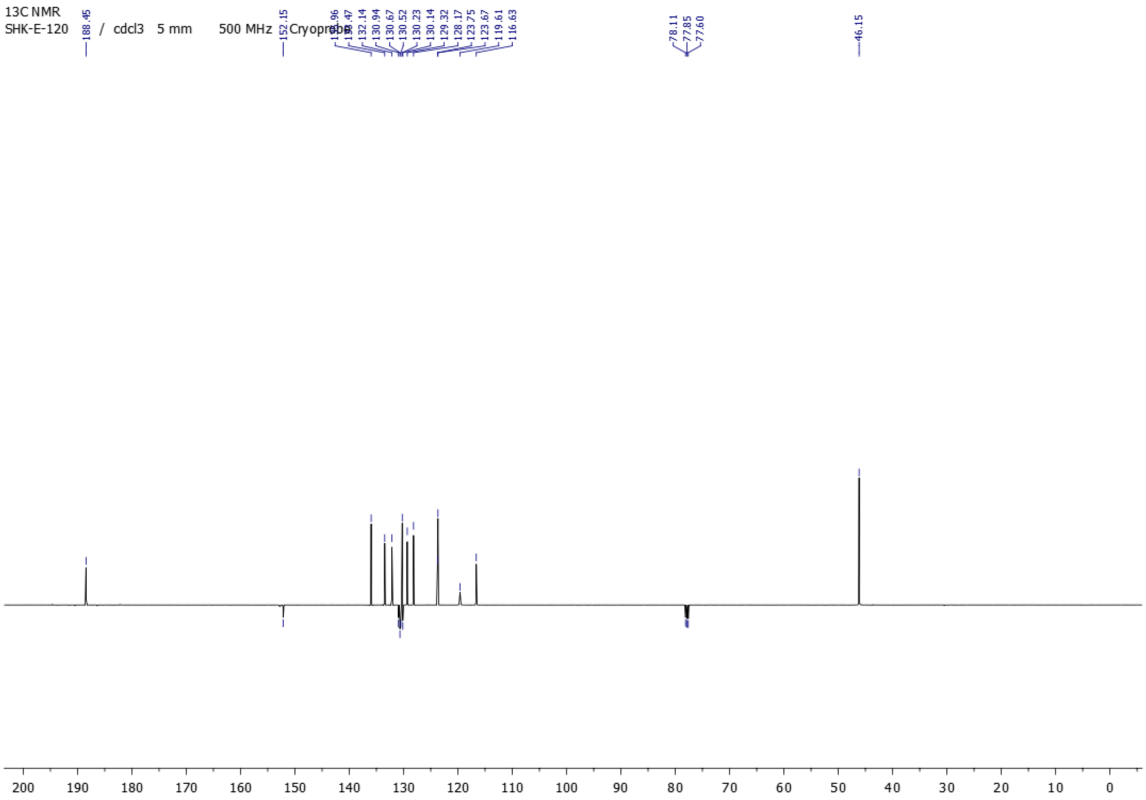

Figure S4. ¹³C NMR Spectrum of compound **3**.

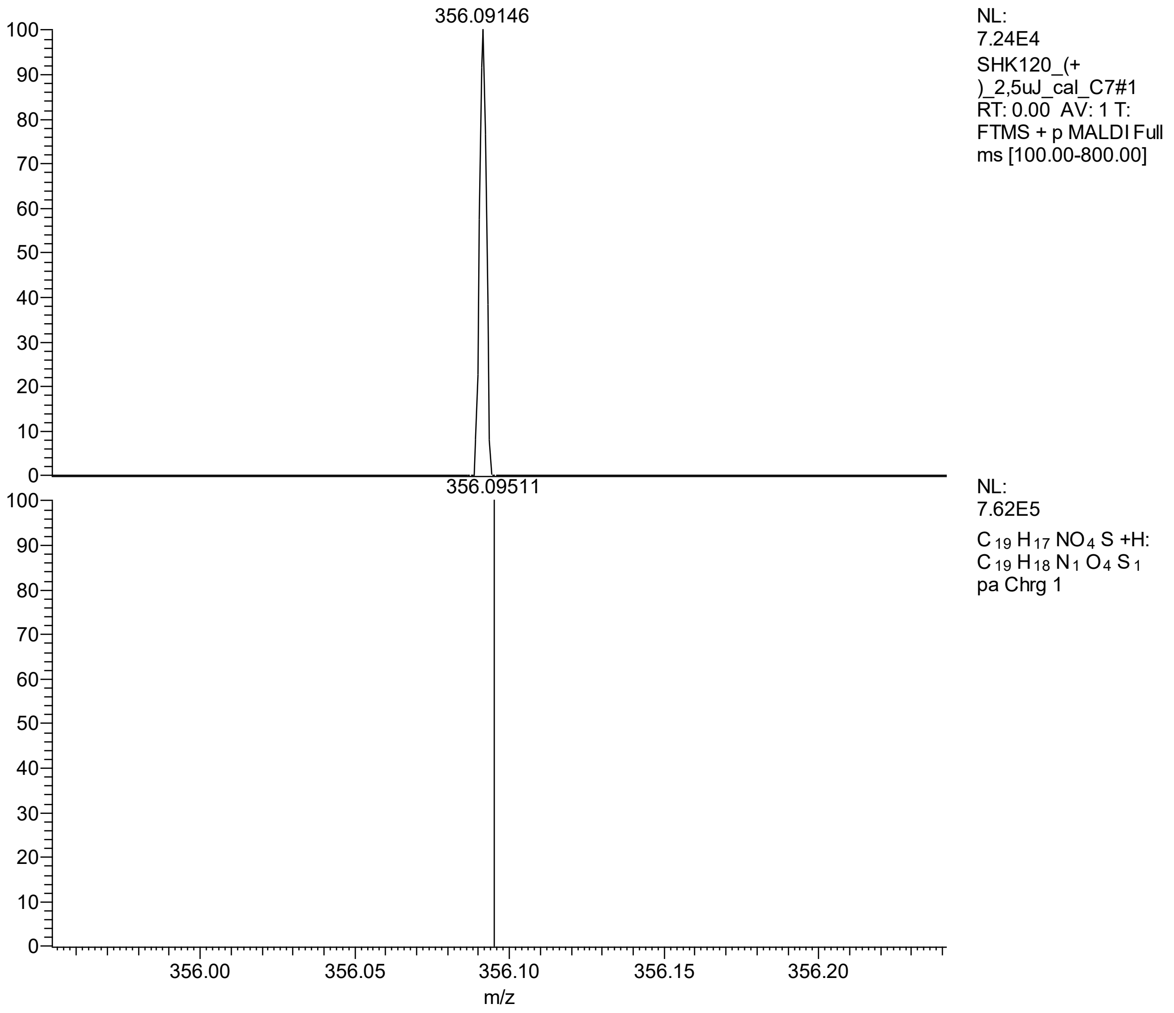

Figure S5. HRMS of compound **3**.

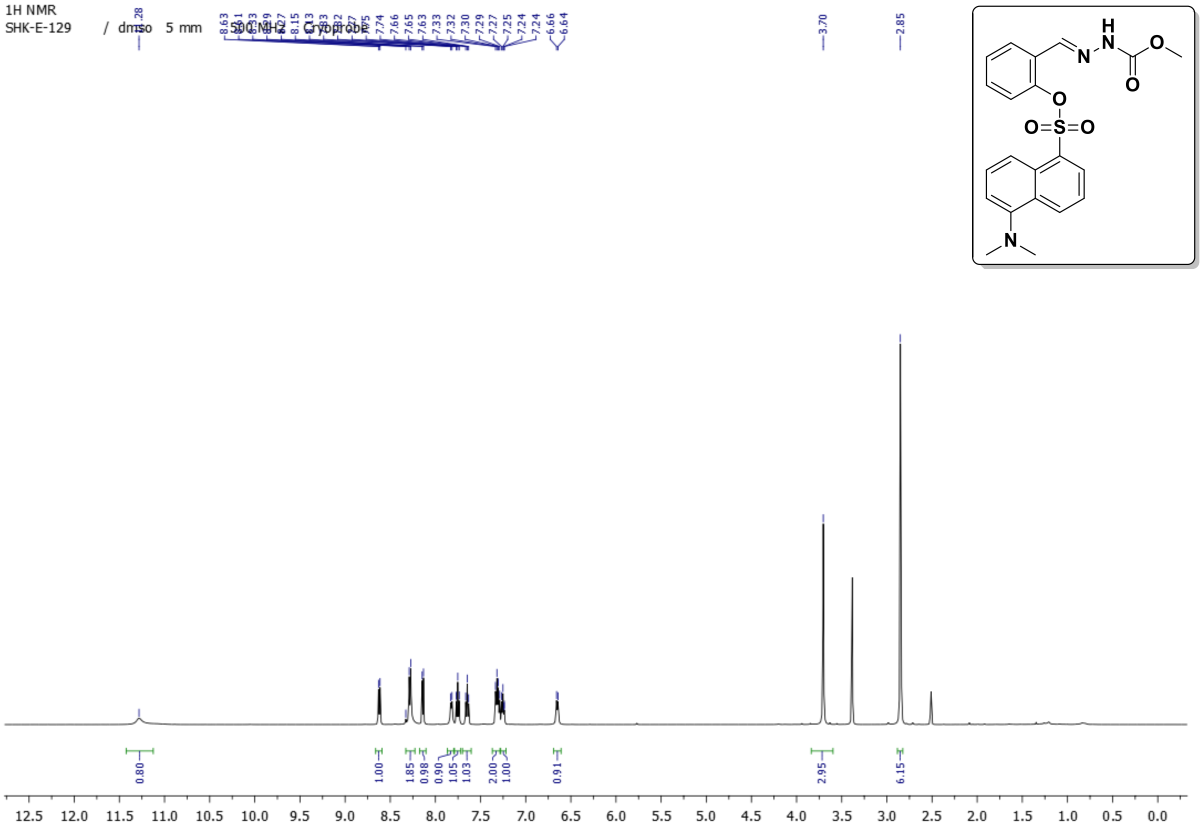

Figure S6. ^{1}H NMR Spectrum of compound **5a**.

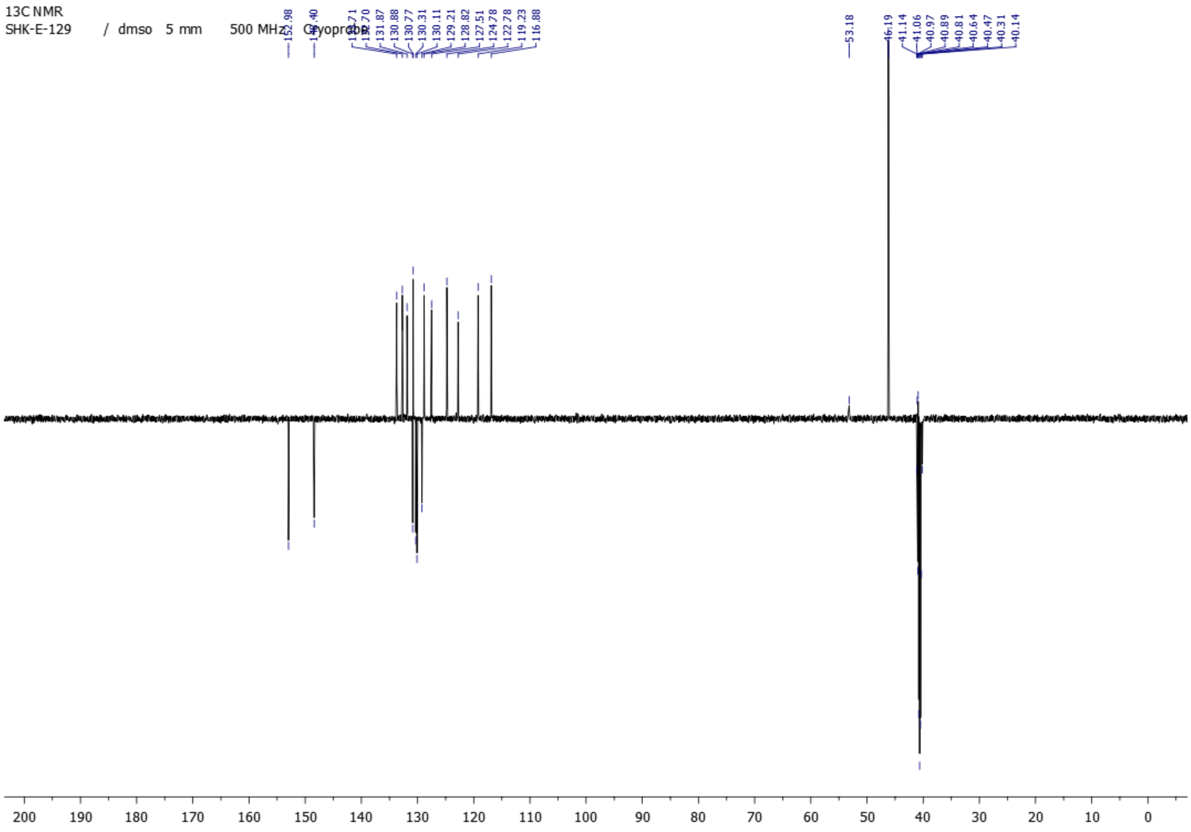

Figure S7. ^{13}C NMR Spectrum of compound **5a**.

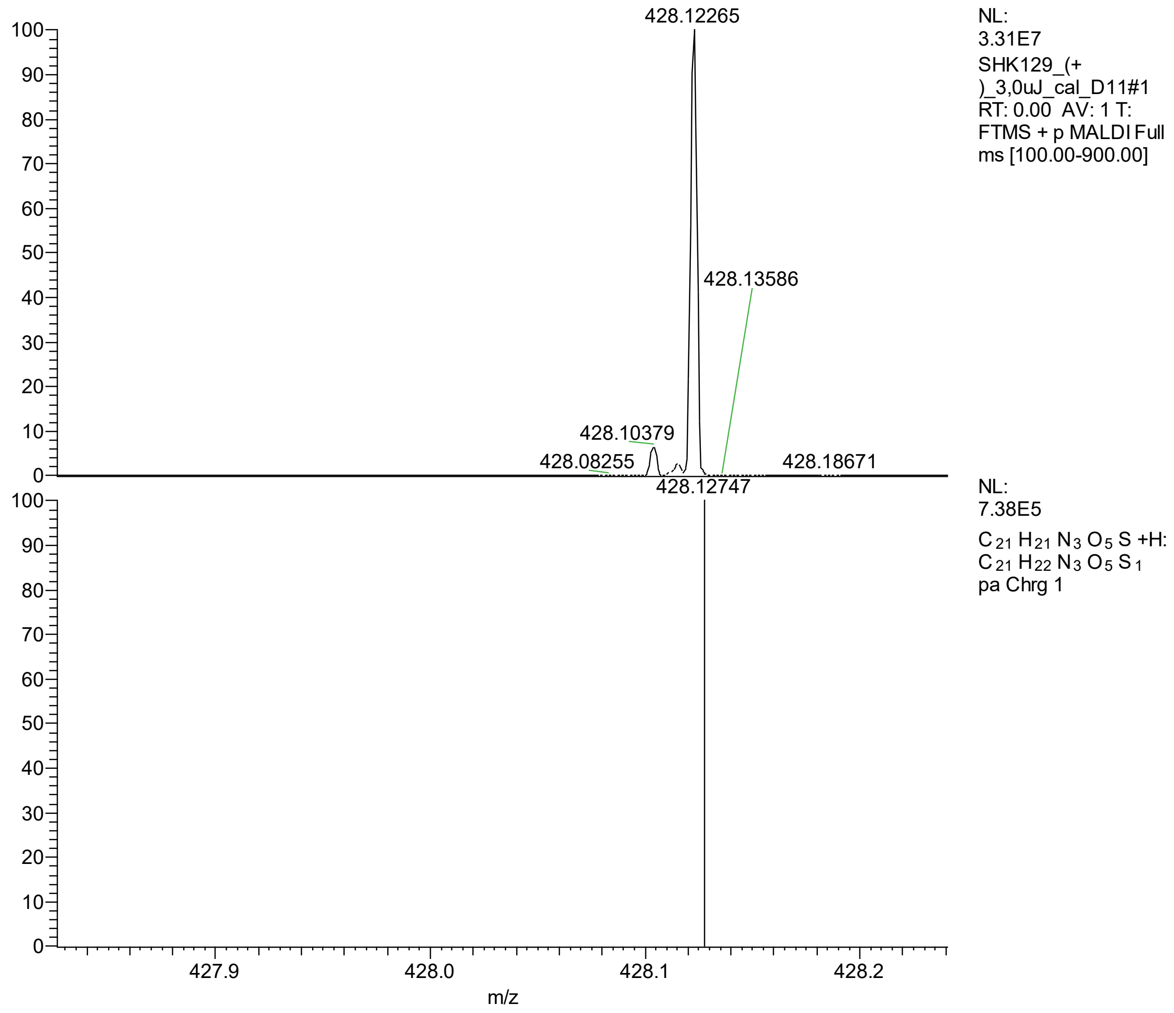

Figure S8. HRMS of compound **5a**.

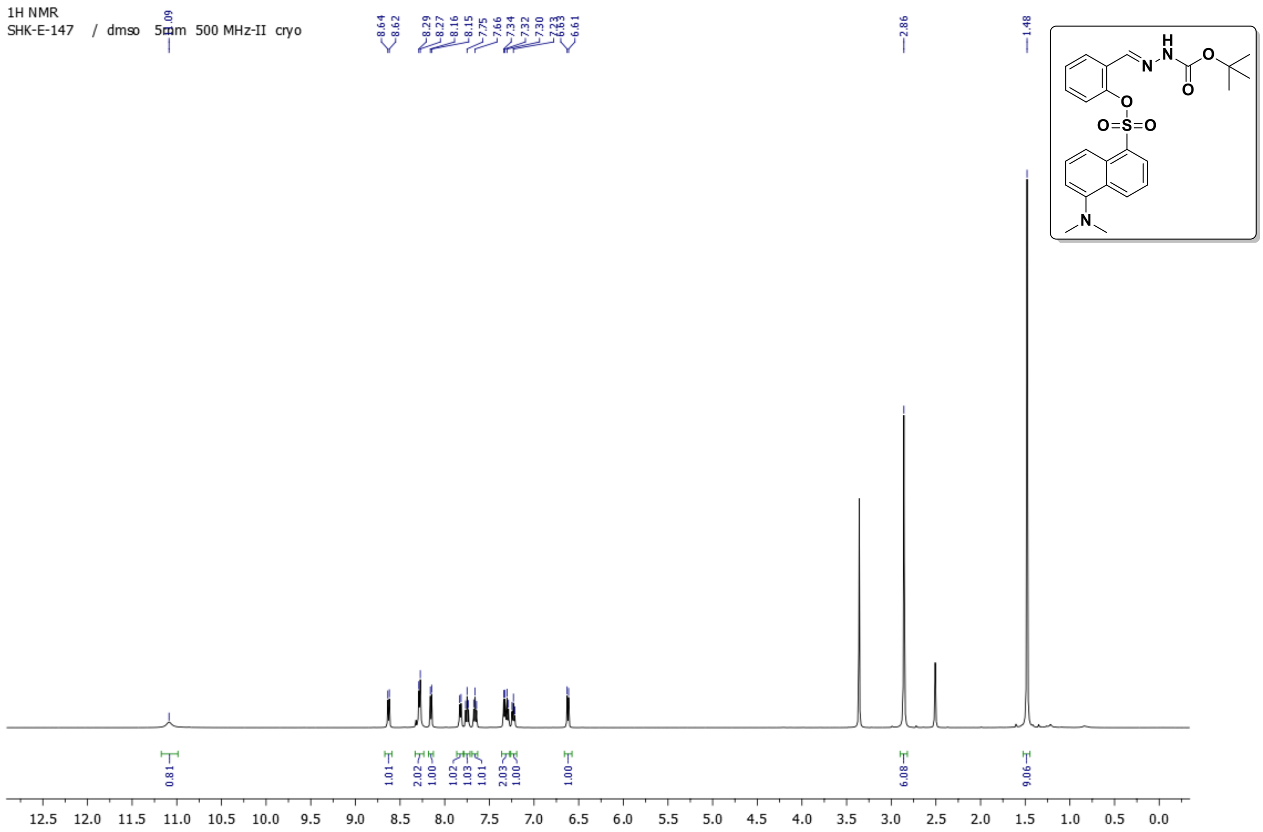

Figure S9. ^{1}H NMR Spectrum of compound **5b**.

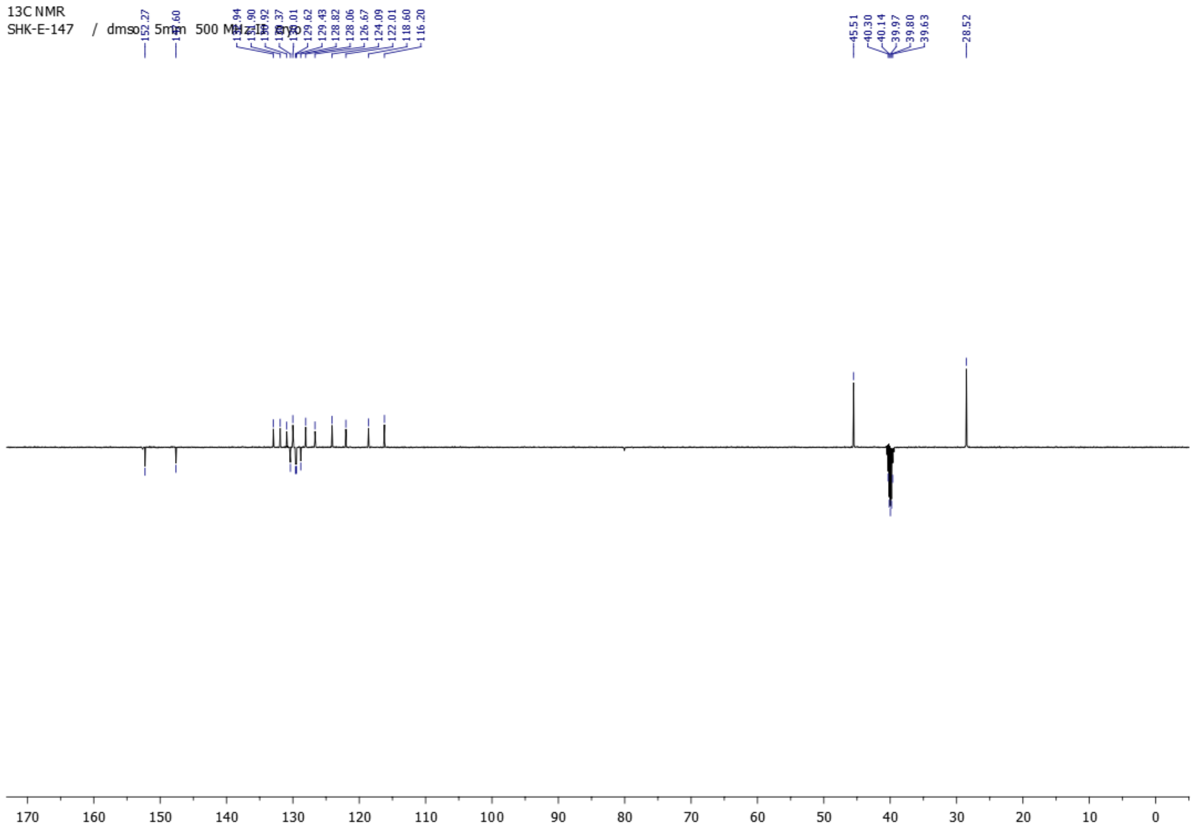

Figure S10. ^{13}C NMR Spectrum of compound **5b**.

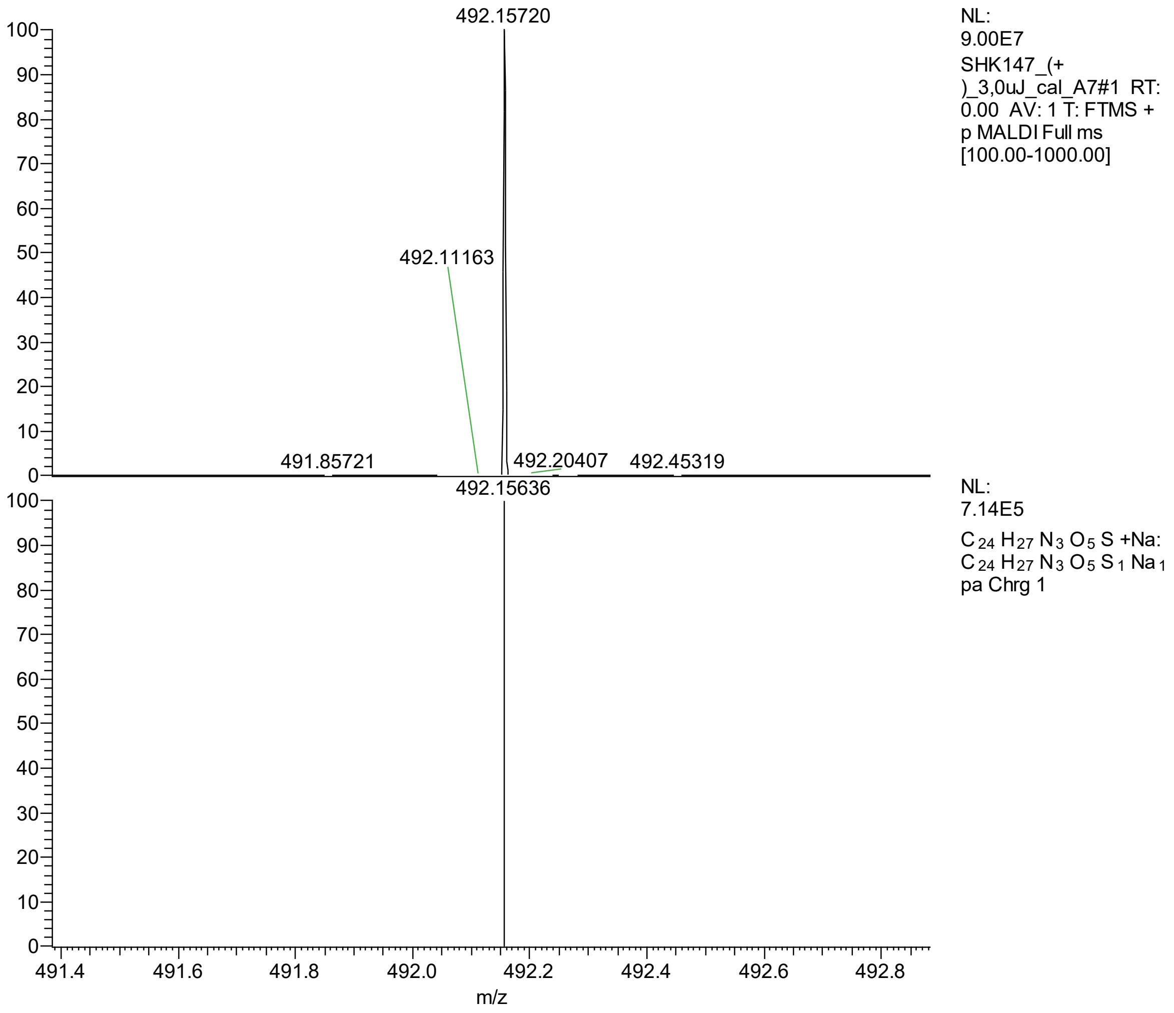

Figure S11. HRMS of compound **5b**.

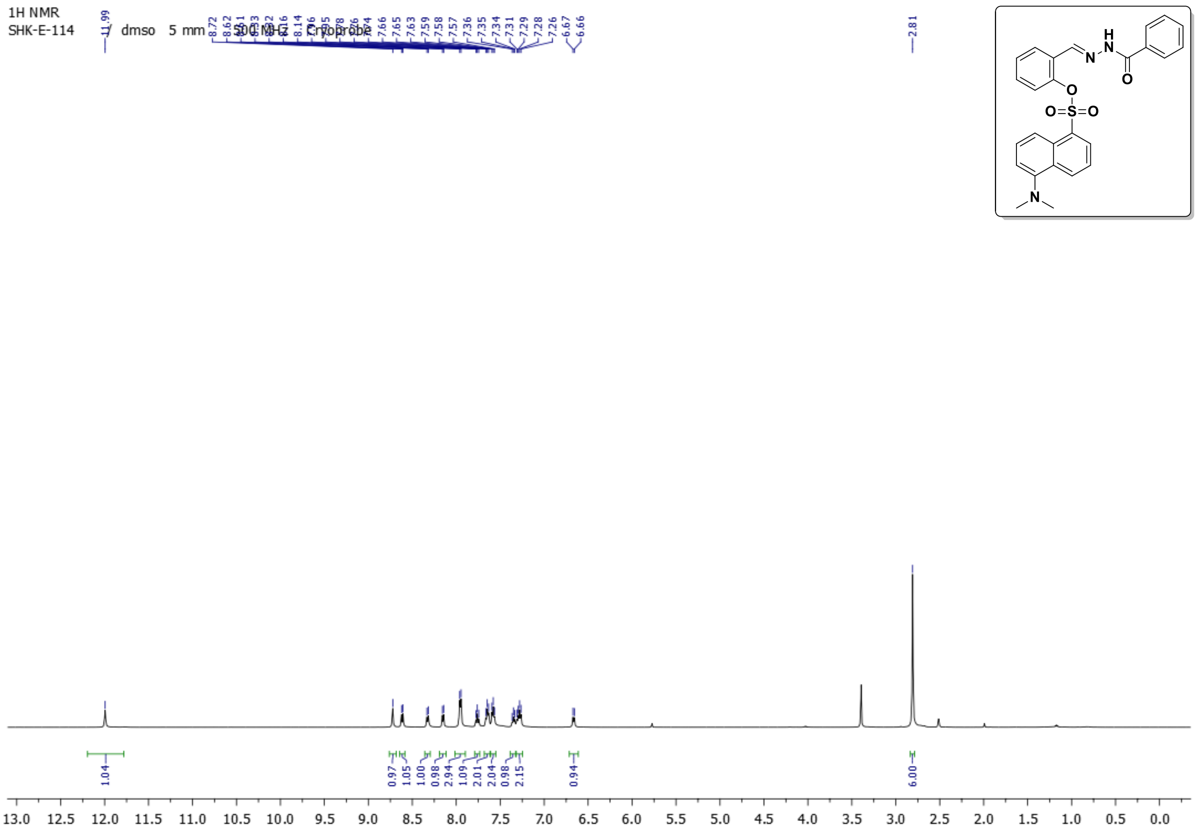

Figure S12. ^{1}H NMR Spectrum of compound **5c**.

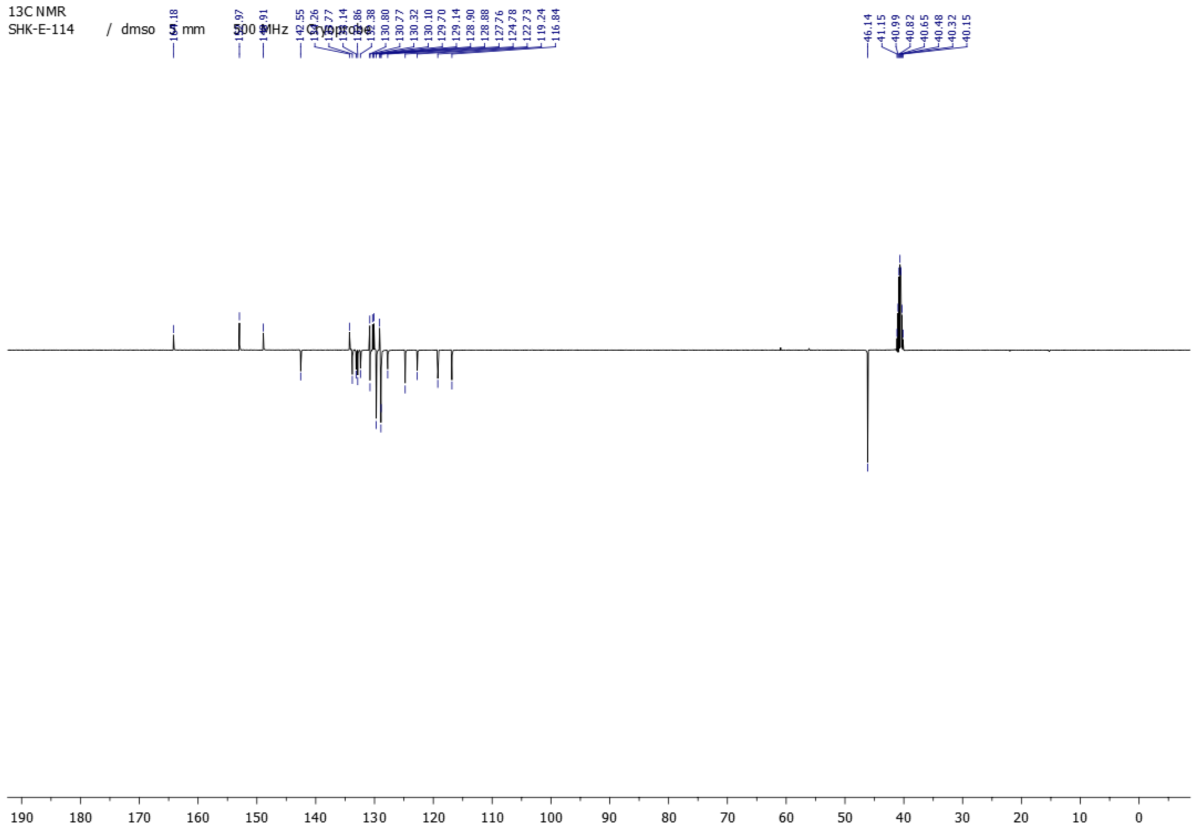

Figure S13. ^{13}C NMR Spectrum of compound **5c**.

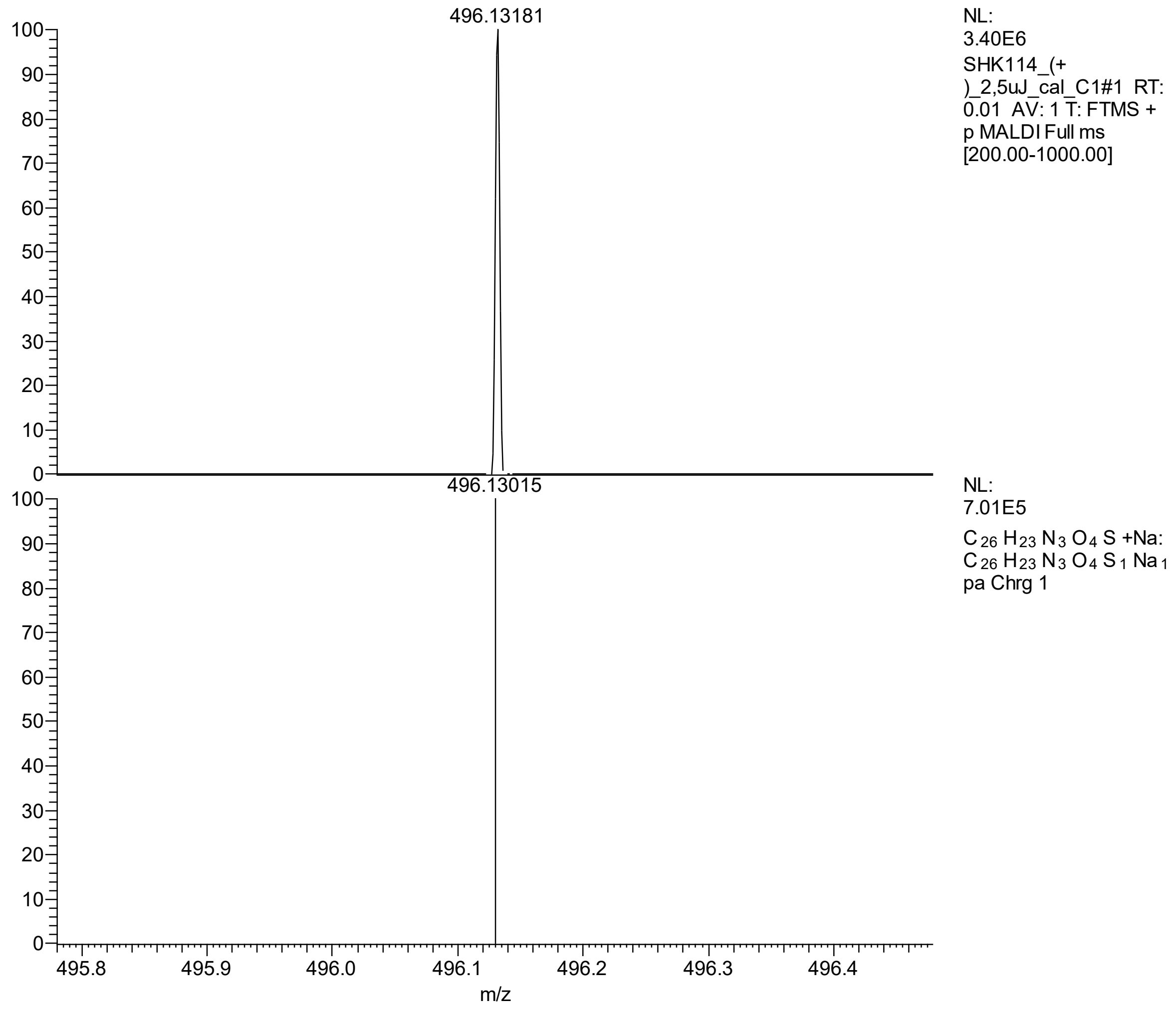

Figure S14. HRMS of compound **5c**.

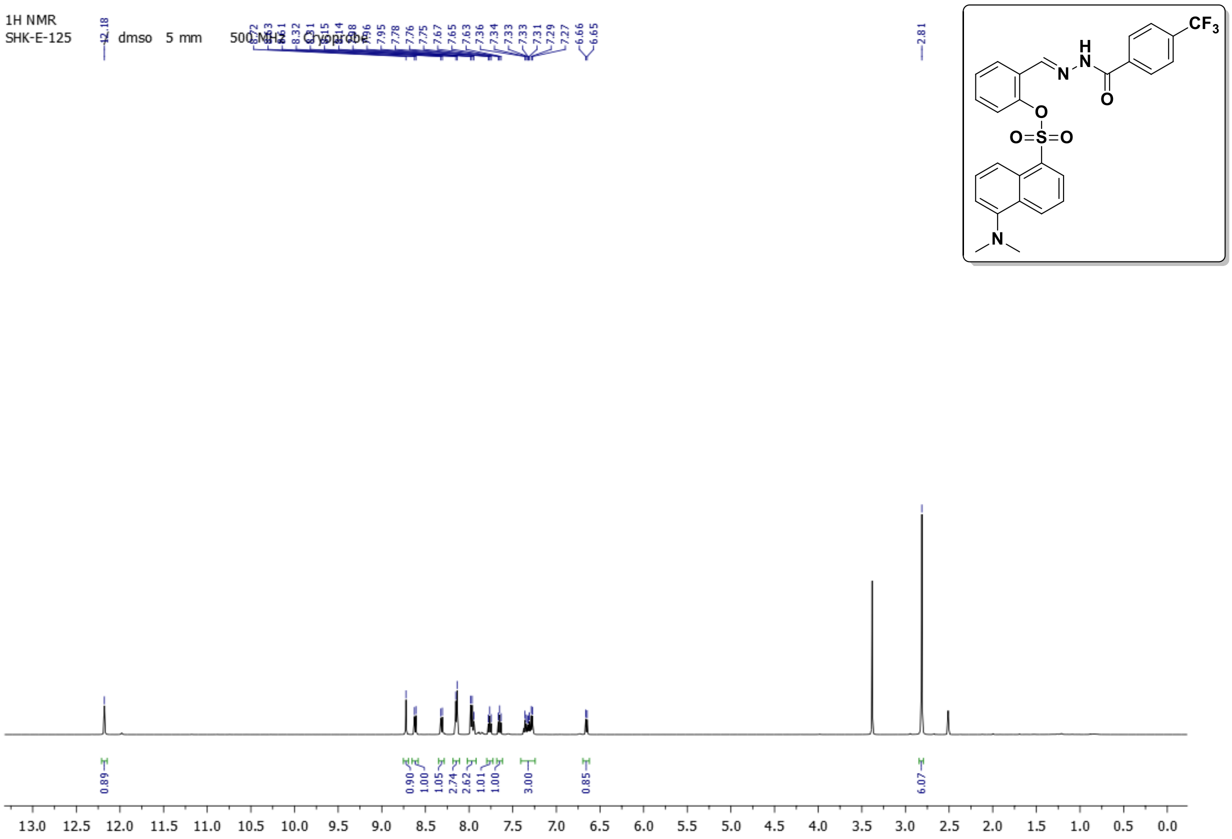

Figure S15. ^{1}H NMR Spectrum of compound **5d**.

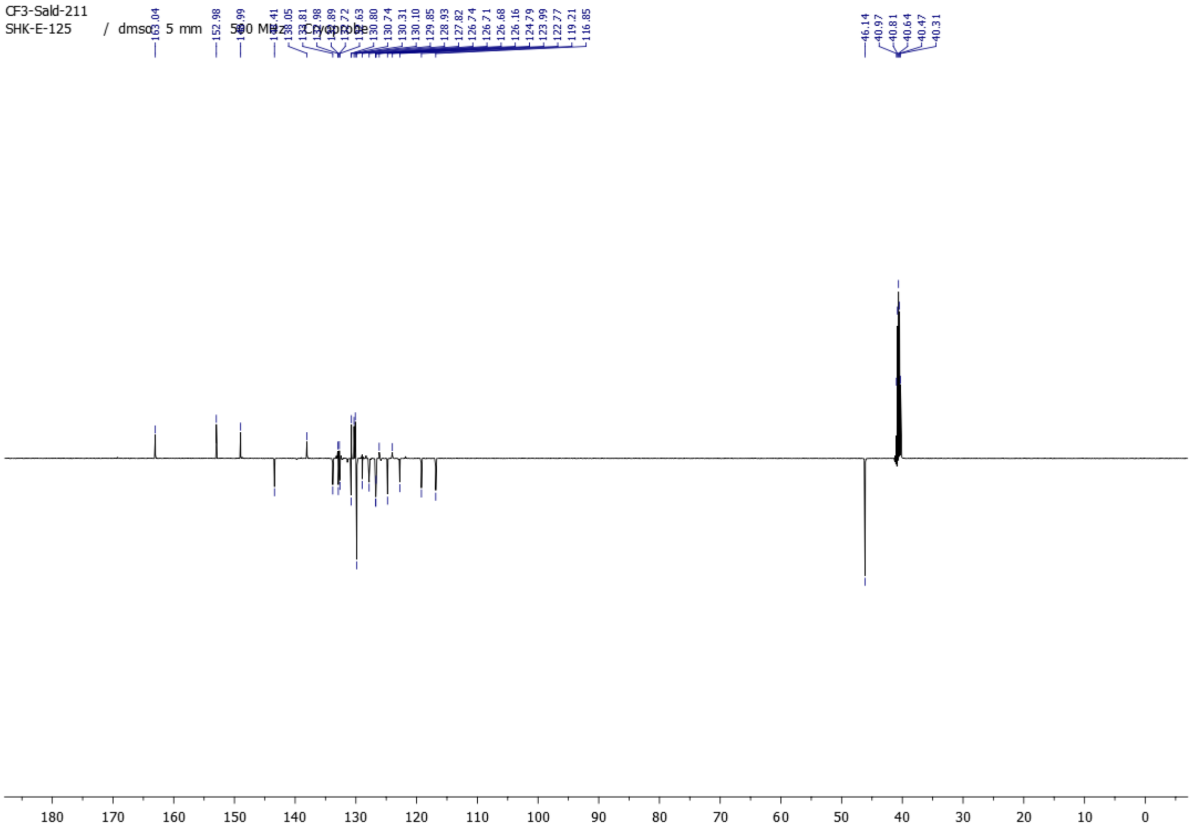

Figure S16. ^{13}C NMR Spectrum of compound **5d**.

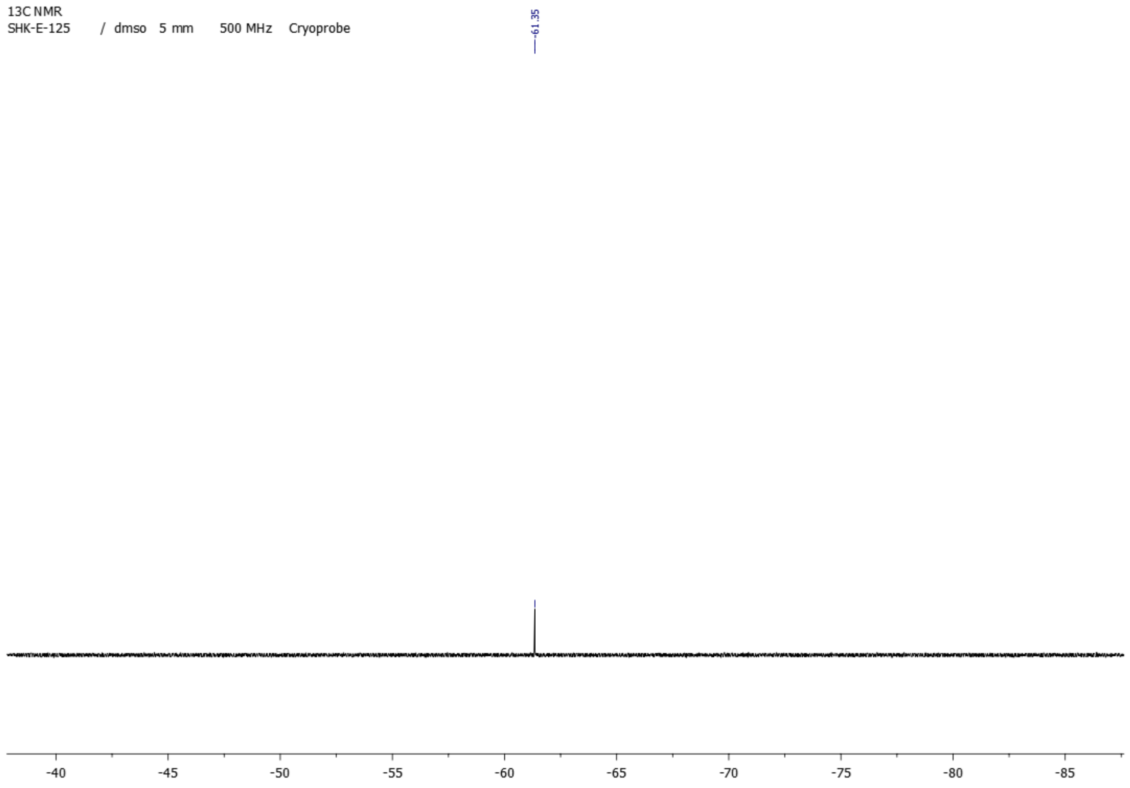

Figure S17. ^{19}F NMR Spectrum of compound **5d**.

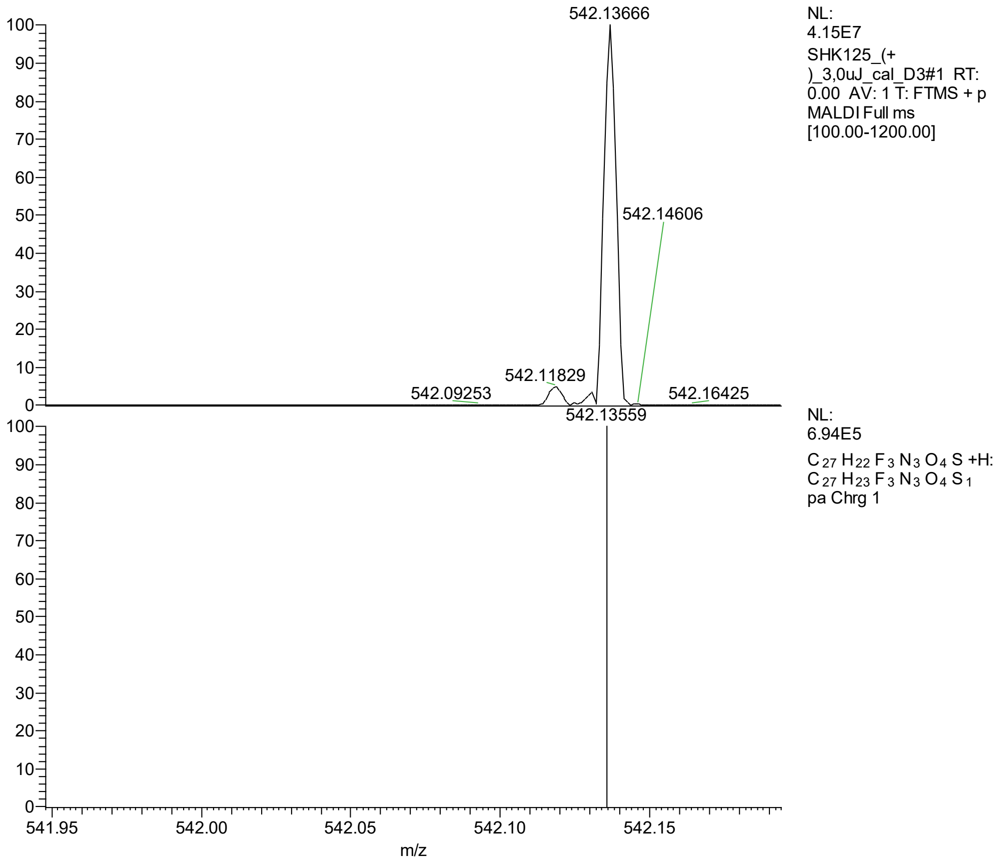

Figure S18. HRMS of compound **5d**.

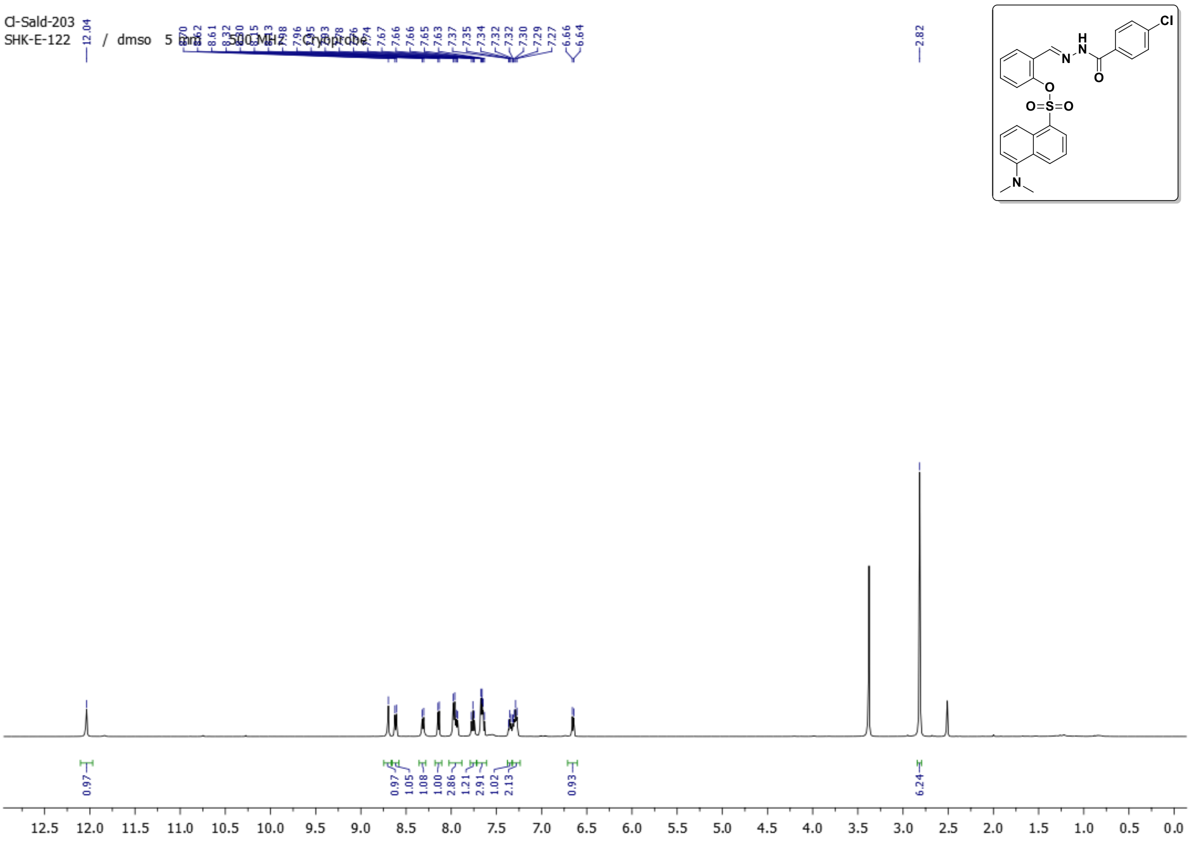

Figure S19. ¹H NMR Spectrum of compound **5e**.

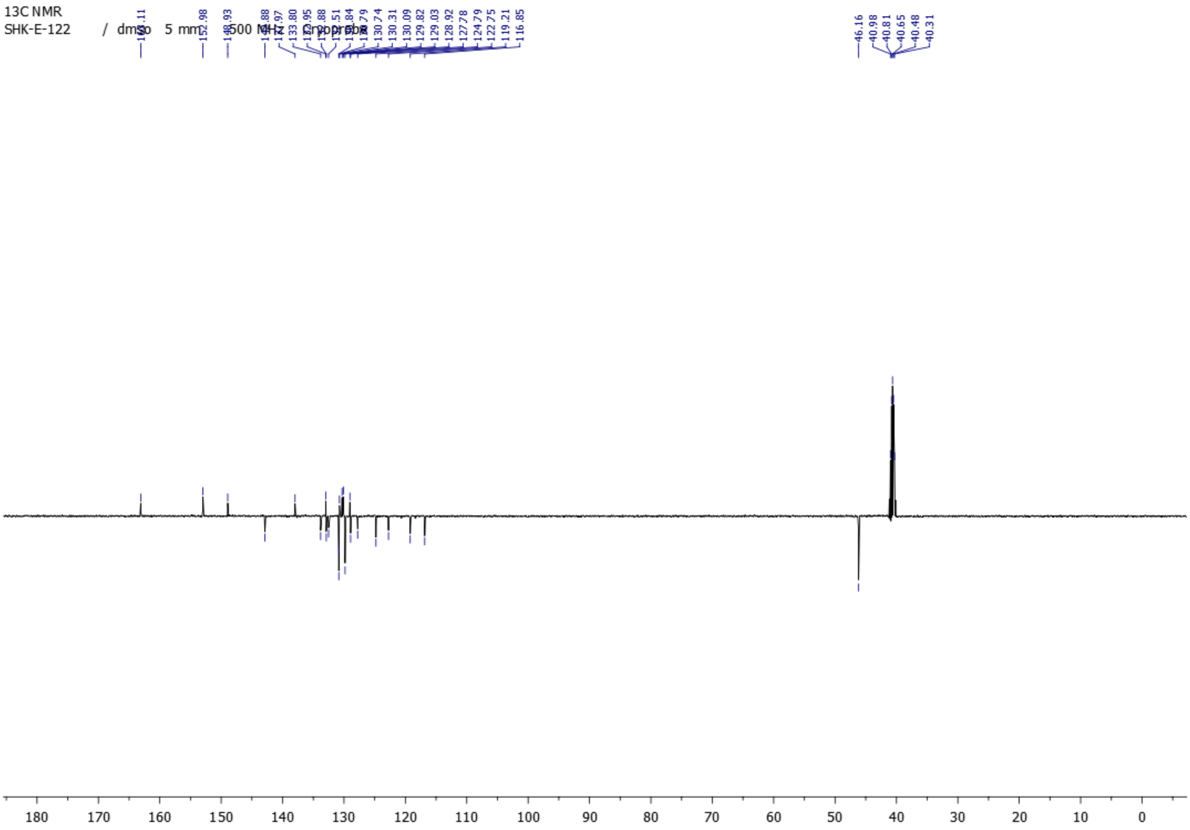

Figure S20. ¹³C NMR Spectrum of compound **5e**.

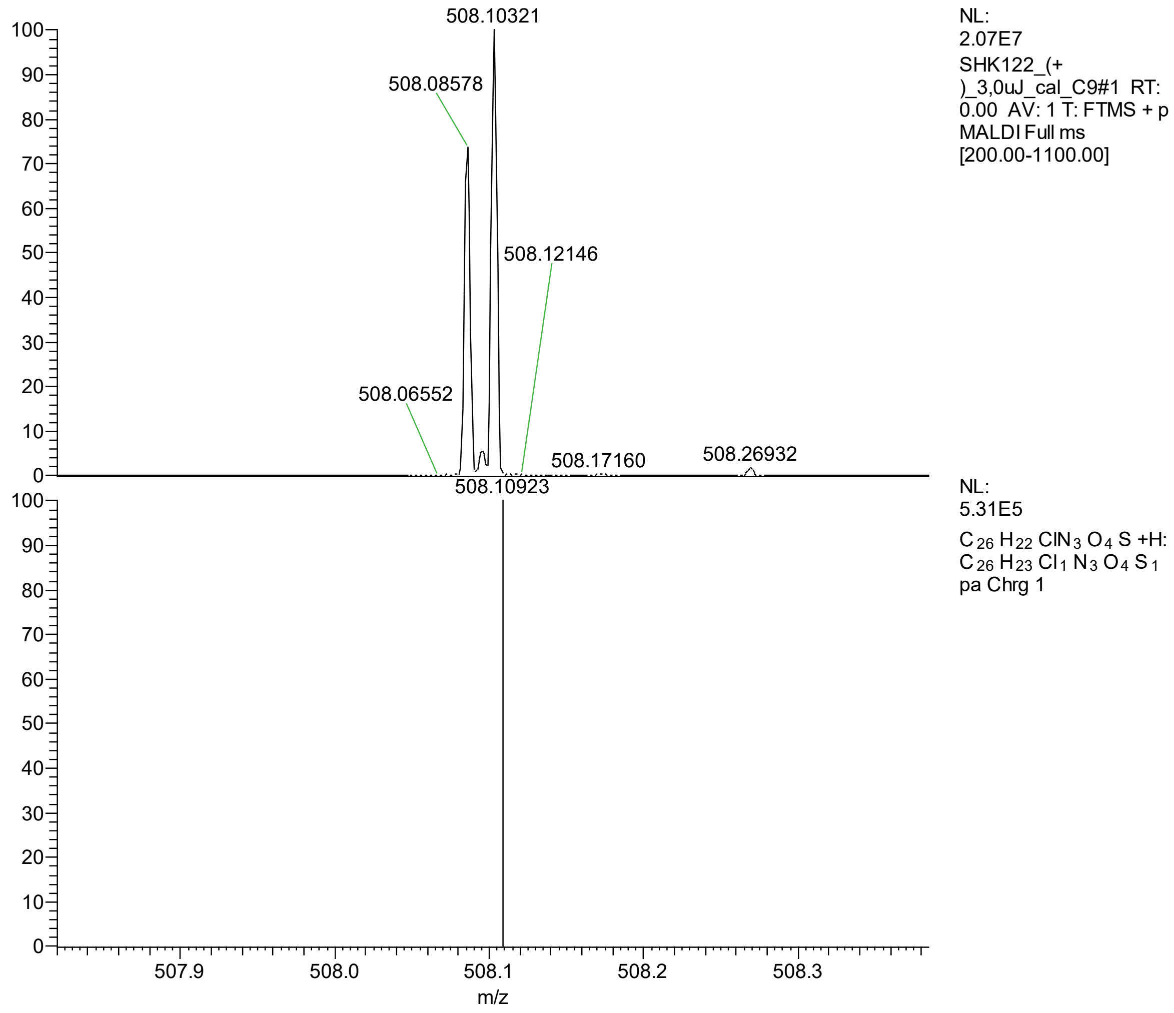

Figure S21. HRMS of compound **5e**.

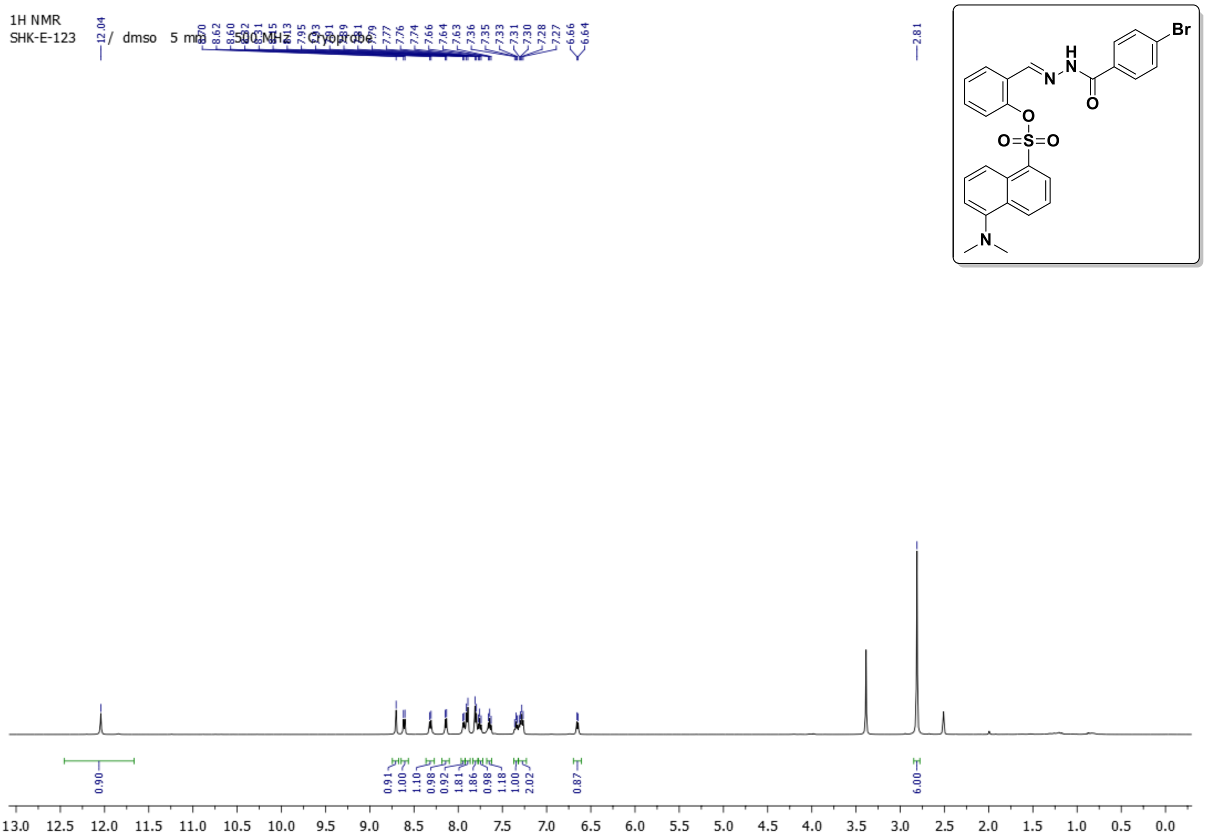

Figure S22. ^{1}H NMR Spectrum of compound **5f**.

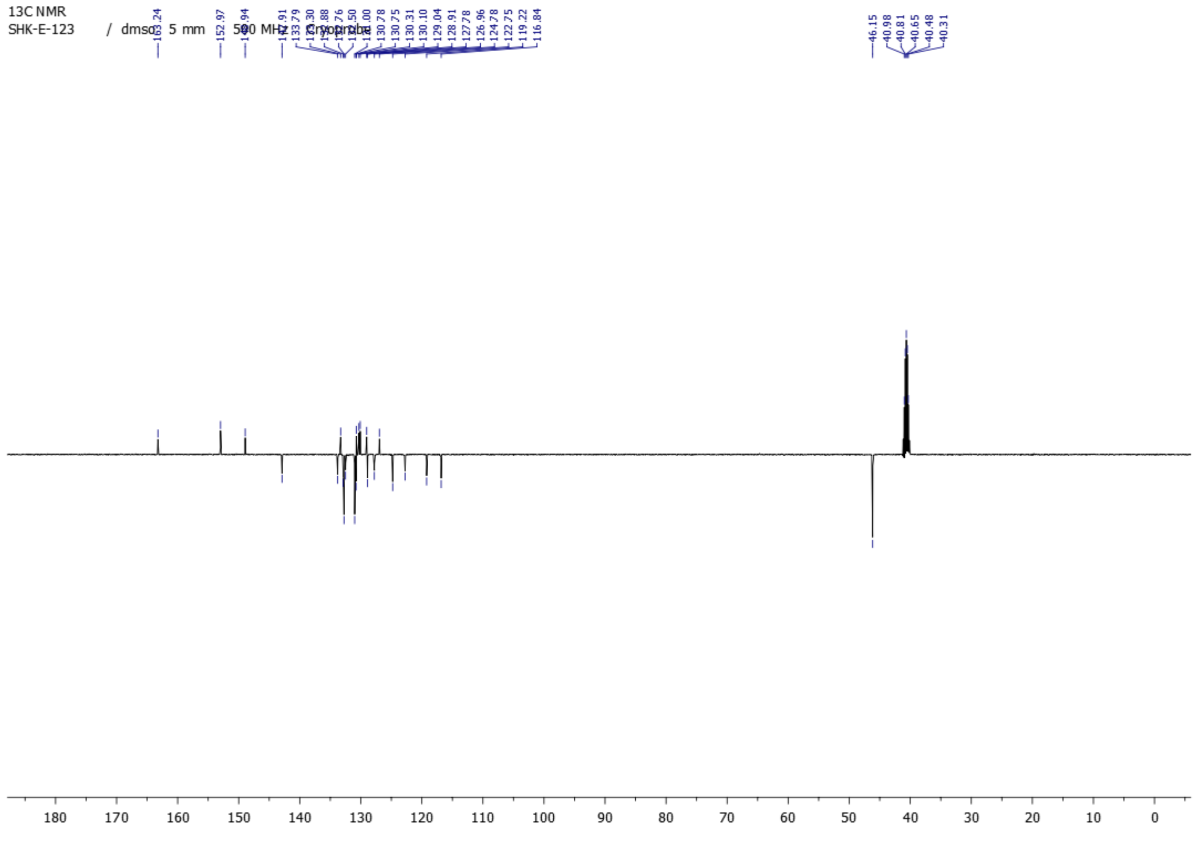

Figure S23. ^{13}C NMR Spectrum of compound **5f**.

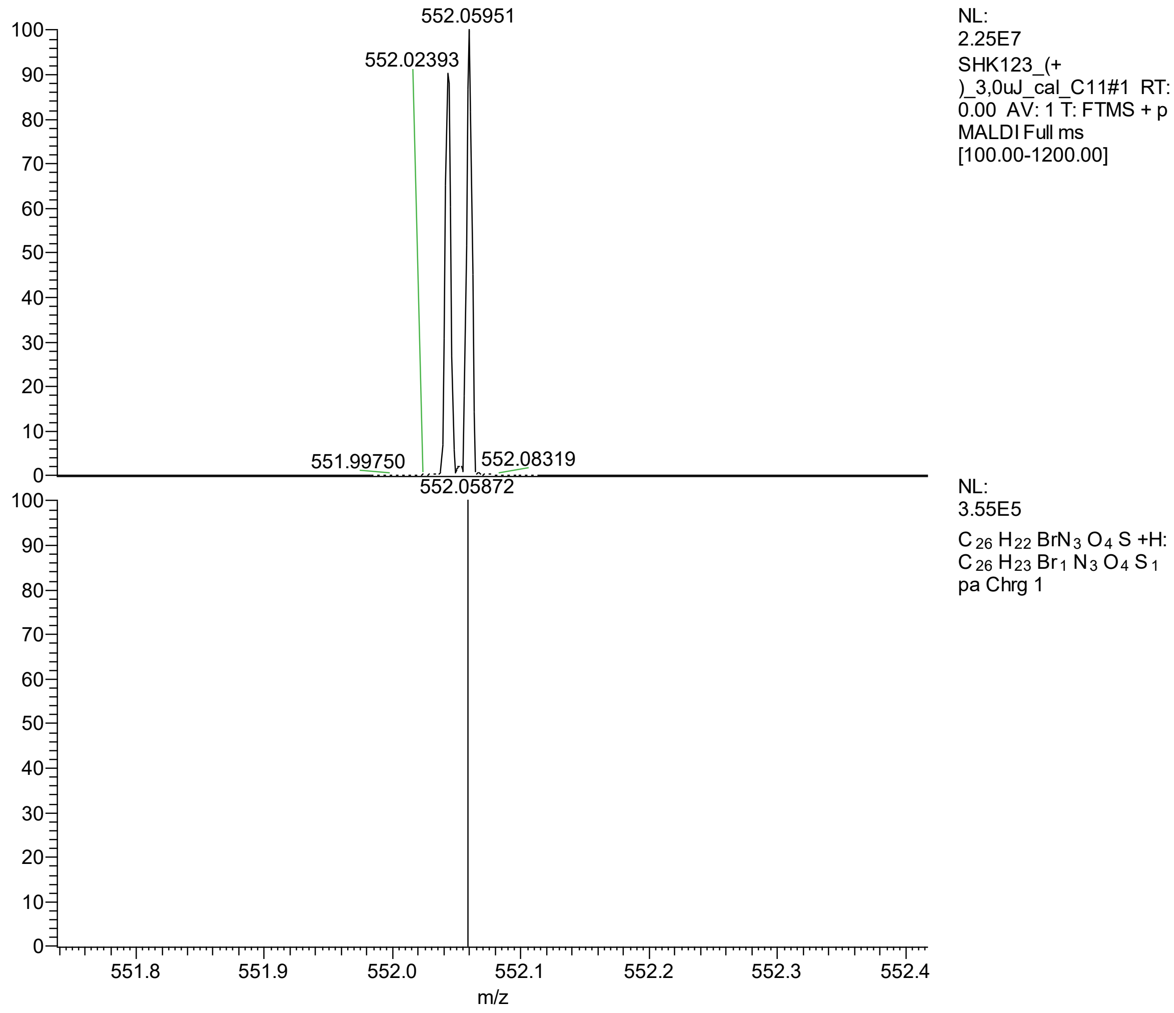

Figure S24. HRMS of compound **5f**.

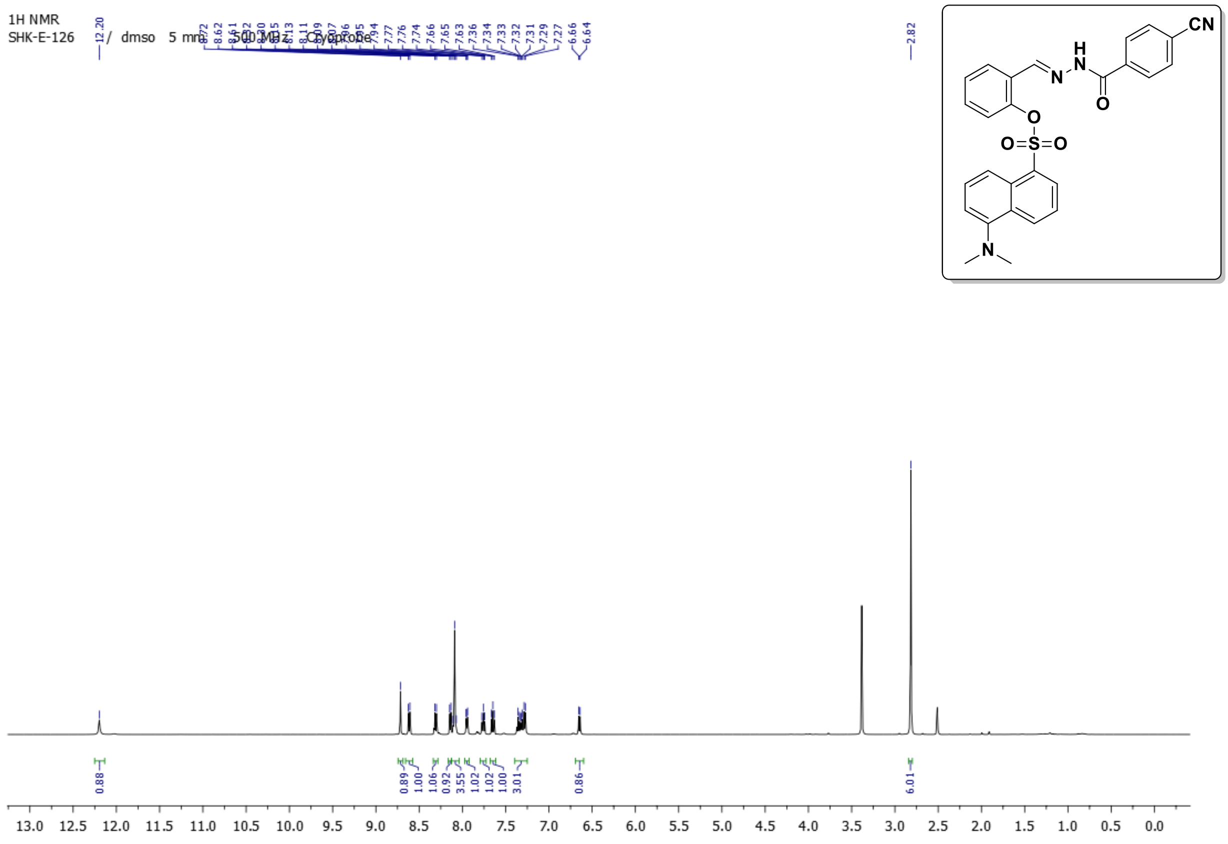

Figure S25. ^{1}H NMR Spectrum of compound **5g**.

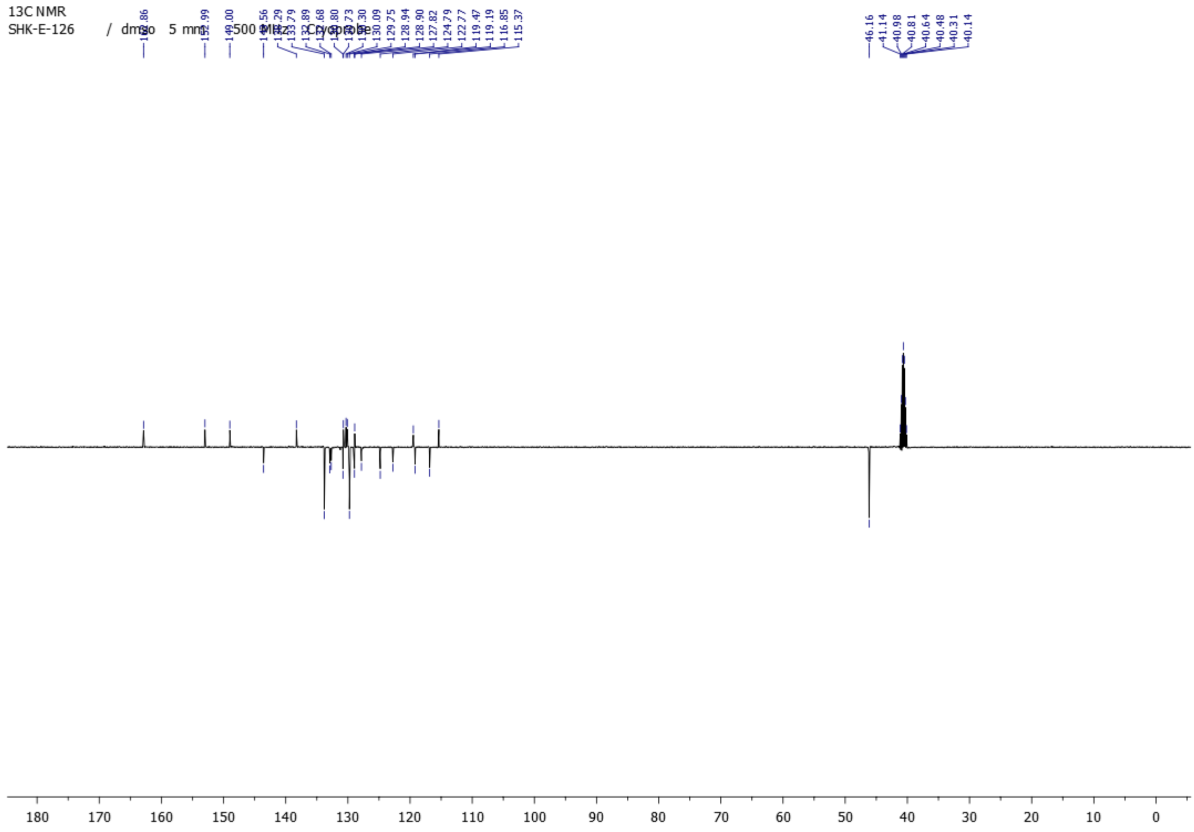

Figure S26. ^{13}C NMR Spectrum of compound **5g**.

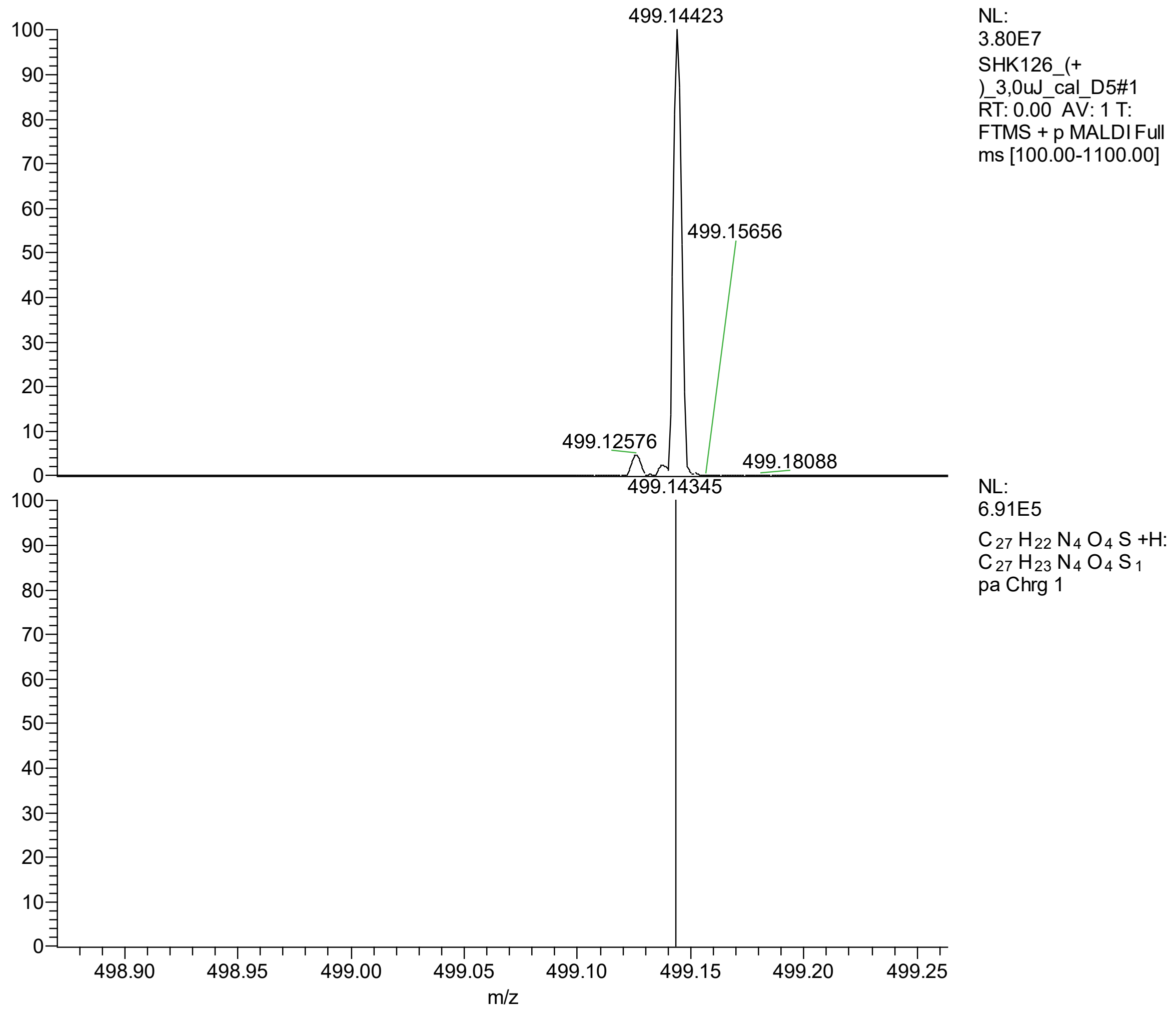

Figure S27. HRMS of compound **5g**.

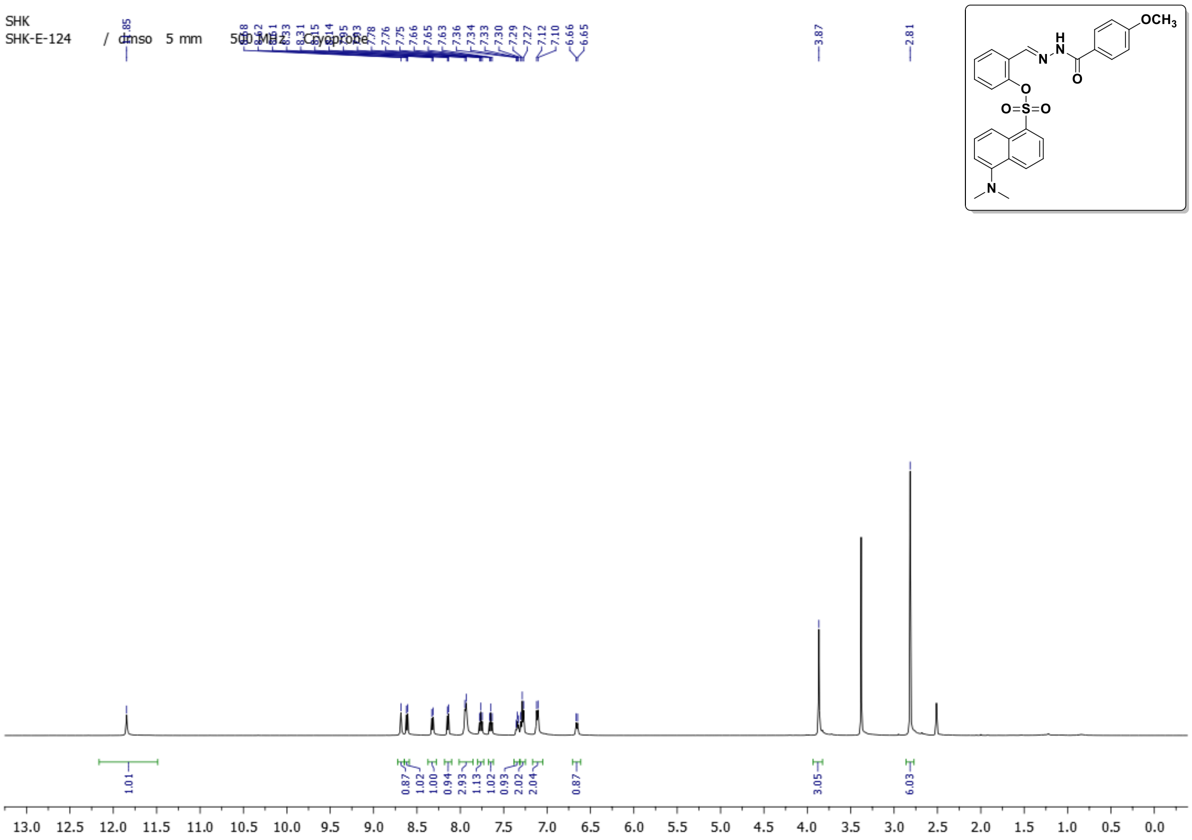

Figure S28. ^{1}H NMR Spectrum of compound **5h**.

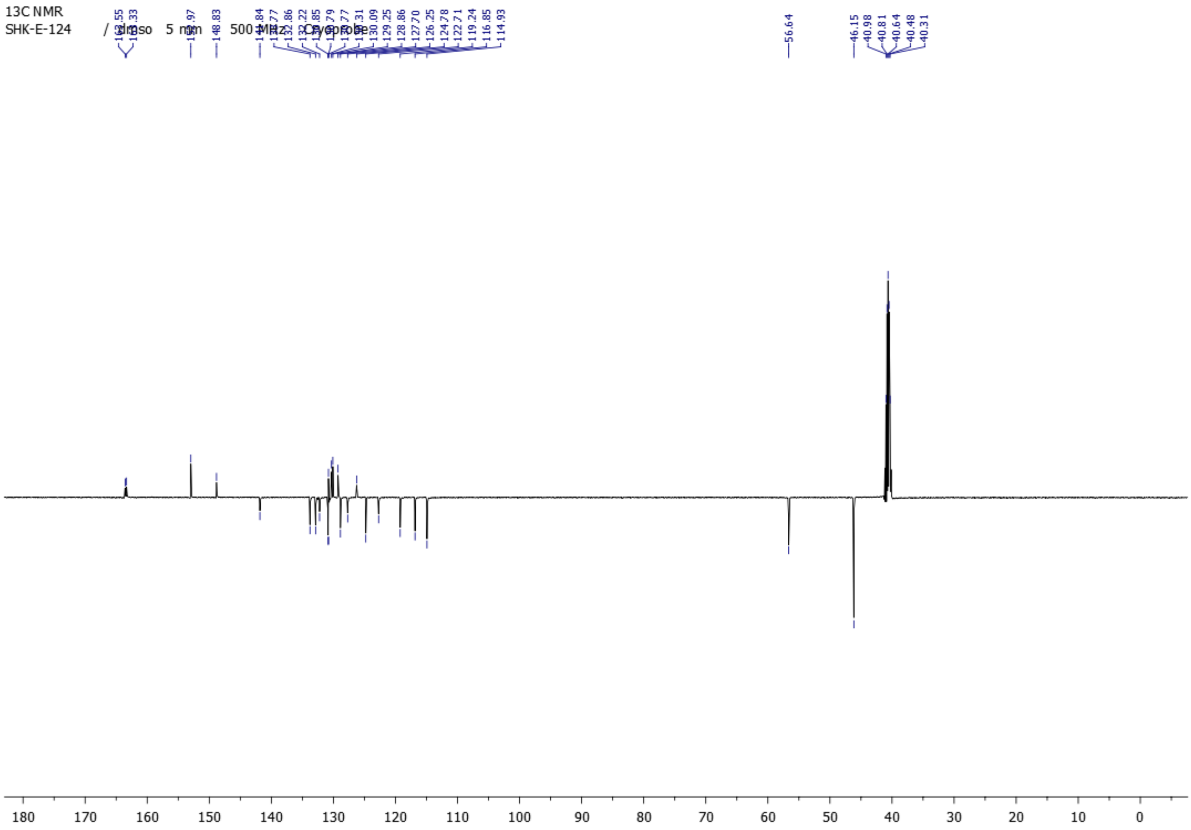

Figure S29. ^{13}C NMR Spectrum of compound **5h**.

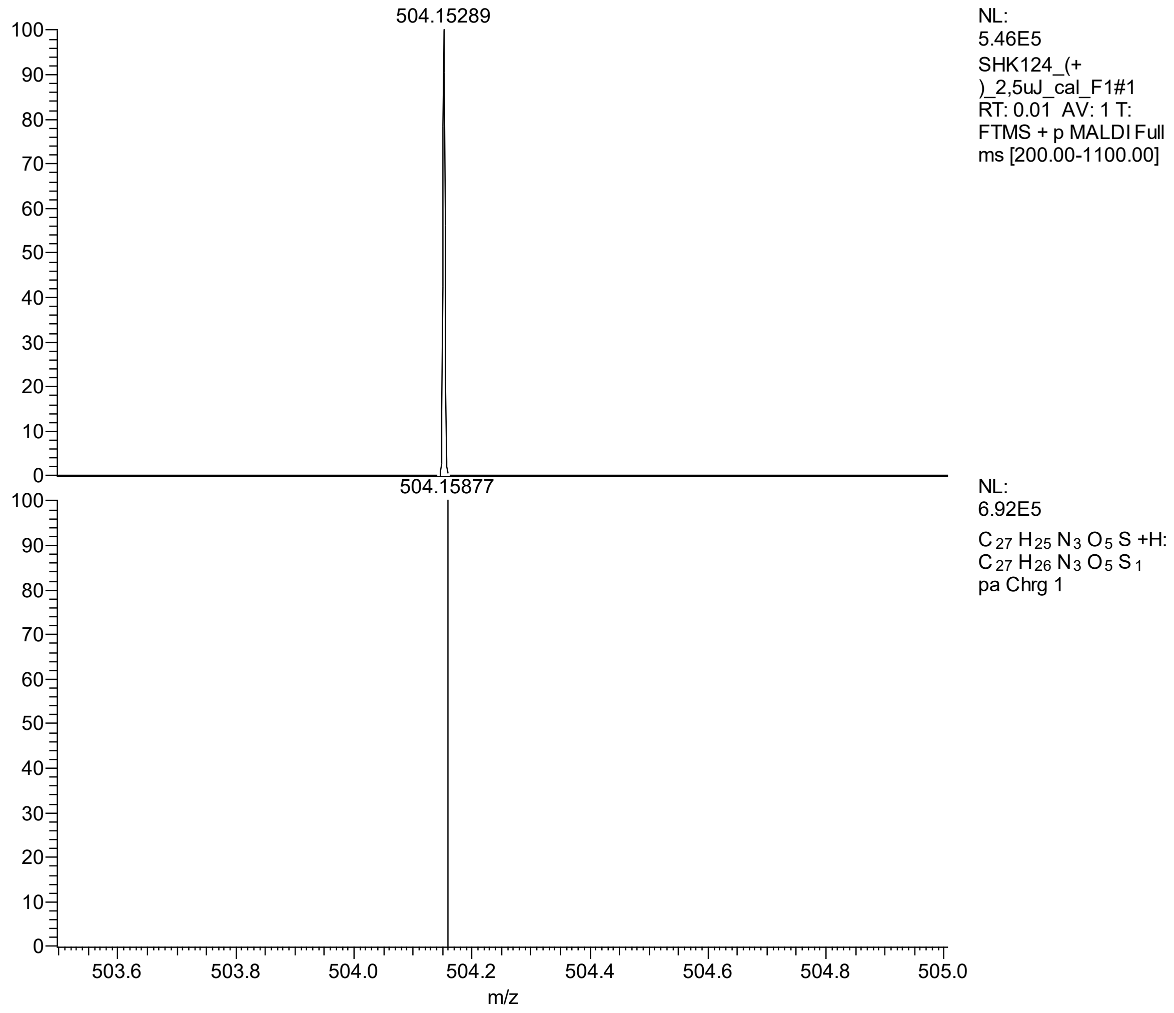

Figure S30. HRMS of compound **5h**.

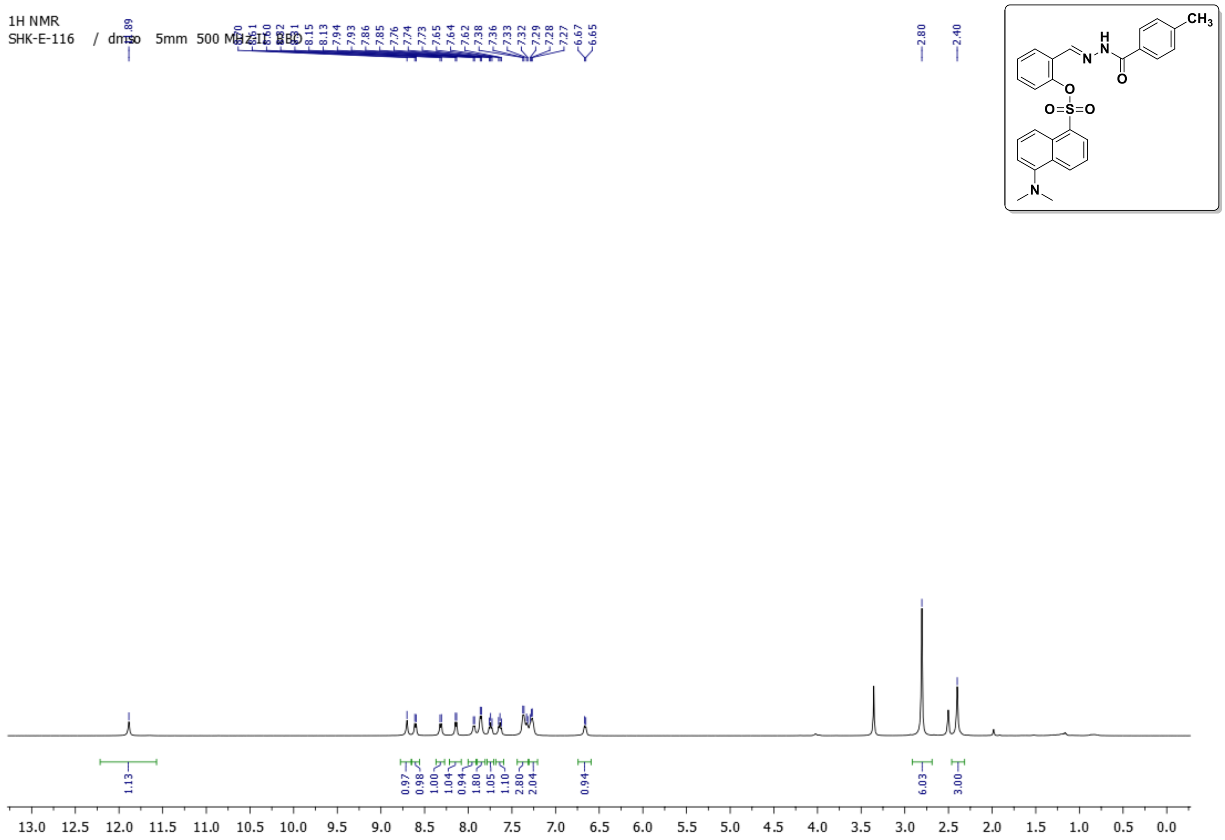

Figure S31. ^{1}H NMR Spectrum of compound **5i**.

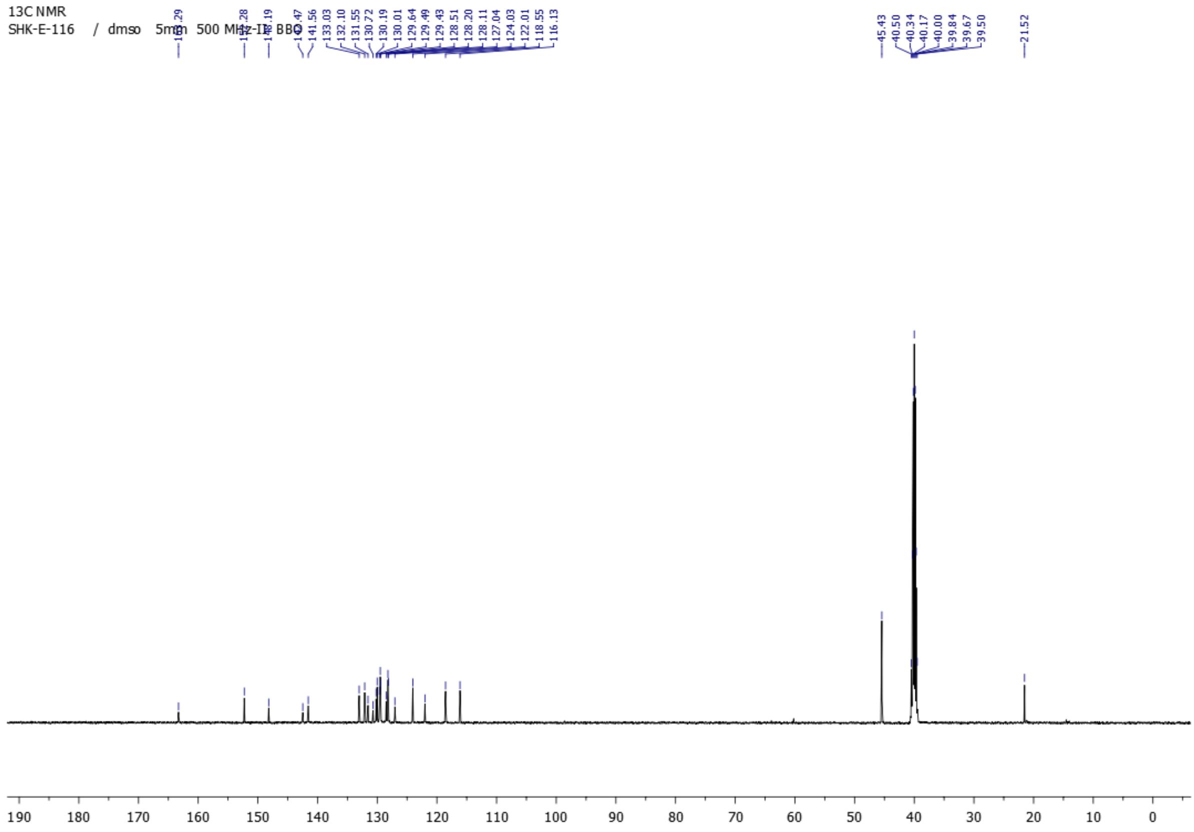

Figure S32. ^{13}C NMR Spectrum of compound **5i**.

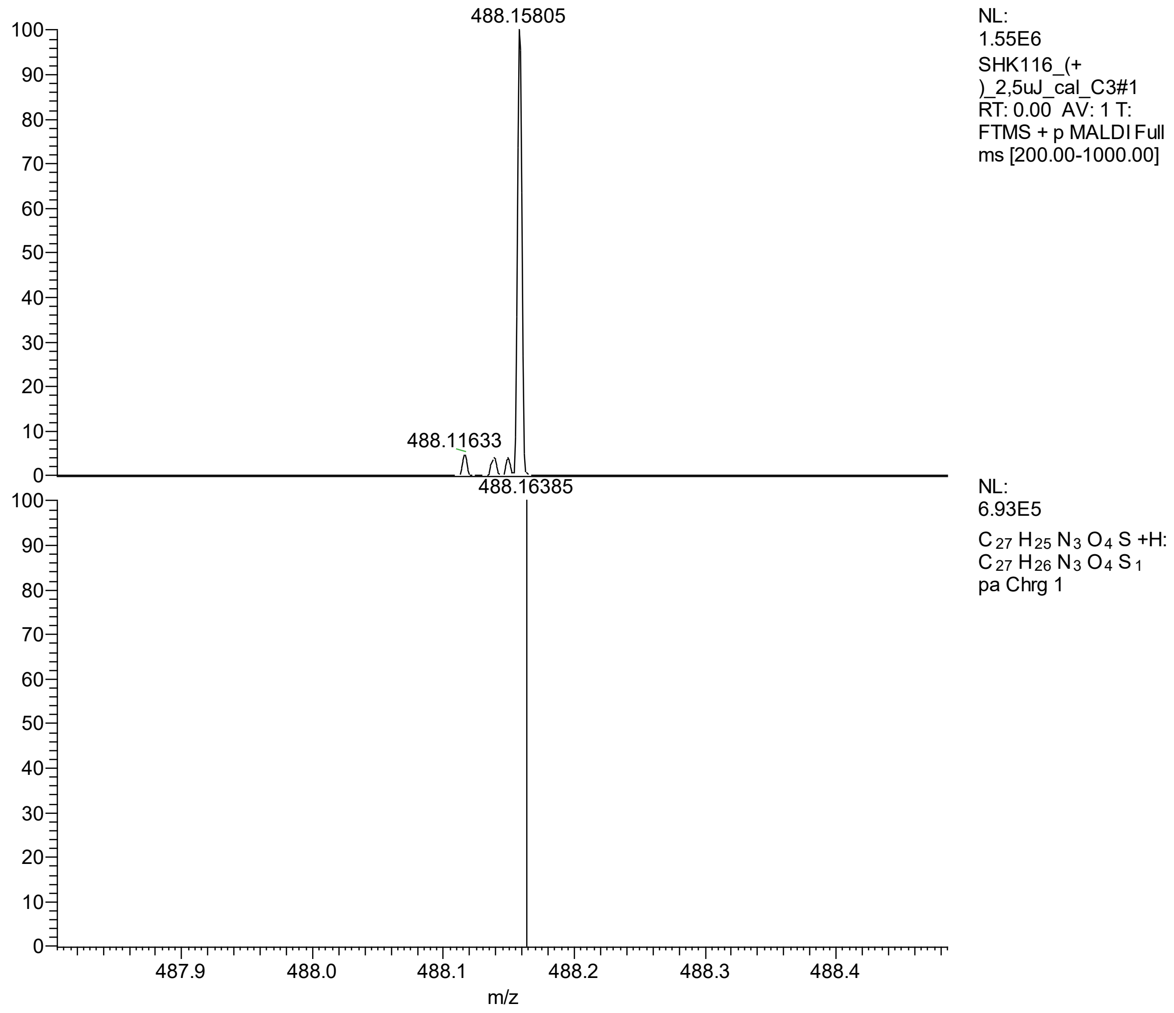

Figure S33. HRMS of compound **5i**.

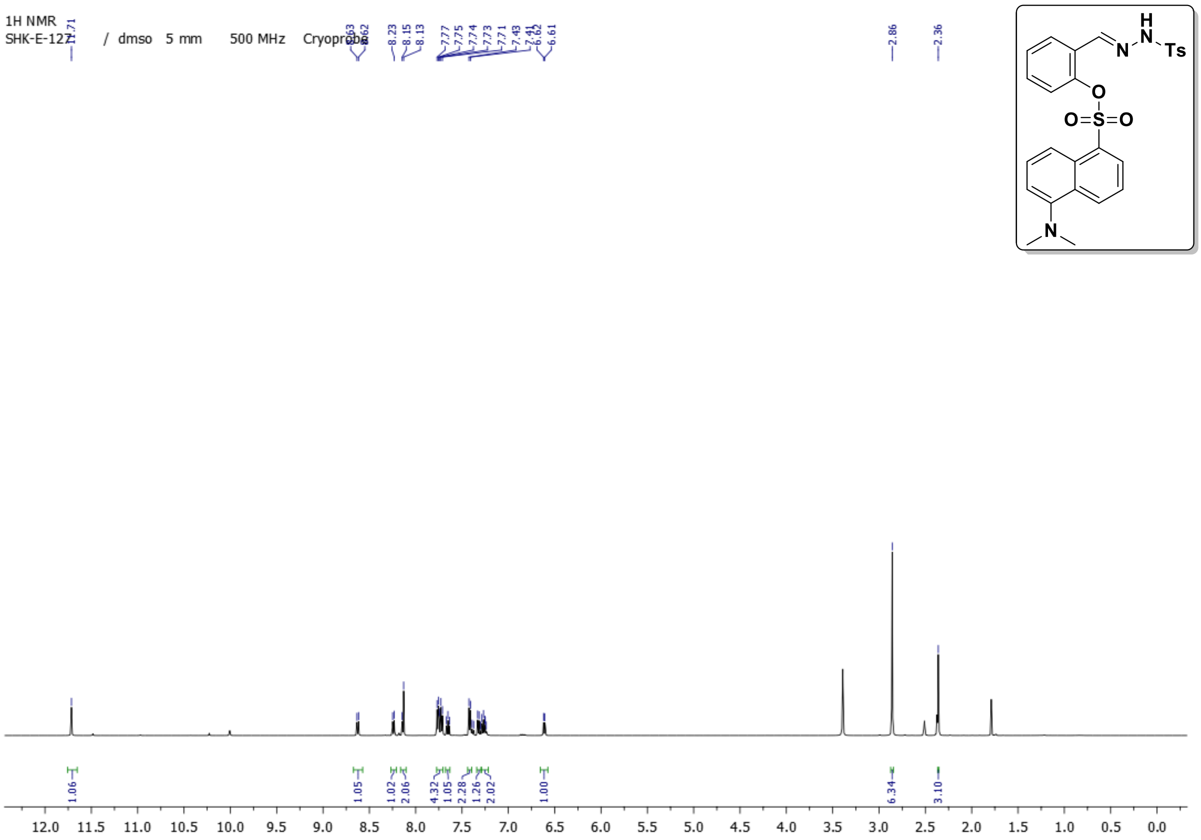

Figure S34. ^{1}H NMR Spectrum of compound **5j**.

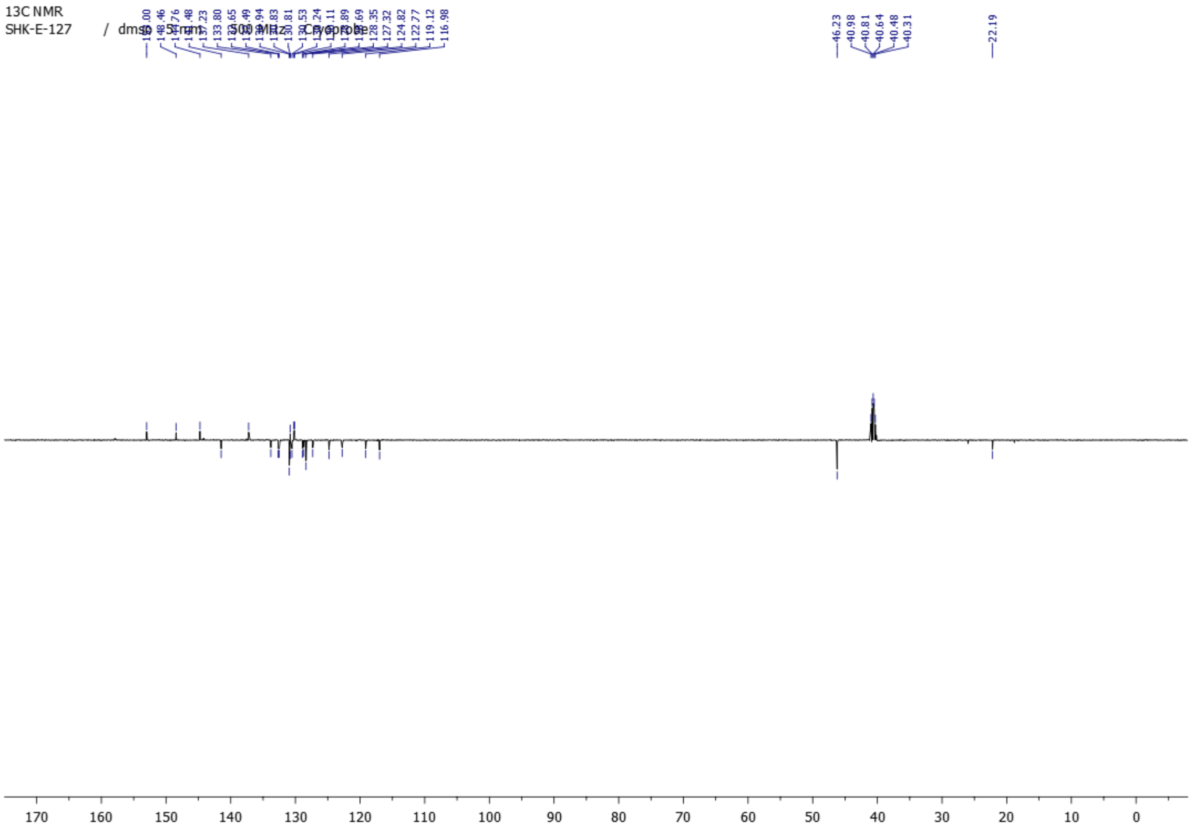

Figure S35. ^{13}C NMR Spectrum of compound **5j**.

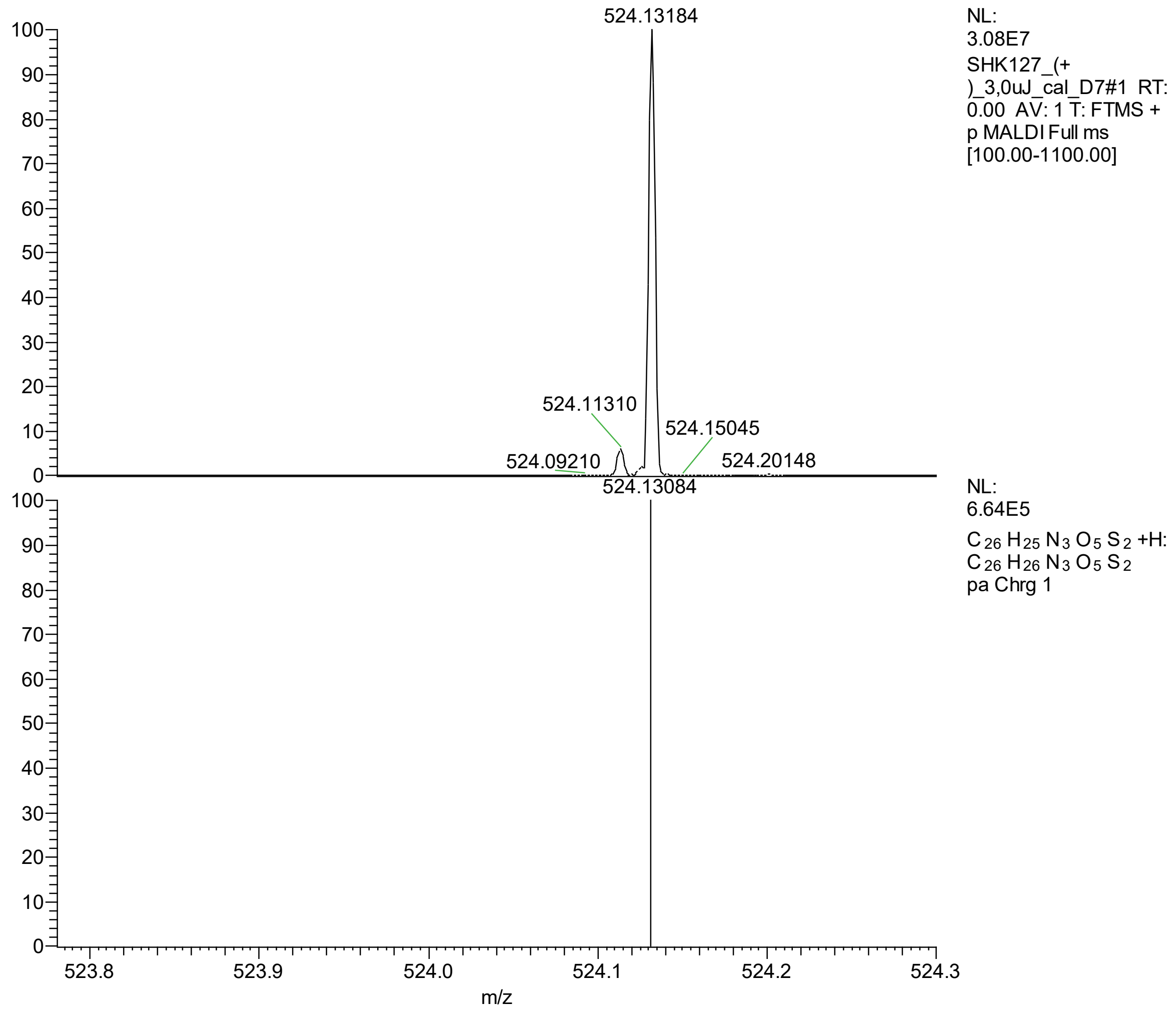

Figure S36. HRMS of compound **5j**.
